# The repeated evolution of anti-bat bioacoustics in hawkmoths

**DOI:** 10.1098/rspb.2026.2303

**Published:** 2026-11-04

**Authors:** Paul K. Masonick, Maja Petricic, Alessandro Giusti, Christian D. Couch, Jesse R. Barber, Akito Y. Kawahara

**Affiliations:** ^1^ Florida Museum of Natural History, University of Florida, Gainesville, FL, USA; ^2^ African Natural History Research Trust, Herefordshire, UK; ^3^ Insects Division, The Natural History Museum, London, UK; ^4^ McGuire Center for Lepidoptera and Biodiversity, Gainesville, FL, USA; ^5^ Department of Ecology and Evolutionary Biology, University of Colorado Boulder, Boulder, CO, USA; ^6^ American Museum of Natural History, New York, NY, USA

**Keywords:** convergent evolution, predator–prey interactions, phylogenetic comparative analysis, Lepidoptera, stridulation

## Abstract

Predator–prey interactions impose strong selective forces, often resulting in remarkable anti-predatory adaptations. Among insects, moths have been engaged in a long-standing arms race with bats, leading to the evolution of diverse anti-predator strategies. Here, we investigate the evolution of anti-bat stridulatory behaviour in hawkmoths (Lepidoptera: Sphingidae) by integrating acoustic recordings, high-resolution scanning electron microscopy and micro-computed tomography imaging and a time-calibrated phylogeny. We classify three distinct ultrasound-producing systems—scale-on-scale, scale-on-spine and scale-on-nodule—based on the interaction of scraper and file components. Building on previous descriptions, we uncover new details of two stridulatory mechanisms and describe for the first time the file component in the scale-on-nodule system of Macroglossinae. Phylogenetic analyses reveal that stridulatory responses to bat echolocation evolved independently multiple times among hawkmoths within the past approximately 30 million years. Furthermore, we demonstrate that divergent lineages produce acoustically distinct signals that correlate with variation in stridulatory morphology and the presence or absence of hearing organs. Collectively, our results highlight the repeated evolution of ultrasonic defences across divergent hawkmoth lineages and underscore predator–prey conflict as a powerful driver of both evolutionary innovation and redundancy in bioacoustic warfare.

## 1. Introduction

Predator–prey interactions exert powerful selective pressures, driving co-evolutionary arms races that have produced remarkable anti-predatory adaptations across the tree of life [1]. In arthropods, examples of similar phenotypes originating multiple times independently in response to predatory threats are prolific and evident in the replicate evolution of camouflage [2–4], Müllerian and Batesian mimicry [5–8], chemical defences [9–11] and a myriad of specialized behaviours [12–14]. Among insects, moths and butterflies (Lepidoptera) are particularly notable for the diversity of their defences, which reflect the wide range of predators they encounter [15].

Nocturnal Lepidoptera face intense selection from bats and have repeatedly evolved bioacoustic defences, including the detection of ultrasonic bat cries [16,17] and ultrasound production [18,19]. Anti-bat ultrasound can advertise toxicity [20], mimic unpalatable species [21], jam bat sonar [22] or startle naive bats as they strike their prey [23]. Many moths possess hearing organs tuned to detect bat echolocation [16,17,24–29], enabling evasive manoeuvres such as flight cessation or sudden turns [30,31], and, in some cases, the emission of sonar-disrupting ultrasound [22,32].

Hawkmoths (Sphingidae) comprise a diverse clade of approximately 1600 predominantly nocturnal species [33] and many species have evolved unique genital-based stridulatory mechanisms that enable them to evade bat attacks [34,35]. Ultrasonic stridulation is taxonomically widespread among male Sphingidae, occurring among the family's three most speciose subfamilies, Macroglossinae, Smerinthinae and Sphinginae. Some female Sphingidae are also known to stridulate [32], but the prevalence, mechanisms and functional roles of female-specific stridulation remain poorly documented. Among males of the more than 140 hawkmoth species tested thus far, over half emit ultrasound (77 out of 143; supplementary material, table S1) [32,36,37]. In some species, these signals not only overlap with the frequency range in ultrasound production of hunting bats but are also produced at high duty cycles (more than 20% of a given time window during which the moth is actively producing sound), a key factor to enable effective sonar jamming [32,38]. Stridulation appears to have evolved multiple times; however, the degree of independence among these origins remains unclear, as previous attempts to trace the evolutionary history of anti-bat sound production were hindered by sparse taxon sampling and small genetic datasets that did not robustly resolve key phylogenetic relationships [32].

The structures underlying sound production have been described for several members in the Smerinthinae and Sphinginae [15,34,39,40]. In these taxa, sound is produced when a patch of scraper scales on the male genital valve rubs against a row of heavily modified file scales, termed the ‘stridulatory curtain’ (*sensu* [41]), located along the posterior margin of the eighth abdominal tergite (T8). By contrast, the mechanism of sound production in Macroglossinae remains poorly understood. While valvular scraper scales have been reported for this subfamily [39,42], the complementary structures with which these scales interact to generate sound have not been clearly identified.

Variation in these stridulatory systems is probably to create differences in acoustic signal properties. Predator–prey interactions with echolocating bats may impose strong selective pressures on signal evolution because variation in bat hunting strategies, echolocation call structure, sensory processing and prey capture behaviour, including differences in attack kinematics and terminal buzz structure, may favour defensive signals that are effective under different acoustic and temporal constraints [43,44]. In eared hawkmoths, auditory organs enable the detection of approaching bats and the initiation of defensive behaviours including ultrasound production [27,28,32], providing the sensory basis for selection to act on signal characteristics that reduce predation risk. For example, signals with higher duty cycles are predicted to be more effective at interfering with bat sonar [32,36]. We therefore hypothesize that evolutionary modifications of stridulatory structures drive variation in acoustic output and that lineages possessing auditory organs are more likely to evolve signal properties associated with effective sonar interference. Despite their potential importance, the composition, functional roles and evolutionary convergence and divergence of stridulatory components and acoustic traits in hawkmoths remain largely unexplored.

The objectives of this study were to: (1) identify independent origins of (A) anti-bat acoustic responses and (B) stridulatory systems of male Sphingidae; (2) describe in greater depth the unique acoustic systems of hawkmoths using scanning electron microscopy (SEM), computed tomography (micro-CT) imaging and existing videography; (3) test whether divergent hawkmoth lineages make different sounds, and whether these differences correlate with variation in stridulatory structures and the presence or absence of bat detecting ears; and (4) examine the morphological diversity found across the scraper and file components of these stridulatory systems. Our study integrates both published and unpublished acoustic recordings, high-resolution SEM and micro-CT imaging data and a well-supported, time-calibrated phylogeny of Sphingidae. We classify and describe the mechanics of three distinct stridulatory systems in Sphingidae—termed here as scale-on-scale, scale-on-spine and scale-on-nodule—based on the interaction between the scraper and file components involved in sound production. While the scale-on-scale and scale-on-spine systems have been described for select taxa [34,39,40], we provide a deeper understanding on how these mechanisms work and reveal, for the first time, both the scraper and file components of the scale-on-nodule system of the Macroglossinae. Our analyses show that stridulatory responses to bat echolocation have probably evolved independently multiple times over the past 5–30 million years, and that the acoustic properties of these signals are closely linked to the motion and morphological structure of the stridulatory organ. Collectively, our findings deepen our understanding of defensive bioacoustic adaptations in this iconic predator–prey system.

## 2. Material and methods

### (a) Behavioural assays

To link morphology with functional and evolutionary patterns and uncover the origins of anti-bat acoustic responses across Sphingidae (objective 1A), we first conducted behavioural experiments on tethered, flying hawkmoths to test whether individuals respond with ultrasonic stridulation to bat call playback and/or tactile stimuli (supplementary material, tables S1–S3). We report new behavioural observations of hawkmoth stridulatory responses obtained during recent field expeditions (supplementary material, table S2) and combine them with those published before [32,36,37].

All assays were conducted after dark to elicit the most natural anti-bat behaviours. Each moth was tethered in flight using a monofilament fishing line looped (‘lasso’ technique) around the junction of the thorax and abdomen and threaded through a hollow plastic rod secured in forceps (see [32]). While tethered, all moths were tested for two types of defensive responses: (i) ultrasound production in response to bat call playback and (ii) ultrasound production in response to simulated predator handling. We first simulated bat attack using six recorded bat echolocation attack sequences [36]. As bats use different echolocation strategies around the world, all moths, independent of their location, were queried with three different frequency-modulated (FM) echolocation attack recordings, two constant-frequency (CF) attacks and a synthetic call. Although these recordings originated from bat species that are not always sympatric with the moth species we tested, the sequences we tested were biologically relevant because the fundamental characteristics of FM and CF echolocation strategies are largely conserved across bats (see [36] for details). Moths were exposed to recordings of bat echolocation sequences broadcast through an Avisoft UltraSoundGate Player BL Pro speaker/amplifier (±6 dB, 20–110 kHz, playback sampling rate 250 kHz). The speaker was positioned on-axis 10 cm in front of the head owing to their mouthpart-based hearing organs. Any sounds emitted by the moths were recorded with an Avisoft CM16 condenser microphone (±3 dB, 20–140 kHz) positioned 10 cm from the posterior end of the moth's abdomen and attached to an UltraSoundGate 116Hme data acquisition device sampling at 375 kHz via a laptop computer running Avisoft Recorder software.

Following ultrasound playback trials, tactile trials were conducted to elicit potential stridulation from both hearing and deaf taxa and record signals suitable for downstream measurement and analysis, without the confounding effects of overlapping bat sonar. To simulate handling by a predator, each moth was gently compressed with fingers at the head, thorax or abdomen to prompt stridulation. We recorded all responses to bat call playback and handling (supplementary material, table S2); the former was coded into a trait matrix for ancestral state reconstruction (ASR) (supplementary material, table S3; see §2c).

### (b) Specimen dissection, imaging and stridulatory system characterization

Dissections of male genitalia from known and putative ultrasound-producing taxa, as well as non-stridulating taxa, were conducted to investigate the evolution of stridulatory systems across hawkmoths (objective 1B) and characterize these mechanisms in greater detail (objective 2). In total, we dissected males from 124 species spanning all four subfamilies of Sphingidae (supplementary material, table S4). Although some female Sphingidae are known to produce ultrasound [32], the prevalence and characteristics of female-specific stridulation remain insufficiently documented; therefore, our analyses focused exclusively on males. Specimens from both the McGuire Center for Lepidoptera and Biodiversity at the University of Florida (MGCL) and the Natural History Museum, London (NHMUK) were examined for this study. Habitus images of specimens examined from the MGCL are provided in the appendix in the supplementary material (figures S1–S166). Taxonomic designations follow Kitching *et al.* [33].

Scraper scales, when present, were dissected and separated from valvae and oriented with the lateral, external surface facing upward. Scales were imaged in planar view under a Zeiss AxioZoom v16 stereo light microscope at subfamily-specific magnifications (approx. 40× magnification for Macroglossinae, approx. 60× magnification for Sphinginae and approx. 1000× magnification for Smerinthinae). For each scale, 15–20 slices were acquired and subsequently stacked using the Helicon Remote (v3.8.4 W) and Helicon Focus (v6.7.1) software.

SEM of the dissected stridulatory organs was also conducted. Male valvae and the T8 were mounted, without coating, on aluminium stubs using double-sided carbon tape. Imaging was conducted with a Hitachi Tabletop Microscope TM4000II at multiple magnifications to document overall morphology (i.e. presence/absence of stridulatory organs) and reveal finer details of scale morphology and surface microsculpturing.

To visualize the relative *in situ* positions of the scraper and file components across different stridulatory systems, one representative species from each subfamily was scanned using a Phoenix v|tome|xm micro-CT system (GE Sensing & Inspection Technologies GmbH, Wunstorf, Germany) at the University of Florida, Gainesville, FL, USA. For each specimen, the abdomen was removed and fixed to a carbon rod to stabilize and position it within the scanner. All scans were acquired by taking 1000 projections over a full 360° rotation. Following reconstruction, volumetric data were imported into 3D Slicer v5.6.2 for segmentation and three-dimensional visualization.

For this study, a scale patch was designated as relevant to stridulation if it met two criteria: (i) scraper scales of interest exhibited a distinct contrast in their shape and morphology relative to adjacent valvular scales and (ii) the patch was positioned in direct contact with another area of relevant scales, spines or specialized microsculpturing on the T8 (the file apparatus). We report the presence or absence of male genital-based stridulatory organs and their specific condition (scale-on-scale, scale-on-spine or scale-on-nodule) in supplementary material, table S5.

### (c) Ancestral state reconstruction of ultrasonic response to bats and stridulatory systems

The timing and origin of bioacoustic trait evolution (objectives 1A and 1B) were investigated within a phylogenetic context by mapping trait changes across the most complete dated phylogeny of Sphingidae available [45] using ASR. This tree includes the most extensive sampling of Sphingidae to date with 310 hawkmoth species represented from all four subfamilies. We conducted two analyses on discrete traits using the stochastic character mapper function make.simmap in the *phytools* v2.3.0 R package [46]. Both analyses employed a symmetrical evolutionary model and were run for 1000 simulations each.

In the first analysis, we mapped stridulatory responses of moths to bat call playback. Moths that exhibited no response to these stimuli were coded as 0 (absent), whereas those that did respond with ultrasound production were coded as 1 (present) (supplementary material, table S3). To ensure the most complete trait matrix possible, species lacking response data were coded based on congeners that have been tested. For example, although *Xanthopan morganii* has not been tested directly, we scored it as responsive based on the report of *Xanthopan praedicta* responding to bat call playback [37]. This approach was only applied when a genus was recovered as monophyletic in the dated tree. Taxa for which no score could be assigned (e.g. because no congeners were tested or the genus was non-monophyletic) were coded as unknown with a value of 0.5 in the trait matrix and we allowed the stochastic mapping algorithm to estimate their condition.

The second analysis reconstructed the evolution of the stridulatory systems in male hawkmoths. Based on dissections conducted in the present study of both sound and non-sound producing species (supplementary material, table S4), four discrete character states were coded: 0 = stridulatory system absent, 1 = scale-on-scale, 2 = scale-on-spine and 3 = scale-on-nodule (supplementary material, table S6). Species that were not dissected, or that already had detailed morphological descriptions published, were assigned character scores consistent with those of their dissected congeners. For para- or polyphyletic genera, non-dissected species were coded as unknown, with equal probabilities (0.25) across the four character states, thus permitting make.simmap to approximate their condition.

### (d) Acoustic data processing, clustering and analysis

Differences in the sounds produced by divergent hawkmoth lineages (objective 3) were assessed by measuring and analysing acoustic variables from stridulatory responses recorded during tactile trials. Our acoustic analyses concentrated on the duration of a single complete modulation cycle of sound, defined as the two-part structure of the sound emissions. Using Avisoft SASLab Pro, we measured three modulation cycles per individual, except when only two cycles were available. Temporal characteristics of signals, including duty cycle defined as the proportion of a time window during which moth sound was present, modulation cycle duration and durations of individual modulation cycle components, were quantified using the pulse-train analysis tool with the following settings: time constant = 0.025 ms, threshold = 0.15 V, hysteresis = 15 dB, start/end threshold = 15 dB, envelope = rectification + exponential decay and pulse detection = peak search with hysteresis.

Spectral characteristics were quantified using the Power Spectrum (averaged) tool in Avisoft-SASLab Pro. Dominant frequency and frequencies 15 dB above and below the dominant frequency were extracted using a Hann evaluation window and a fast Fourier transform size of 1024. In total, we extracted from the recordings 11 acoustic parameters for analysis: (i) duty cycle of a single modulation cycle, (ii) duration of modulation cycle, (iii) duration of first half of the modulation cycle, (iv) duration of silent interval, (v) duration of second half of the modulation cycle, (vi) dominant frequency, (vii) dB of the dominant frequency, (viii) frequency at +15 dB above dominant frequency, (ix) frequency at −15 dB below dominant frequency, (x) peak-to-peak mean, and (xi) duty cycle of a 100 ms window of sound (supplementary material, table S7).

We used Uniform Manifold Approximation and Projection (UMAP) [47], a high-dimensionality reduction algorithm, to facilitate the comparison and visualization of the acoustic data in two-dimensional space. The 11 acoustic features measured from the ultrasound recordings (supplementary material, tables S7 and S8) were analysed using the R implementation of *umap* [48] to reduce dimensionality and visualize patterns among samples. Missing values were imputed using the median value of each parameter. For acoustic data clustering, the random seed was set to 15.

Differences among the three stridulatory systems were assessed using three key acoustic features selected to capture complementary aspects of signal structure: duty cycle of a 100 ms window of sound, mean dominant frequency and mean duration of modulation cycle (supplementary material, table S7). These variables were chosen because they capture the primary dimensions of variation among stridulatory signals while minimizing redundancy among highly correlated acoustic measurements. To statistically compare between groups (stridulatory system category), we applied pairwise *t*-tests using the ‘stat_compare_means’ function of the *ggpubr* package [49].

### (e) Morphology of scraper and file stridulatory components

A major aim of this study was to describe the scale and file components of these various systems in greater detail (objective 4). From images captured of scraper scales, four size measurements of individual scales were quantified in ImageJ v1.54 [50]: (i) the total outline length = perimeter (mm), (ii) surface area = aspect ratio (mm²), (iii) density = scales per mm², and (iv) logged scale size = ‘LogCentroidSize’ (supplementary material, table S9). Similarly, measurements of microsculpturing on the ventral surface of T8 were taken from SEMs with Fiji (ImageJ2) [51]. In this case, diameters of 30 nodules per specimen were measured to calculate mean nodule diameter per specimen (supplementary material, table S10). Microsculpturing was present on the ventral surface of T8 on all of the hawkmoths that we dissected. However, only among the Macroglossinae that possess enlarged valvular scales did we observe the organization of this microsculpturing into large, evenly spaced nodules.

To assess whether variation in scraper scale size can distinguish taxonomic groupings, we performed multivariate analyses of variance (MANOVA) followed by discriminant function analyses (DFA) in IBM SPSS Statistics v29.0.2.0 for classification at both subfamily and subtribe levels. The discriminant function achieved a high overall classification accuracy at the subfamily level, correctly assigning 81.3% of specimens, but performed worse when assessing Macroglossinae at the subtribe level, with only 43.4% of cases correctly classified (supplementary material, tables S11 and S12). The first two canonical functions accounted for most of the morphological variation between subfamilies, with perimeter emerging as one of the strongest predictors of subfamily classification alongside logged scale size (Wilk's lambda = 0.044, *p* < 0.001).

Phylogenetic generalized least squares (PGLS) regression was conducted using the *nlme* [52,53] and *ape* [54] packages in R to account for phylogenetic non-independence among species in trait comparisons. First, we modelled the expected covariance among taxa under a Brownian motion model of trait evolution and fit a PGLS model testing the relationship between scraper scale perimeter (the total outline length in mm) and nodule diameter (in µm). These variables were selected for comparison because they serve as proxies for the size of relevant structures involved and exhibit considerable variation across dissected taxa. For scraper scales, the perimeter was prioritized as the key trait owing to its strong discriminatory power in prior multivariate analyses. This analysis was performed on mean-per-genus scale perimeters and mean-per-genus nodule diameters (supplementary material, table S13).

Lastly, we visualized the evolution of two continuous traits using the ‘contMap’ function from the *phytools* v 2.3.0 R package [46]. Specifically, we mapped mean-per-genus perimeters of scraper scale and mean-per-genus nodule diameters (supplementary material, table S13) across a pruned, genus-level dated hawkmoth tree of Couch *et al.* [45]. For each analysis, ancestral states were estimated using a maximum likelihood approach (method = ‘anc.ML’), and trait values were displayed as colour gradients.

## 3. Results and discussion

We constructed extensive behavioural and morphological datasets in combination with the most comprehensive phylogeny of hawkmoths currently available, comprising 310 ingroup species, to investigate the evolutionary origins of anti-bat bioacoustics in Sphingidae. These data were collected over the past decade from populations sampled worldwide and include more than 140 hawkmoth species, of which 77 were confirmed to emit ultrasound (supplementary material, table S1).

### (a) Evolution of anti-bat acoustic responses

First, we mapped the presence or absence of stridulatory response to bat call from behavioural assays (supplementary material, tables S2 and S3) across the dated hawkmoth phylogeny of Couch *et al.* [45] using ASR to examine the behaviour's evolution in a phylogenetic context. We found that most hawkmoths that possess hearing organs (comprised a pilifer + palp system in Sphingidae [28]; see [32] for a dissection-based inventory of taxa confirmed to have ears) respond to bat call playback by emitting their own ultrasound. ASR of stridulatory response estimated that there are multiple origins of the behaviour across Sphingidae (figure 1A). The origin of laryngeal echolocation in bats (denoted by the brown arc at approximately 50 mya in figure 1) [43,55] predates the origins of the sphingid subfamilies by roughly 5–15 million years and occurred long before any ancestral hawkmoths were estimated to have exhibited this anti-predatory behaviour. There appear to have been three separate origins of stridulation in response to bat echolocation within the Macroglossinae: twice among Macroglossini and once among Dilophonotini (figure 1A, blue arrows). Among these, *Nephele* and *Pseudosphinx* are two genera for which hearing organs have not been described, yet they respond strongly to echolocation playback. Multiple instances of this behaviour may also have arisen within the Sphinginae (figure 1A, blue arrows). Among the 27 species of Sphinginae that were tested, male *Psilogramma* (*Psilogramma* genus group), *Megacorma* (Acherontiina), *Xanthopan* (Cocytiina) and *Meganoton* (Sphingina) all responded to bat call playback (supplementary material, table S1). Smerinthinae, a clade not known to possess hearing organs, did not respond to echolocation playback; however, roughly half of the 31 species tested did stridulate in response to tactile stimuli.

**Figure 1.**
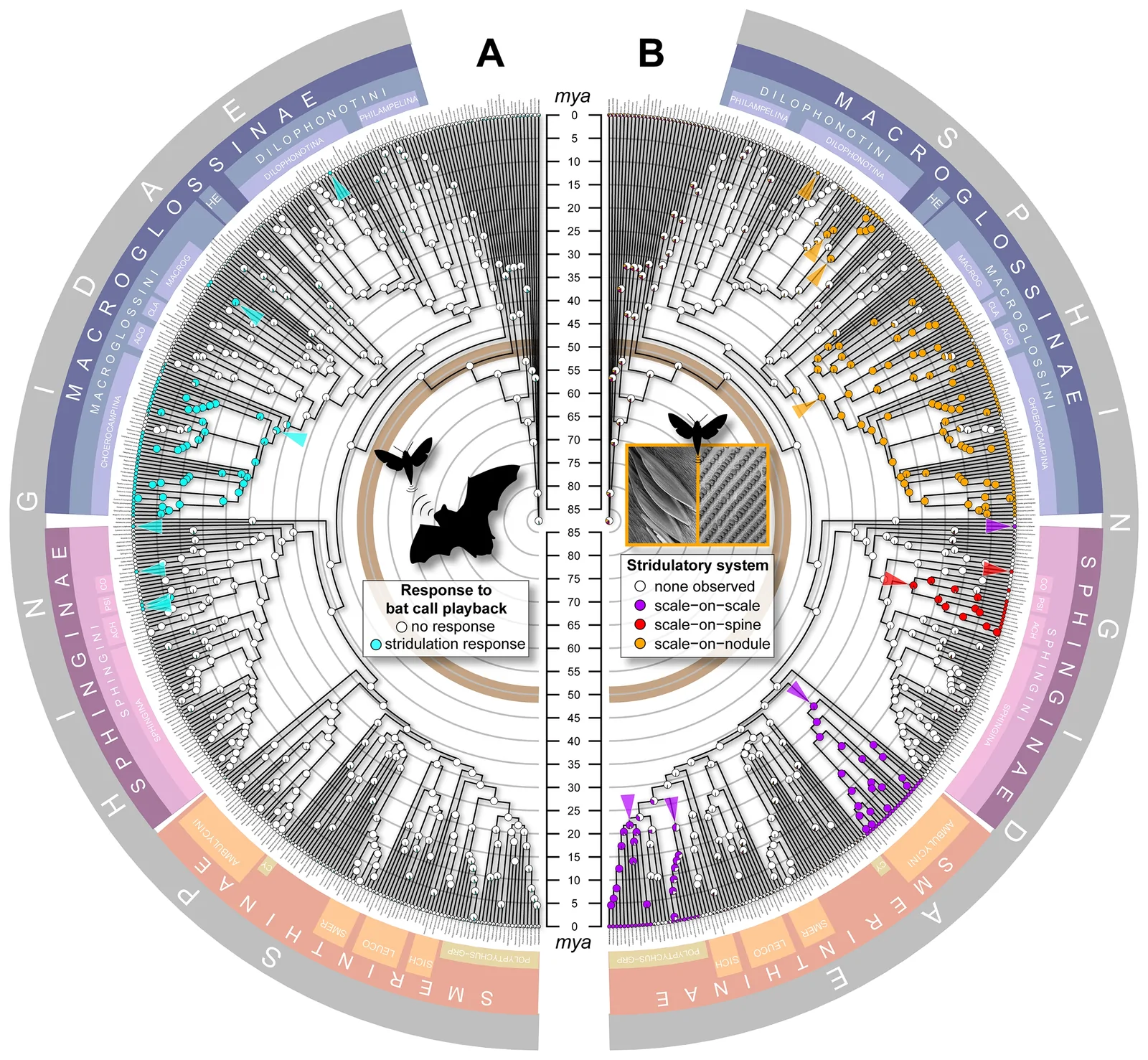
Evolution of bioacoustics across Sphingidae. (A) Multiple origins of anti-bat acoustic stridulation in male Sphingidae. Blue nodes indicate ASR of a stridulatory response to bat call playback. Blue arrows indicate nodes with the highest likelihood of independent origins of the response. (B) Evolution of male-based acoustic stridulatory systems in Sphingidae. Orange nodes denote a scale-on-nodule system, red a scale-on-spine system and purple a scale-on-scale system. Arrows denote inferred independent origins among the three systems. The brown arc at approximately 50 mya represents the approximate time that laryngeal echolocation in bats first evolved [43,55]. Abbreviated clade names: HE = Hemarini, MACROG = Macroglossina, CLA = Clarinina, ACO = Acosmerygina, CO = Cocytiina, PSI = *Psilogramma* genus-group, ACH = Acherontiina, CY = *Cypa* genus-group, SMER = Smerinthini, LEUCO = Leucophlebiini, SICH = Sichiini.

### (b) Evolution and description of stridulatory systems in male Sphingidae

After identifying the taxa that emit sound in response to bat echolocation and/or touch, stridulatory structures were examined using SEM, micro-CT and high-speed videography originally captured by [32] (supplementary material, table S4). In male hawkmoths, the scraper apparatus typically comprises a row or patch of enlarged scales located on the dorsal or dorsolateral surface of the valvae, which interact with one of three types of file structures—scales, spines or nodules—positioned on the ventral surface or posterior margin of the T8. Both direction and temporal aspects of valvular movement relative to T8 differ markedly among subfamilies (see details on these behaviours in the supporting information text section of the appendix in supplementary material and movies S1–S3 of [32]), demonstrating variation in sound production mechanisms beyond their structural components.

Our study confirmed three distinct stridulatory systems in Sphingidae: (i) a scale-on-scale system (primarily observed in Smerinthinae), (ii) a scale-on-spine system (restricted to certain Sphinginae), and (iii) a scale-on-nodule system (prevalent among Macroglossinae) (figures 1B and 2; supplementary material, table S5). Although valvular scales in Macroglossinae have long been suspected to play a role in stridulation [32,39], the corresponding surface with which they interact had not been characterized. Our study revealed that the file component is not composed of opposing scales or spines but instead consists of intricate microsculpturing (i.e. nodules) on the ventral surface of T8. Dissections and micro-CT imaging revealed that valvular scraper scales in many Macroglossinae are positioned so that they contact the nodule-studded region during valve contraction and relaxation. Notably, the structure and complexity of these nodules vary substantially, even among closely related species.

**Figure 2.**
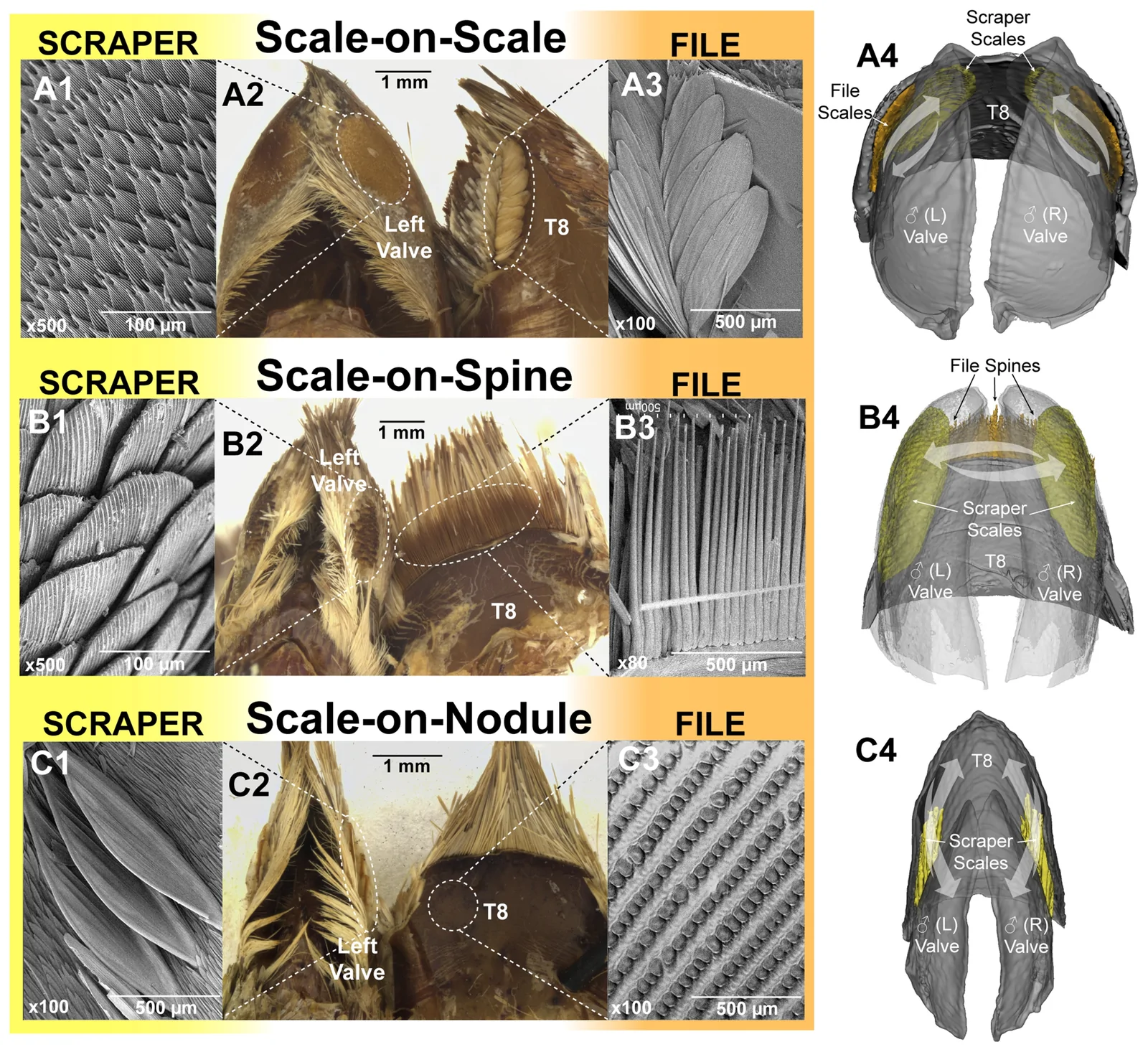
Male-based stridulatory systems in Sphingidae. The scale-on-scale system is displayed across the top (Smerinthinae: Ambulycini: *Ambulyx moorei* A1–A4), the scale-on-spine system across the middle (Sphinginae: Sphingini: *Psilogramma menephron* B1–B4) and the scale-on-nodule system across the bottom (Macroglossinae: Macroglossini: *Daphnis hypothous* = C1 and C3, *Xylophanes pluto* = C2, *Xylophanes falco* = C4). Dissections of these systems are displayed in A2, B2 and C2. In each, the dorsal surfaces of the male valvae are positioned to the left and the ventral surface of the eighth abdominal tergite (T8) is exposed on the right. SEMs of scraper scales are highlighted in A1, B1 and C1 and SEMs of their corresponding file structures are shown in A3, B3 and C3. Micro-CT reconstructions of the male-based stridulatory systems in Sphingidae are displayed in A4, B4 and C4. Grey arrows indicate the directions that each genital valve and its scraper scale patch (yellow) move against the opposing file structure (orange) on T8. (A4) Ventrocaudal view of *A. moorei*: the left and right valvae move in opposition towards and away from each other as the scraper scale patch is pressed and rubbed across corresponding patches of file scales on the ventral surface of T8. (B4) Ventrocaudal view of *P. menephron*: the left and right valvae move in tandem laterally while pressing the scraper scales against a row of stiff file spines that protrude from the posterior margin of T8. (C4) Ventrocaudal view of *X. falco*: the left and right valvae, during contraction, move dorsally underneath T8 causing the scraper scales to rub against fine, nodule-studded microsculpturing on the ventral surface of T8. See supplementary material, videos S1–S3, for three-dimensional renderings of these systems.

To reconstruct the evolution of stridulatory system morphology, we performed an ASR using stridulatory system type as a character (supplementary material, tables S5 and S6) and mapped the three distinct mechanisms onto the phylogeny (figure 1B). The scale-on-nodule system was only observed in Macroglossinae and appears to have evolved at least twice independently (figure 1B, orange arrows): once near the base of the Macroglossini (including the Choerocampina, Acosmerygina, Clarinina and Macroglossina) and again (perhaps numerous times) among the Dilophonotini (i.e. *Callionima*). This mechanism was subsequently lost in several lineages of Macroglossini that exhibit shifts towards crepuscularity (e.g. *Hyles*) and diurnality (e.g. *Hemaris* and *Macroglossum*). These specific taxa lack scraper scales on the valvae and the size and complexity of the microsculpturing on T8 is greatly reduced. The scale-on-spine system is unique to the Sphinginae and was observed in Acherontiina, Cocytiina and members of the *Psilogramma* genus group (figure 1B, red arrows). While *Agrius* (Acherontiina) possesses modified valvular scales that abut a row of spines on the apex of T8, our behavioural experiments failed to elicit stridulatory responses from any *Agrius* individual tested. *Panogena* (*Psilogramma* genus group), whose behavioural response remains to be tested, also displays the scale-on-spine mechanism. While not present in the dated tree, *Notonagemia analis* produces ultrasound to bat call playback and has a scale-on-spine arrangement similar to other members of the *Psilogramma* genus group. Lastly, the scale-on-scale system has evolved independently among two divergent clades of Smerinthinae (the Ambulycini and members of the *Polyptychus* genus group) (figure 1B, purple arrows). A strikingly similar arrangement of scraper scales abutting file scales is also apparent in *Meganoton nyctiphanes* (Sphinginae). This species is the only member of the Sphinginae in which we found a scale-on-scale system.

Our ASR analysis of hawkmoth stridulatory systems (figure 1B) shows different evolutionary patterns when compared to those reconstructed for behavioural responses to bat calls (figure 1A). Some Sphinginae (e.g. Acherontiina and species of the *Psilogramma* genus group), which did not respond to bat calls or touch, have robust stridulatory organs. Furthermore, several lineages within the Smerinthinae and early-diverging Macroglossinae (e.g. Dilophonotini) that supposedly lack hearing organs exhibit well-developed scraper scales and corresponding modifications to file structures. While stridulatory responses to tactile stimulation were observed in the earless (deaf) Ambulycini (Smerinthinae) [32], such responses were not detected in members of the *Polyptychus* genus group. However, dissections revealed that most *Polyptychus* and *Pseudoclanis* species possess scale-on-scale stridulatory systems. While possessing the ability to hear echolocating bats plays an important role in predator evasion in some insects such as tiger moths [22] and tiger beetles [56], it may not have been a universal requirement for the evolution of stridulatory structures across all hawkmoths. Beyond its defence role, sound production may also function in sexual communication [29], potentially representing one of the rare instances in which a putative ancestral defensive system was co-opted for courtship function rather than the reverse [57,58]. Some moths can modulate the intensity, frequency or pattern of their acoustic signals to facilitate quiet, close-range intraspecific interactions while minimizing detection by rivals or predators [59]. While surprisingly little is known regarding the use of bioacoustics for courtship in Sphingidae, male *Psilogramma* (Sphinginae: Sphingini) have been observed stridulating in the presence of females [60].

### (c) Acoustics of hawkmoth stridulation

After measuring stridulatory bouts from all ultrasound producing species (supplementary material, tables S7 and S8), the acoustic data were clustered using UMAP to visualize patterns in two-dimensional space (figure 3A,B). Resulting clusters largely corresponded to stridulatory system type and the presence or absence of hearing organs. Specifically, acoustic signals from eared Sphinginae possessing scale-on-spine mechanisms were tightly clustered and distinct from those of the other systems (figure 3B, red points). Contrary to the tight clustering exhibited by the acoustics of the scale-on-spine system, the scale-on-nodule system was broadly distributed across acoustic space (figure 3B, orange points). Signals from deaf Smerinthinae with scale-on-scale mechanisms formed a dispersed cluster that overlapped with the ultrasound emissions of some scale-on-nodule Macroglossinae (figure 3B, purple points).

**Figure 3.**
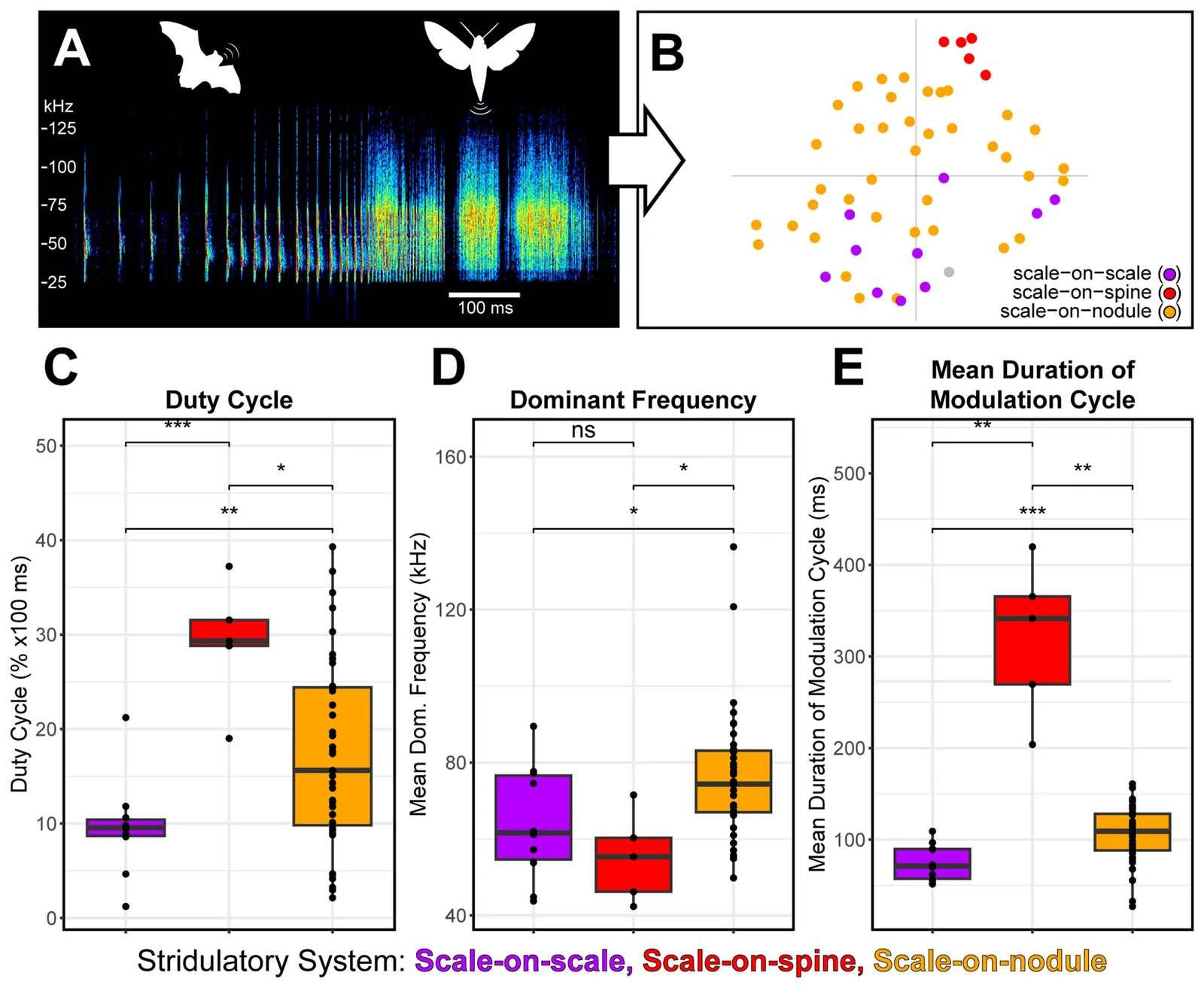
Acoustic analysis of hawkmoth anti-bat sounds. (A) Spectrogram of an anti-bat acoustic response from *Xylophanes ceratomioides* (Macroglossinae) to bat call playback. (B) UMAP clustering of moth acoustic response data. Colours correspond with stridulatory system. The grey dot indicates the unknown mechanistic status of *Cocytius lucifer* (Sphinginae) which does produce ultrasound but lacks any apparent genital-based stridulatory structures. Acoustic characteristics based on stridulatory system are displayed as box plots: (C) duty cycle of a 100 ms window of sound (%), (D) mean dominant frequency (kHz) and (E) mean duration of modulation cycle (ms). In (C–E), *t*-test significance levels are annotated based on conventional *p*-value thresholds (*0.01 < *p* ≤ 0.05, **0.001 < *p* ≤ 0.01, ***0.0001 < *p* ≤ 0.001; ns, *p* > 0.05).

We compared key acoustic features of hawkmoth ultrasound across different stridulatory systems to assess whether morphological variation correlates with differences in sound output (figure 3C–E; supplementary material, table S7). Percent duty cycle—the proportion of time during which sound is actively emitted—was significantly higher in moths with scale-on-spine and scale-on-nodule systems compared to those with the scale-on-scale system (figure 3C). Behavioural studies have shown that ultrasound with a duty cycle of approximately 20% can interfere with bat echolocation and that the effectiveness at disrupting predator detection increases progressively with higher duty cycles rather than reflecting a strict threshold [32]. In our dataset, eared Sphinginae and some eared Macroglossinae exhibited duty cycles high along this gradient, whereas deaf taxa, such as most Smerinthinae, had consistently lower values. This suggests that the ability to detect bat calls may be closely linked to the production of anti-bat ultrasound with jamming potential.

We also examined mean dominant frequency—the frequency at which maximum acoustic energy is concentrated. While there was no significant difference between scale-on-scale and scale-on-spine systems, species with the scale-on-nodule mechanism differed significantly from both (figure 3D). Additionally, mean duration of modulation cycle—the length of a single stridulatory bout—varied significantly across the three systems (figure 3E). Sphinginae, which use a scale-on-spine mechanism, exhibited the longest emission periods (approx. 350 ms). We postulate that the increased duration of noise production may better enable these moths to confuse echolocating bats.

Despite being on the low end of the jamming-potential spectrum, many deaf moths emit sound in response to touch, potentially serving a deimatic function that startles predators, including bats, which scoop and manipulate insect prey with their wing and tail membranes [61,62]. During these encounters, hawkmoths may have sufficient time to stridulate and avoid capture. Alternatively, stridulation may function as a form of acoustic mimicry [32,35]. Unlike chemically defended tiger moths that use ultrasound as an aposematic signal [22], hawkmoths are palatable to bats [32]. Thus, some Sphingidae may exploit mimicry of noxious models to facilitate escape [36].

One significant pattern that was uncovered through this study is that the acoustic features of Macroglossinae are much less consistent than others. Among the three stridulatory systems investigated, the macroglossine-specific scale-on-nodule system tends to show the greatest variability with respect to acoustic parameter space (figure 3B). Given that these differences are highly variable between even closely related macroglossine species, they may serve both as an anti-bat strategy and as a species discrimination signal in mating contexts. Macroglossinae are also broadly distributed geographically [63], and it is possible that there is a latitudinal and bat abundance-based reason for why some taxa produce ultrasound capable of jamming bats and others do not, but this remains to be explored.

### (d) Morphological variation in stridulatory systems

Given the broad acoustic variation observed across these systems, we examined their elements in greater detail with light microscopy and SEM (figure 4; see supplementary material, figures S167–S283, in the appendix for additional SEMs of MGCL specimens). Using morphological data derived from scraper scales (supplementary material, table S9), we conducted a discriminant analysis classification to test whether subfamilies, and by extension, stridulatory systems, form distinct clusters in morphospace (supplementary material, table S11; figure 4A). The analysis revealed that Macroglossinae, Smerinthinae and Sphinginae exhibit distinct distributions and varying degrees of morphological variance in multivariate space, with each generally clustering separately based on scraper scale aspect ratio and their perimeter. Notably, this approach also provided reasonable discriminatory power at finer taxonomic levels, successfully differentiating several subtribes within Macroglossinae (supplementary material, table S12; figure 4B). While Macroglossinae present the largest scraper scales on average among the three subfamilies tested, they also exhibited the greatest amount of scale morphological diversity, possibly indicating either relaxed structural constraint or the evolution of multiple adaptive strategies for sound production within the scale-on-nodule system. These findings suggest that scraper morphology contains phylogenetically and functionally informative signals relevant to stridulatory system evolution. A PGLS regression was used to compare the size of scraper scales with the diameters of nodules found on T8 while accounting for shared evolutionary history among species (supplementary material, table S13; figure 4C). We found that the genera that exhibit the largest (per proxy of perimeter length) scraper scales also have the largest nodules.

**Figure 4.**
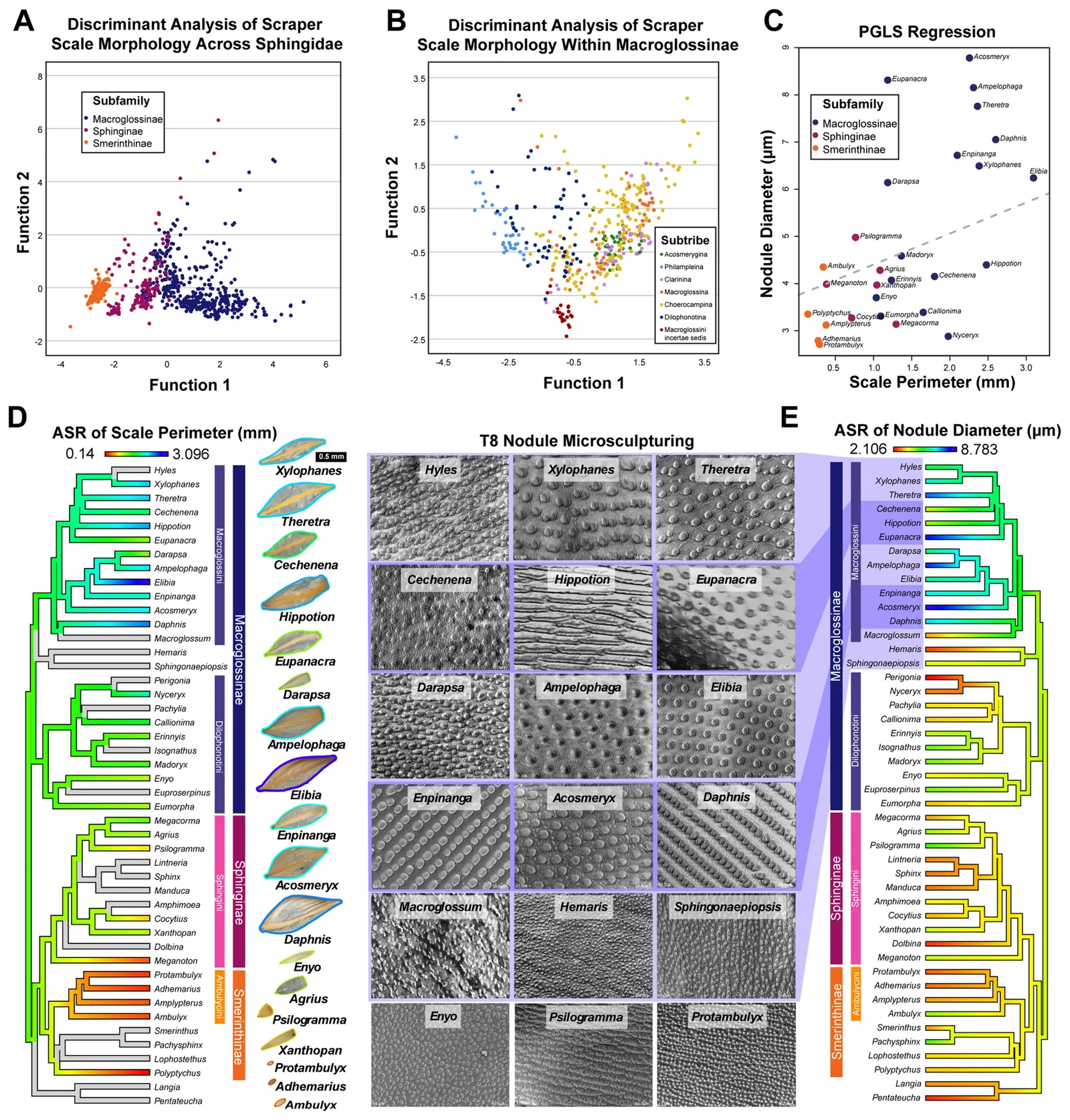
Morphological diversity of stridulatory systems. (A) Discriminant analysis plot of Sphingidae based on scraper scale morphology. Each point represents a scraper scale measured from a dissected specimen and is coloured based on their respective subfamily. (B) Discriminant analysis plot based on scraper scale morphology showing subtribal clustering within Macroglossinae alone. (C) Phylogenetic generalized least squares (PGLS) regression plot of scraper scale perimeter in comparison with microsculpturing nodule diameter. Macroglossinae possessing the unique scale-on-nodule stridulatory system are predominantly recovered in both the upper and lower right quadrants of this plot. Hawkmoths that employ scale-on-scale (Smerinthinae = orange) and scale-on-spine (Sphinginae = pink) stridulatory systems cluster in the lower left quadrant of the graph, reflecting their relatively smaller scraper scales and nodules. (D) Continuous trait mapping of the mean perimeter of valvular scraper scales. Example scraper scales (all to scale) are shown. (E) Continuous trait mapping of the mean diameter of nodules on the ventral surface of T8 (the file of Macroglossinae). SEMs (centre) showcase the unique nodule microsculpturing found across Sphingidae. All SEMs are displayed at 1000× magnification.

While scraper scale morphology diverges significantly across the three systems (e.g. scraper scales tend to be the largest among nocturnal Macroglossini and smallest among ultrasound producing Smerinthinae), the scale-on-nodule system alone exhibits substantial morphological diversity, with both the size and shape of scraper scales and their corresponding nodule-based file components varying substantially across genera. We measured the mean perimeters of the valvular scraper scales and mean nodule diameter (a proxy for nodule size) on the ventral surface of T8 and mapped these traits onto a pruned, generic-level version of the hawkmoth phylogeny (supplementary material, table S13; figure 4D,E). Within Macroglossini, scraper scales are among the largest observed (e.g. *Elibia*, *Daphnis*, *Theretra*), and nodule microsculpturing is especially elaborate, exhibiting increased size (e.g. *Acosmeryx*), wider spacing (e.g. *Elibia*), and, in some taxa, organization into parallel rows (e.g. *Enpinanga* and *Daphnis*). *Hippotion* offers an extreme example where the nodules are merged into narrow, parallel ridges. Taxa that utilize non-nodule based systems of stridulation (Sphinginae and Smerinthinae) possess especially small scraper scales. While all hawkmoths that were dissected exhibited some form of microsculpturing on the undersurface of T8, in Macroglossinae that do not stridulate (e.g. *Hemaris* and *Macroglossum*), and among taxa that exhibit the scale-on-scale and scale-on-spine systems, the nodules comprising the microsculpturing are much smaller and more densely spaced.

## 4. Conclusion

In this study, we investigated the evolution of stridulatory systems and ultrasound production in hawkmoths through a combination of field experiments and collections-based research, with the goal to elucidate the defensive bioacoustic traits of this classic predator–prey arms race. Based on our analyses, anti-bat stridulatory behaviour in Sphingidae has potentially arisen independently up to seven times in divergent lineages and has manifested as distinct ultrasound-producing mechanisms. The three stridulatory systems examined here share a broadly similar architecture, with the scraper involving valvular scales and the file involving some sort of modification of the ventral or posterior surface of the last abdominal tergite. The repeated, independent emergence of such traits across divergent lineages underscores how persistent predation pressure can drive the evolution of novel yet similar strategies in distantly related groups, reinforcing the view that predator–prey conflict is one of the most powerful forces shaping evolutionary innovation.

## Supplementary Material



## Data Availability

All data and scripts are provided as supplementary material. Supplementary material is available online [64].
