# Supplementary material for: The repeated evolution of anti-bat bioacoustics in hawkmoths
Source: Proc Biol Sci. 2026 Nov 4;293(2082):20262303. doi: 10.1098/rspb.2026.2303 (PMC13730811; doi:10.1098/rspb.2026.2303)
Supplement: Supplementary file 1 [file rspb.2026.2303.zip › rspb.2026.2303/rspb20262303_si_001.pdf]

Appendix

The repeated evolution of anti-bat bioacoustics in hawkmoths

Proceedings B 10.1098/rspb

This PDF file includes:

Supporting Information Text:

- Descriptions of stridulatory systems in male Sphingidae
- Legends for Supplementary Tables S1 to S13
- Legends for Supplementary Movies S1 to S3
- Descriptions of Additional Supplementary Files

Supporting Information References

Figures S1 to S166: Habitus images of MGCL voucher specimens

Figures S167 to S283: SEMs of stridulatory systems

Other supporting materials (separate files) for this manuscript include the following:

- Tables S1 to S13
- Movies S1 to S3
- Supplementary tree files 1 and 2

SUPPORTING INFORMATION TEXT

Descriptions of stridulatory systems in male Sphingidae

To examine and describe the diversity of stridulatory structures in male hawkmoths, we dissected both known and putative ultrasound producing hawkmoths. Stridulatory structures were studied using SEM and micro-CT, as well as highspeed recordings of the stridulatory valvae in motion. Three types of hawkmoth stridulatory systems were found: scale-on-scale system (primarily observed in the Smerinthinae), scale-on-spine system (exclusively seen among some Sphinginae), and scale-on-nodule system (found in many Macroglossinae) (Figures 1B & 2, SEMs of stridulatory systems). The scraper apparatus in male hawkmoths comprises a row or patch of specialized scales located on the dorsal or dorsolateral surface of the valvae, which interact with one of three distinct file types situated on the ventral surface or posterior margin of the eighth abdominal tergite. The kinematics of valval movement differ substantially among subfamilies (see Movies S1–S3 of Kawahara & Barber [1]), resulting in variation in how these structures engage during sound production. A detailed description of these stridulatory systems is provided below.

1.) Scale-on-scale system:

Stridulation is produced when modified scales on the male genital valvae, hereafter referred to as scraper scales, are rubbed against opposing rows of specialized file scales located on the ventral surface of the eighth abdominal tergite. During sound production, the right and left valvae move in short, coordinated bursts upward and medially, bringing the scraper scales into contact with the ventral surface of the eighth tergite. Scraper scales vary in shape among taxa and are generally oriented parallel to the cuticular surface of the valvae. In stridulating members of Ambulycini, individual scraper scales are relatively flat and typically bilobed, bearing two distinct apices, and overlap to form dense, ovate patches on the dorsal surface of the valvae. By contrast, scraper scales in members like the *Polyptychus* genus group are strongly keeled along their longitudinal axis and terminate in a single pointed apex. These distinctive scales are tightly appressed to the valvular cuticle and form more diffuse patches compared to those observed in Ambulycini. In the smerinthine scale-on-scale system, scraper scales are relatively small, measuring approximately 0.06–0.66 mm in perimeter length, and are markedly reduced in size compared to the valvular scraper scales of the other two stridulatory systems.

Both groups possess two rows of enlarged scales arising from the posterior margin of the eighth abdominal tergite. Unlike surrounding scales, which project posteriorly away from the body, these specialized scales curve anteriorly across the ventral surface of the eighth tergite and function as the file, or *pars stridens*, in which the much smaller valvular scales are rubbed. In Ambulycini, file scales are typically elongate, appearing oblong (e.g., *Ambulyx moorei*), lanceolate (e.g., *Ambulyx pryeri*), or sickle-shaped (e.g., *Protambulyx* spp.) and are generally more than twice as long as they are wide. In contrast, file scales in taxa belonging to the *Polyptychus* genus group are shorter and more quadrate in shape. Ancestral state reconstruction recovered two putative independent origins of stridulation reliant involving this scale-on-scale configuration: once in the MRCA of the Ambulycini and again in the MRCA of the *Polyptychus* genus group. This inference is supported by the pronounced morphological differences in both scraper and file scales that are observed between the two clades.

In this system, the right and left valvae move rapidly in opposition to one another, producing two discrete sound emissions during each contraction-relaxation cycle. During the contraction phase, both valvae move medially toward one another and upward against the ventral surface of the eighth tergite (T8), bringing the scraper scales into contact with the file scales and generating sound. Following a brief period of silence, the valvae return to their resting positions. During the relaxation phase, as the valvae move laterally apart, a second sound pulse is produced. Species possessing this stridulatory system do not respond to bat ultrasound playback, but reliably stridulate in response to tactile stimulation. Across our recordings, the duration of sound emission

during the first half of the modulation cycle (contraction phase) was typically slightly longer than that of the second half (relaxation phase) (see D_1h_mean versus D_2h_mean in Tables S7–S8). The total duration of a single modulation cycle, averaged across all scale-on-scale stridulators, was significantly shorter than that observed in other stridulatory systems (~74.8 ms, SD = 16.0). For these taxa, the mean duration of the first half of the cycle was 35.1 ms (SD = 8.3), whereas the second half averaged 26.9 ms (SD = 7.0). Consistent with these short emission durations, mean percent duty cycles measured from 100-ms windows (100_ms_DC_mean) were also significantly lower than those of taxa possessing either of the other two systems, averaging only 9.6% across scale-on-scale stridulators.

2.) Scale-on-spine system:

In this system, stridulation occurs by rubbing patches of specialized scales on the male genital valvae (scraper scales), against spines located at the apex of the eighth abdominal tergite (file spines). Paired valvae extend well beyond the posterior margin of the eighth abdominal tergite and move laterally in unison, oscillating back and forth while pressing against a row of rigid spines that project posteriorly from the posterior margin. Scraper scales are positioned on the dorsal surface of the valvae and oppose specialized spines of the tergal margin. These scales are robust, flat, and rectangular, overlapping in a shingled arrangement to form large patches. The apical margins of the scraper scales may be serrated, as in *Megacorma obliqua*, or relatively smooth, as in *Psilogramma menephron*. The specialized file spines along the posterior margin of the eighth tergite show moderate variation in form across lineages: in *Psilogramma* species, they are stout, tapering bristles of relatively uniform length, whereas in taxa such as *Megacorna obliqua*, they vary more in length and exhibit spatulate apices.

Unlike the other two systems, the scale-on-spine system is characterized by synchronous movement of the paired genital valvae, which move together in the same direction while performing a repeated lateral back-and-forth motion. During this movement, valvae press dorsally against the row of file spines along the posterior margin of T8. Each lateral pass across the file produces sound, and prolonged bouts of stridulation consist of numerous such passes, interrupted by brief silent intervals as the valvae reverse direction. As discussed in the main text, scale-on-spine stridulators exhibit significantly longer mean modulation cycles and higher percent duty cycle than other systems. Across the five species measured, the mean total duration of a single modulation cycle was approximately 320 ms (SD = 66.6). The first and second halves of the cycle were similar in duration, with each pass across the file averaging approximately 152 ms (SD = 33).

3.) Scale-on-nodule system:

Unlike the previous two systems, in which the stridulatory apparatus consists of two opposing patches of modified scales or spines, this system involves only a single type of modified scale located on the dorsolateral surface of each genital valve. In stridulating species, both the right and left valvae bear patches of scraper scales that comprising approximately 10–30 individual scales, depending on the taxon. Rather than lying flat against each valve, these scraper scales are rotated slightly along their longitudinal axis and project at an oblique angle above the cuticle. During valvular contraction and relaxation, the scraper scales move across the ventral surface of the eighth abdominal tergite. The individual scales are seated in broad cuticular sockets and, in some cases, interlock with each other.

In Macroglossinae, the ventral surface of the eighth tergite lacks scales and instead is bears taxon-specific fine microsculpturing (file nodules). Although some degree of microsculpturing was observed across hawkmoth taxa examined, both the size (diameter) of nodules and complexity of organization were greatest in stridulating Macroglossinae. Within this subfamily, nodule size and arrangement vary substantially among species. Ancestral state reconstruction suggests that stridulation in male Macroglossinae evolved independently at least twice, once in the most recent

common ancestor (MRCA) of Macroglossini and again secondarily within the genus *Callionima* (Dilophonotini).

In this system, the right and left valvae move in opposition to one another in short, repetitive bursts. During the contraction phase, the valvae move medially toward each other while lifting dorsally and pressing against the ventral surface of T8. At this point, the enlarged scraper scales are thought to pass across the microsculptured cuticle of T8, producing sound. A brief period of silence follows as valvae reverse direction. During the relaxation phase, the valvae move laterally apart and return to their resting positions. As in the scale-on-scale system, sound emission during the first half of the modulation cycle (contraction phase) is typically longer than during the second half (relaxation phase). Across all scale-on-nodule stridulators, the mean duration of the first half of the modulation cycle was 51.5 ms (SD = 9.5), whereas the second half averaged 46.6 ms (SD = 8.5). The mean total duration of a single modulation cycle for this group was 106 ms (SD = 14.7).

Legends for Supplementary Tables* (separate file):*

Table S1: List of species tested for ultrasound production.

Table S2: Behavioral responses of moth species represented in the dated tree, indicating whether individuals produce stridulatory sounds in response to bat echolocation playback and/or tactile stimulation.

Table S3: Data matrix for the ASR of stridulatory response behavior.

Table S4: List of specimens dissected for examination of stridulatory structures.

Table S5: Type of stridulatory system exhibited by taxa in the dated tree.

Table S6: Data matrix for the ASR of stridulatory systems.

Table S7: Acoustic parameters measured from live hawkmoths during stridulatory behavior assays. A detailed description is provided in the accompanying Excel file README.

Table S8: Imputed acoustic data used for UMAP clustering and analysis.

Table S9: Measurements taken from valvular scraper scales.

Table S10: Measurements taken from microsculpturing on T8 (file component of the scale-on-nodule system).

Table S11: Summary statistics from discriminant analyses classifying scraper scale morphology at the subfamily level.

Table S12: Summary statistics from discriminant analyses classifying scraper scale morphology at the macroglossine subtribe level.

Table S13: Mean measurements for scale perimeter and T8 nodule diameter, at the generic level.

Legends for Supplementary Movies

Supplementary Movie S1 (separate file): µCT reconstruction of a scale-on-scale stridulatory system (*Ambulyx moorei*).

Supplementary Movie S2 (separate file): µCT reconstruction of a scale-on-spine stridulatory system (*Psilogramma menephron*).

Supplementary Movie S3 (separate file): µCT reconstruction of a scale-on-nodule stridulatory system (*Xylophanes falco*).

Descriptions of Additional Supplementary Files

Supplementary Tree File 1 (separate file): Dated tree of Sphingidae from Couch et al. [2] derived from a 537-locus anchored hybrid enrichment dataset.

Supplementary Tree File 2 (separate file): Genus-level phylogeny of Sphingidae built from Couch et al. [2].

SUPPORTING INFORMATION REFERENCES

1. Kawahara AY, Barber JR. 2015 Tempo and mode of antibat ultrasound production and sonar jamming in the diverse hawkmoth radiation. *Proceedings of the National Academy of Sciences* **112**, 6407–6412. (doi:10.1073/pnas.1416679112)

2. Couch CD, Masonick P, Plotkin D, Li X, Breinholt J, Rougerie R, Barber JR, Kitching **I**, Kawahara AY. 2026 The evolution of proboscis length and feeding behavior in hawkmoths (Lepidoptera: Sphingidae). *R. Soc. Open Sci.* **13**, 251697. (doi:10.1098/rsos.251697)

FIGURES S1–S166: Habitus images of MGCL voucher specimens (pages 6–33).

FIGURES S167–S283: SEMs of stridulatory systems (pages 34–67).

Habitus images of MGCL voucher specimens

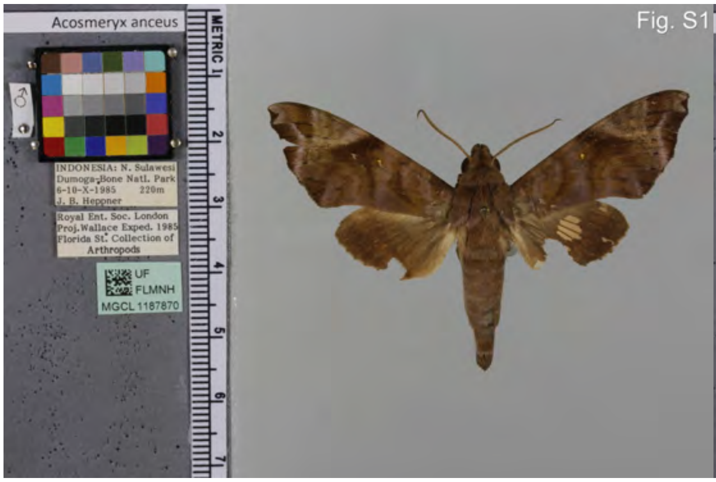

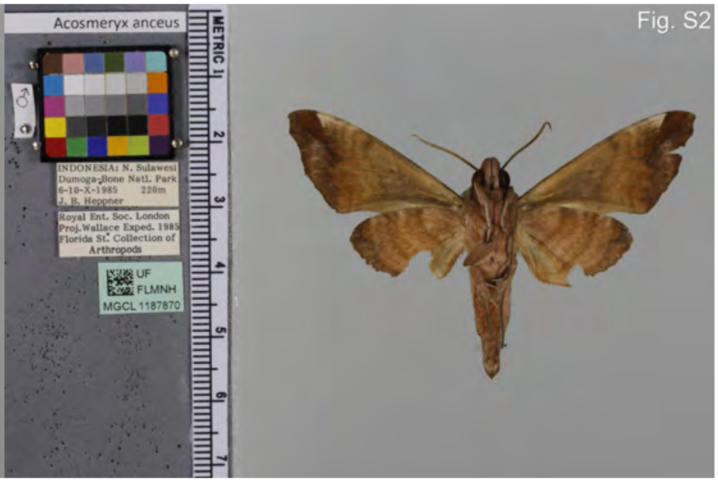

Acosmeryx_anceus_MGCL_1187870_D.JPG

Acosmeryx_anceus_MGCL_1187870_V.JPG

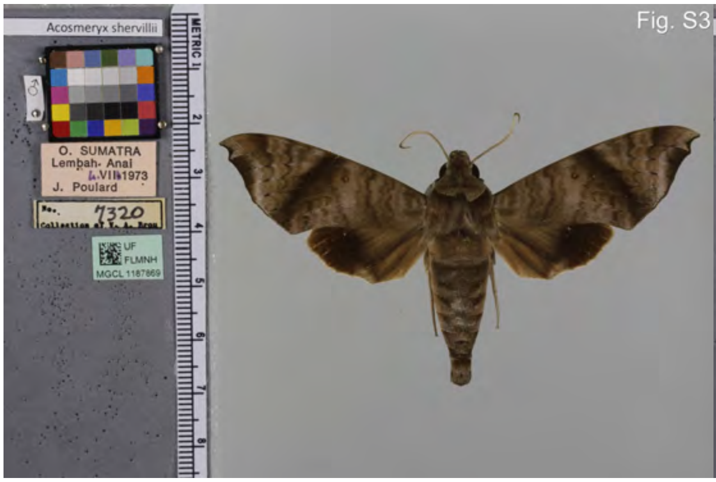

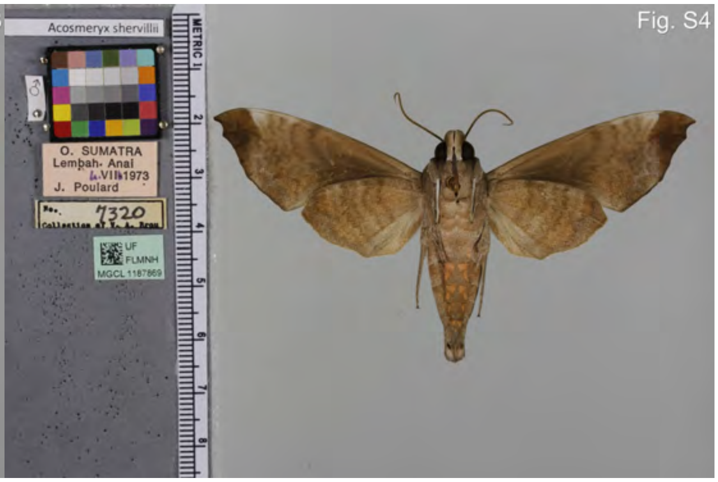

Acosmeryx_shervillii_MGCL_1187869_D.JPG

Acosmeryx_shervillii_MGCL_1187869_V.JPG

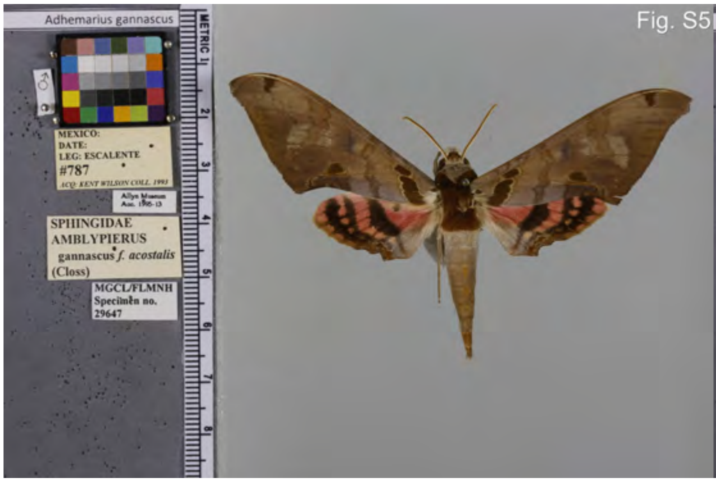

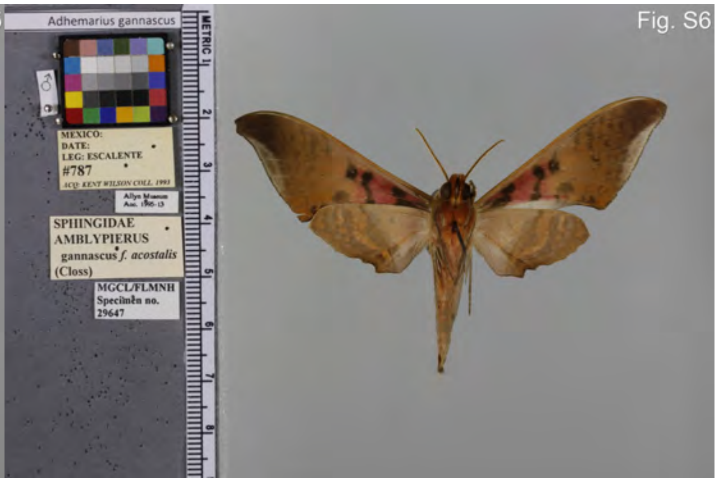

Adhemarius_gannascus_MGCL_29647_D.JPG

Adhemarius_gannascus_MGCL_29647_V.JPG

Habitus images of MGCL voucher specimens

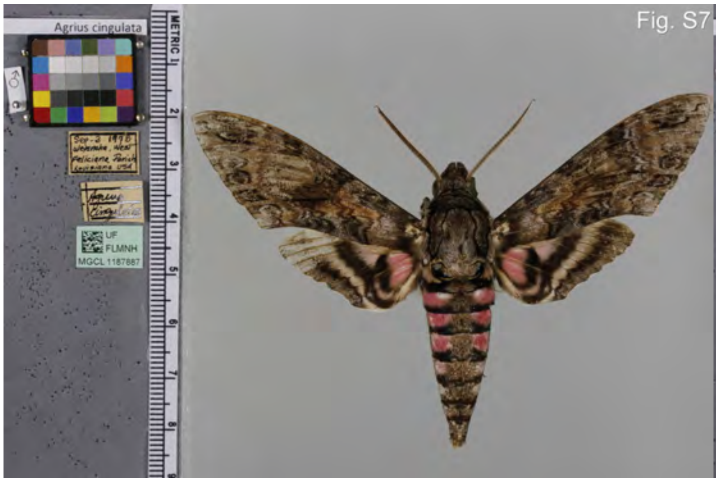

Agrius_cingulata_MGCL_1187887_D.JPG

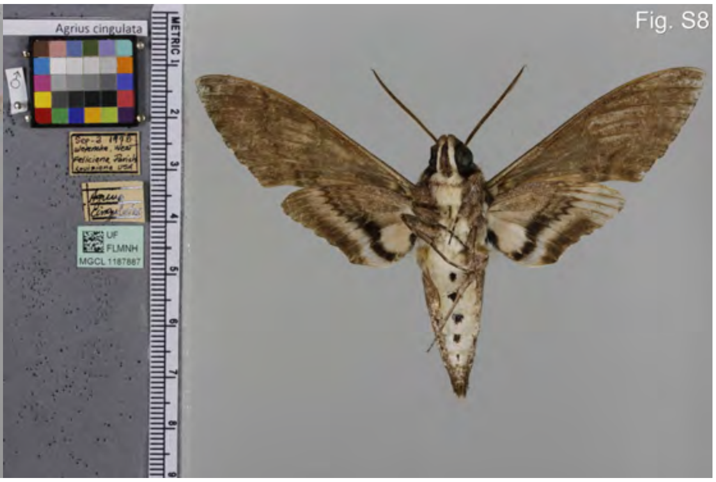

Agrius_cingulata_MGCL_1187887_V.JPG

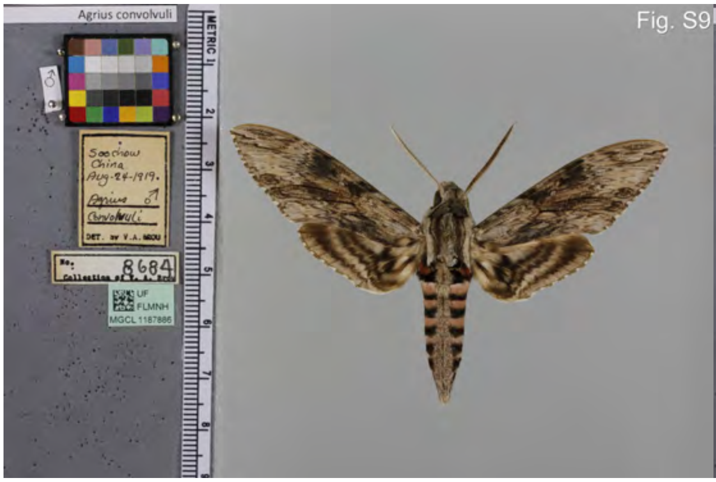

Agrius_convolvuli_MGCL_1187886_D.JPG

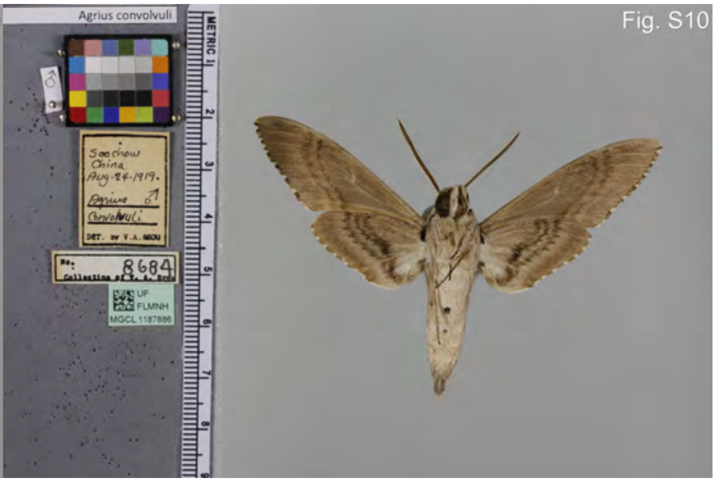

Agrius_convolvuli_MGCL_1187886_V.JPG

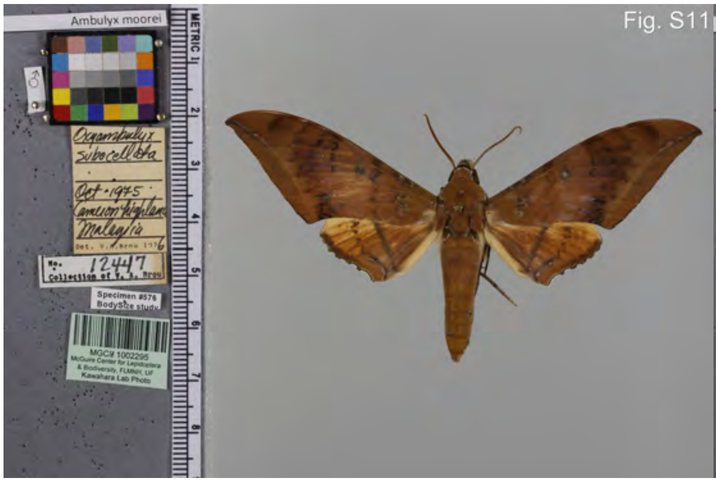

Ambulyx_moorei_MGCL_1002295_D.JPG

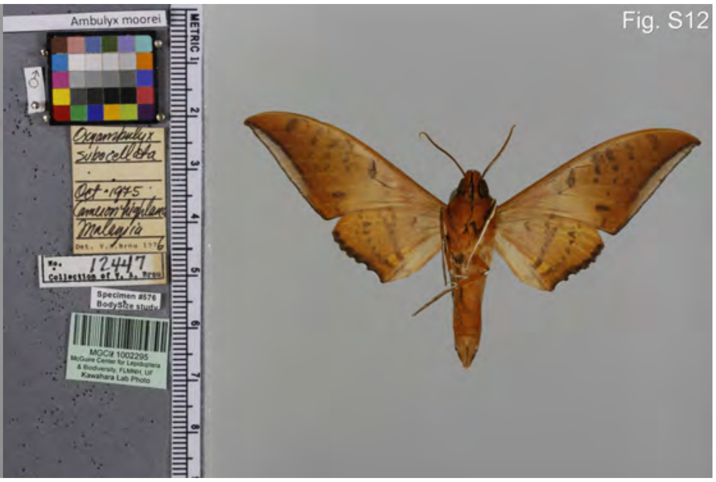

Ambulyx_moorei_MGCL_1002295_V.JPG

Habitus images of MGCL voucher specimens

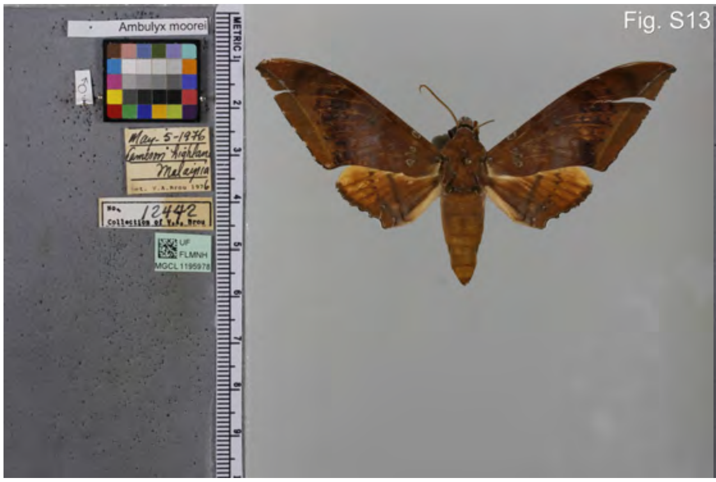

Ambulyx_moorei_MGCL_1195978_D.JPG

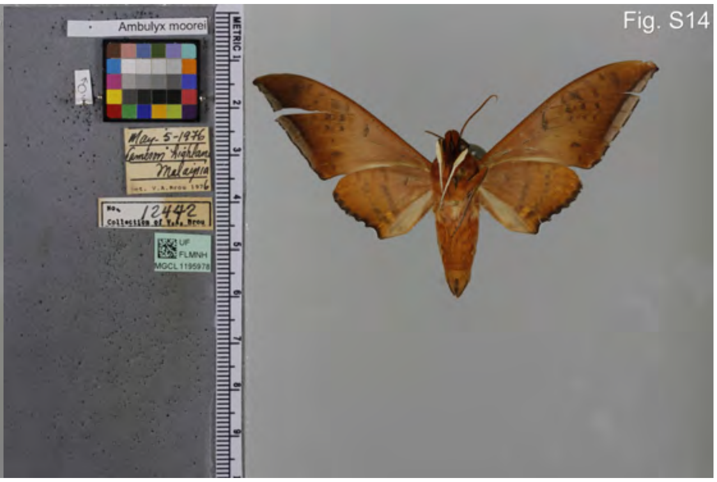

Ambulyx_moorei_MGCL_1195978_V.JPG

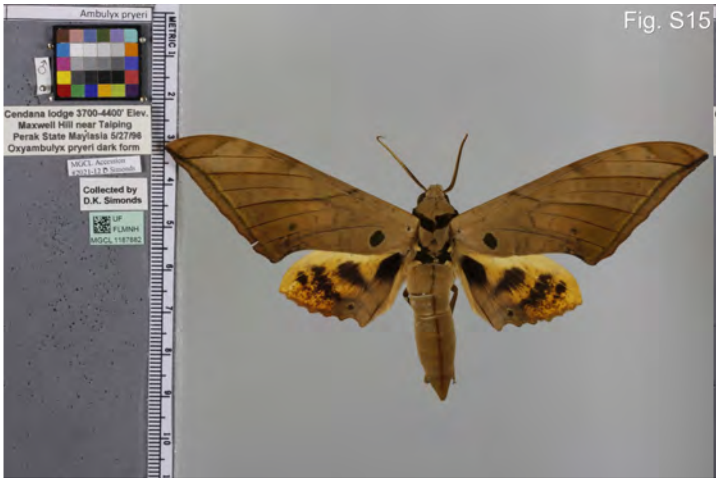

Ambulyx_pryeri_MGCL_1187882_D.JPG

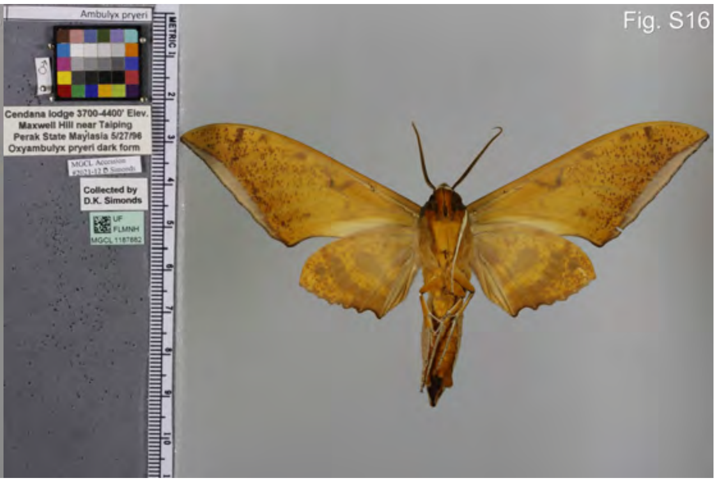

Ambulyx_pryeri_MGCL_1187882_V.JPG

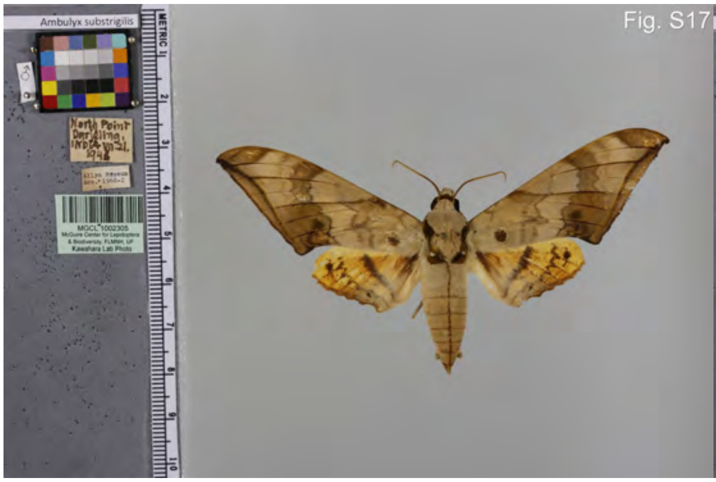

Ambulyx_substrigilis_MGCL_1002305_D.JPG

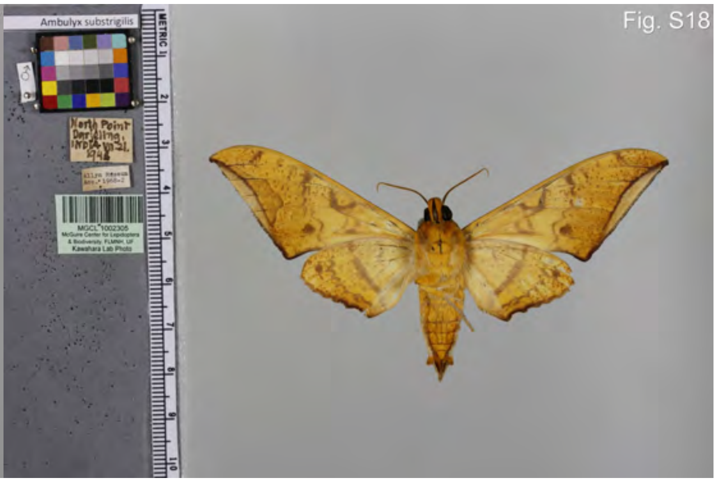

Ambulyx_substrigilis_MGCL_1002305_V.JPG

Habitus images of MGCL voucher specimens

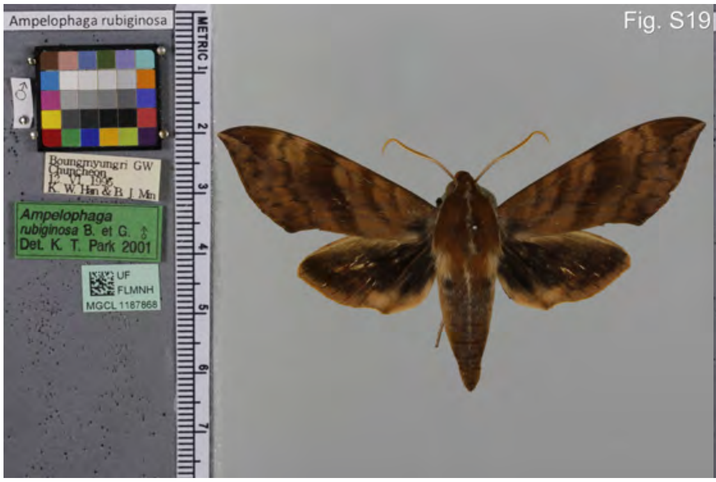

Ampelophaga_rubiginosa_MGCL_1187868_D.JPG

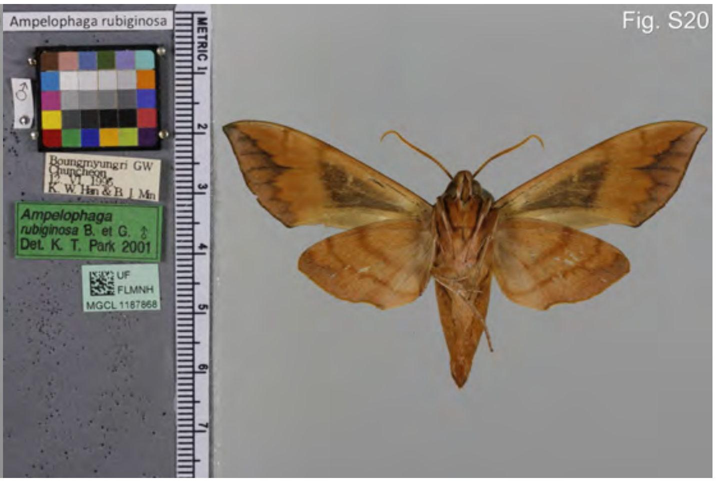

Ampelophaga_rubiginosa_MGCL_1187868_V.JPG

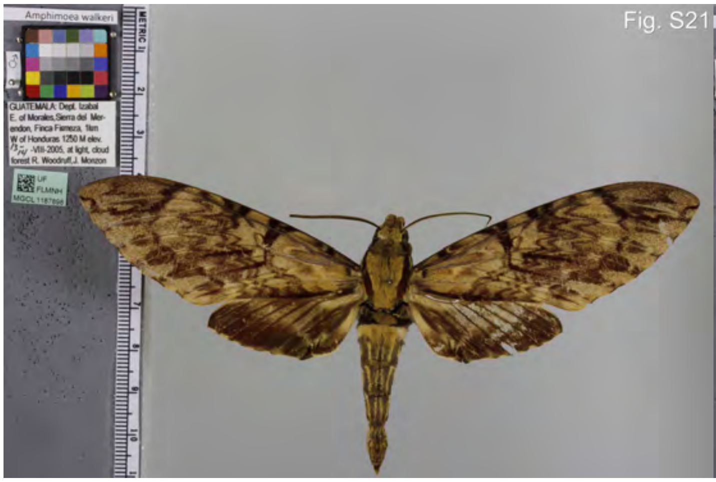

Amphimoea_walkeri_MGCL_1187898_D.JPG

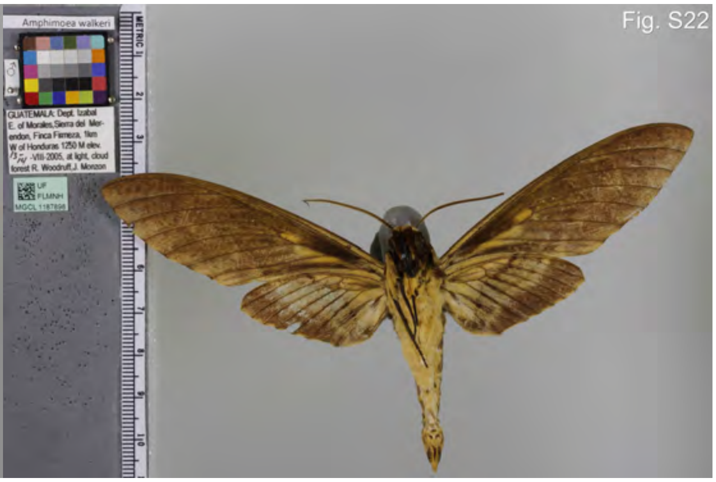

Amphimoea_walkeri_MGCL_1187898_V.JPG

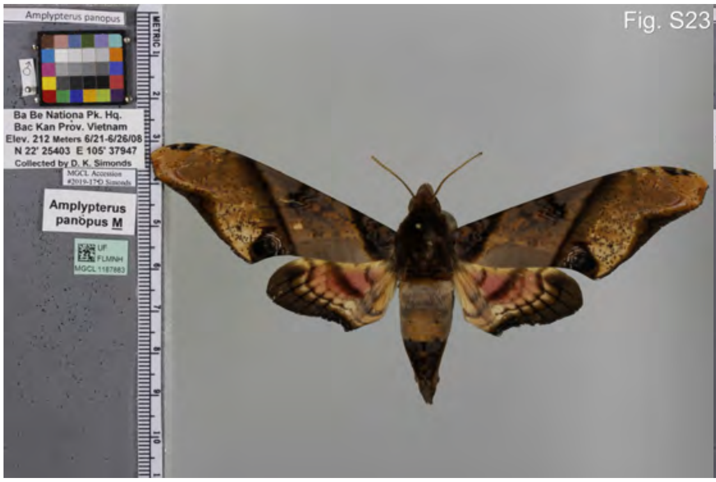

Amplypterus_panopus_MGCL_1187883_D.JPG

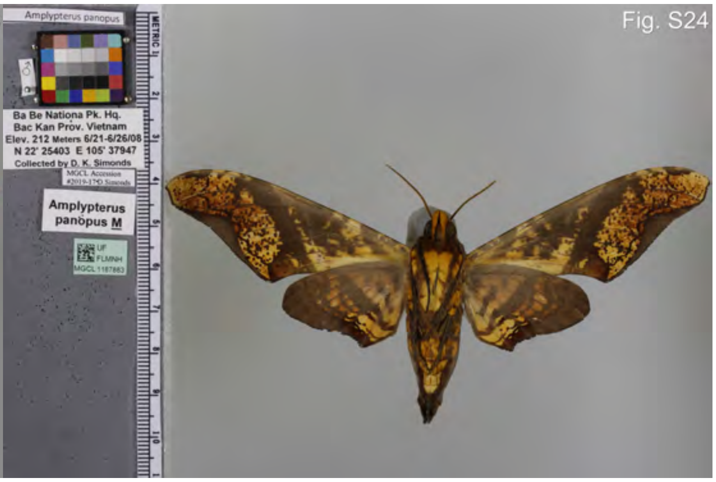

Amplypterus_panopus_MGCL_1187883_V.JPG

Habitus images of MGCL voucher specimens

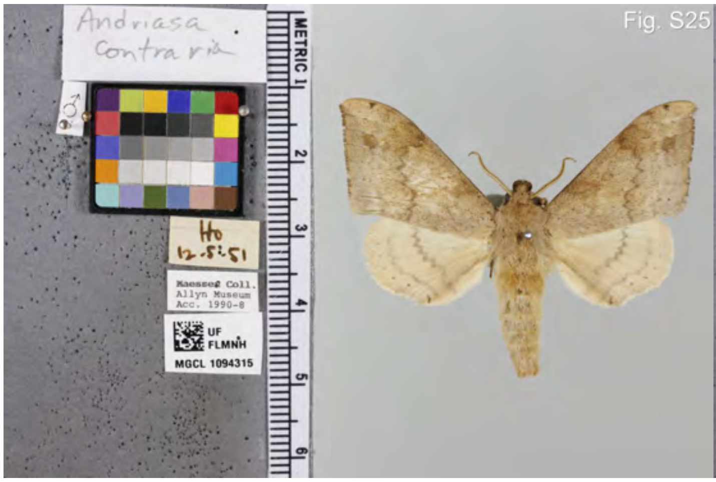

Andriasa_contraria_MGCL_1094315_D.JPG

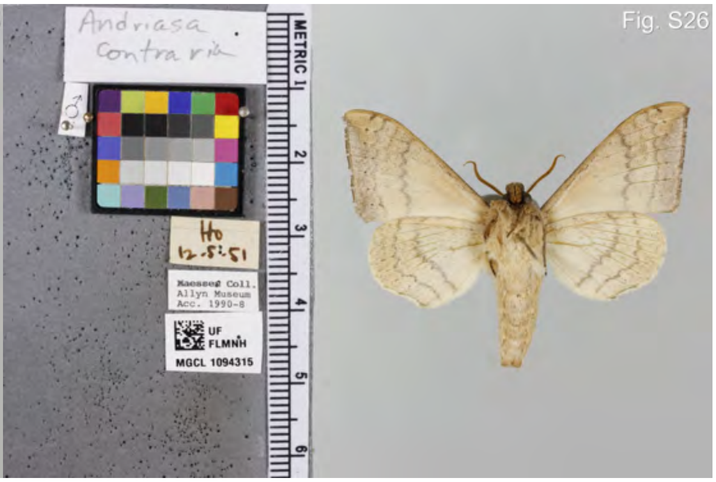

Andriasa_contraria_MGCL_1094315_V.JPG

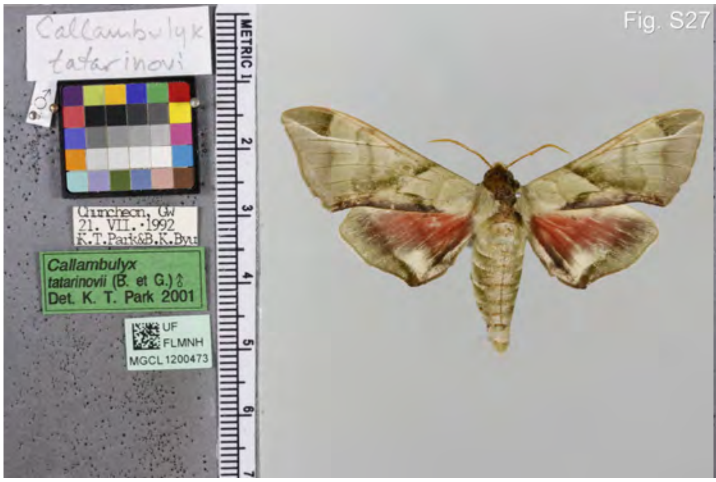

Callambulyx_tatarinovi_MGCL_1200473_D.JPG

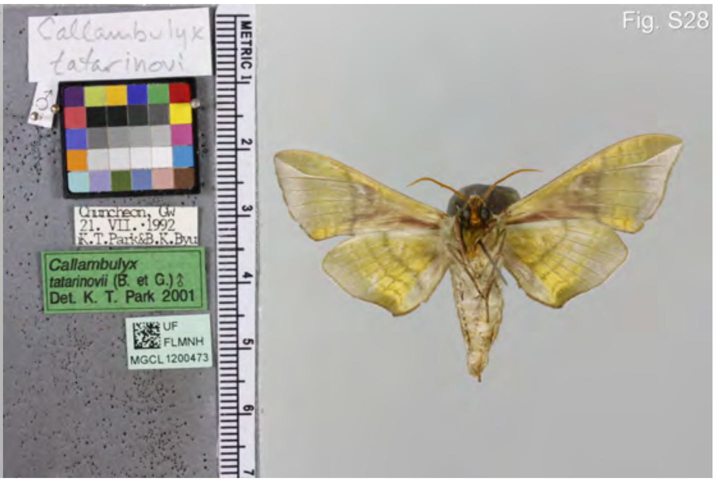

Callambulyx_tatarinovi_MGCL_1200473_V.JPG

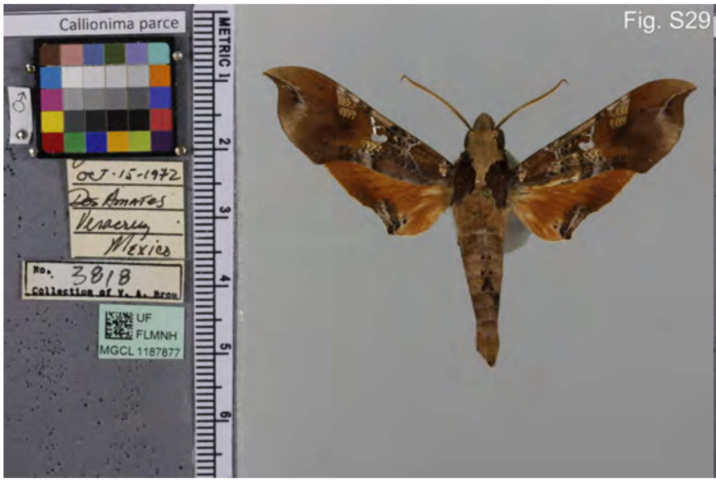

Callionima_parce_MGCL_1187877_D.JPG

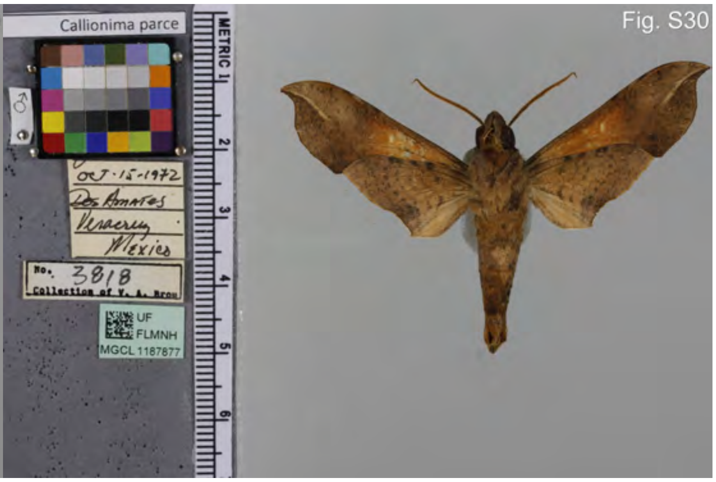

Callionima_parce_MGCL_1187877_V.JPG

Habitus images of MGCL voucher specimens

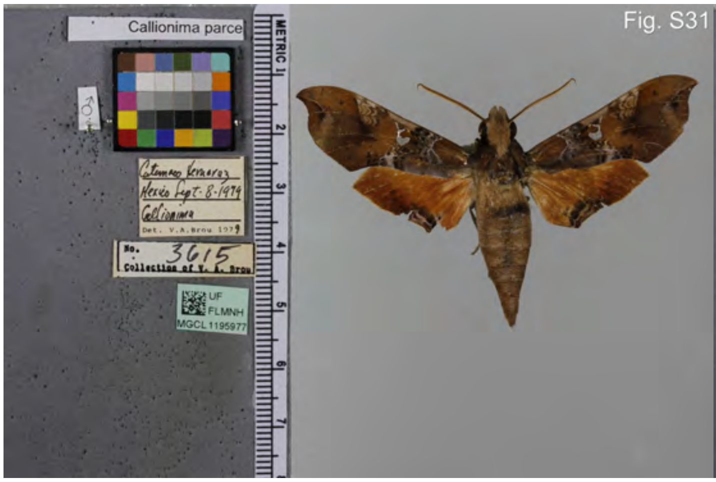

Callionima_parce_MGCL_1195977_D.JPG

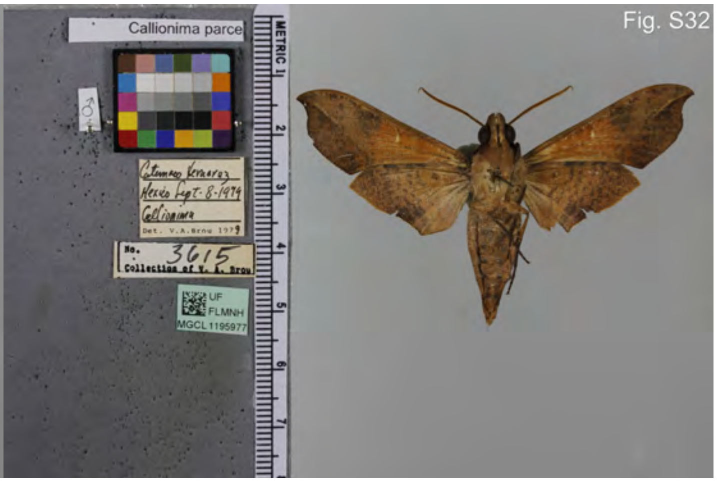

Callionima_parce_MGCL_1195977_V.JPG

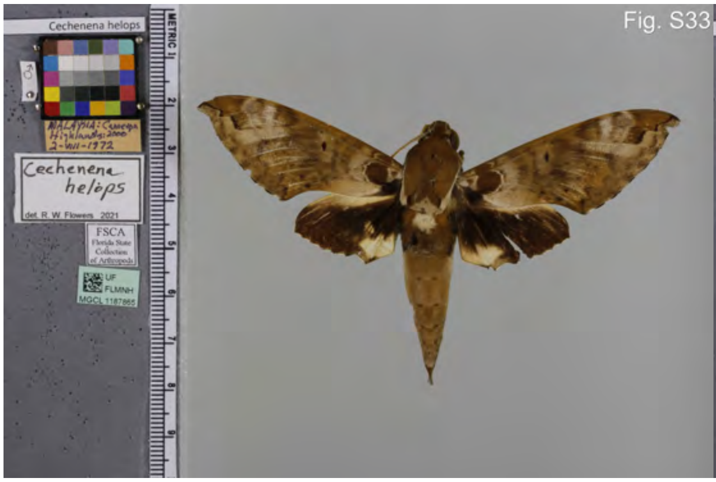

Cechenena_helops_MGCL_1187865_D.JPG

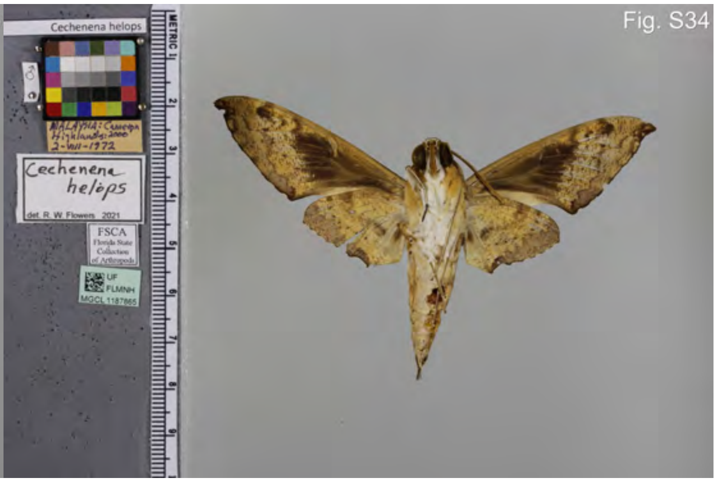

Cechenena_helops_MGCL_1187865_V.JPG

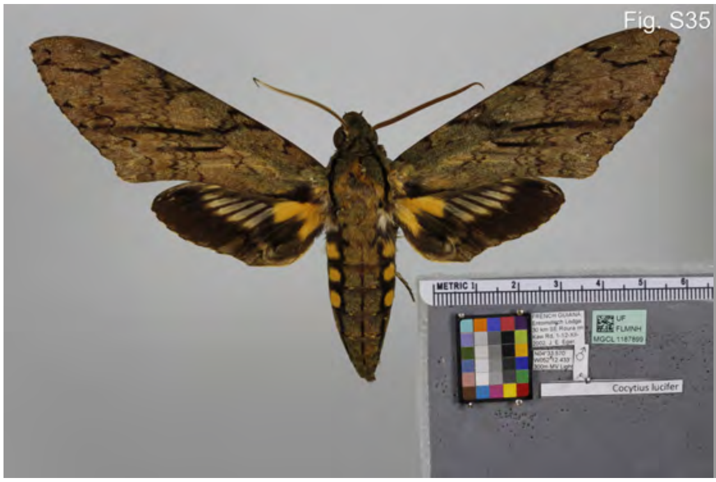

Cocytius_lucifer_MGCL_1187899_D.JPG

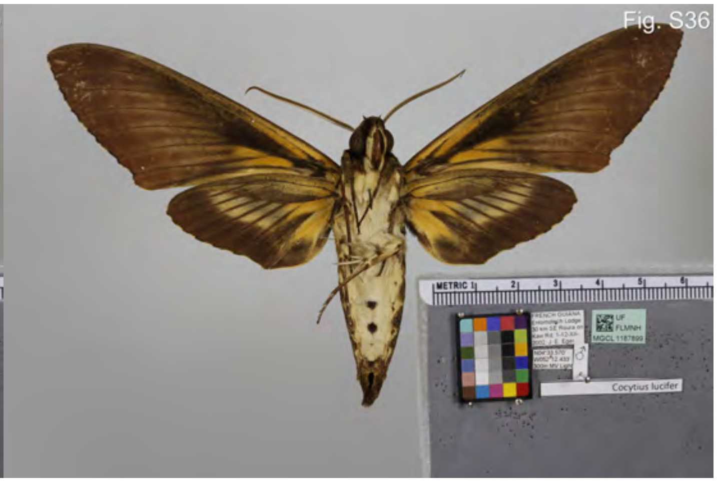

Cocytius_lucifer_MGCL_1187899_V.JPG

Habitus images of MGCL voucher specimens

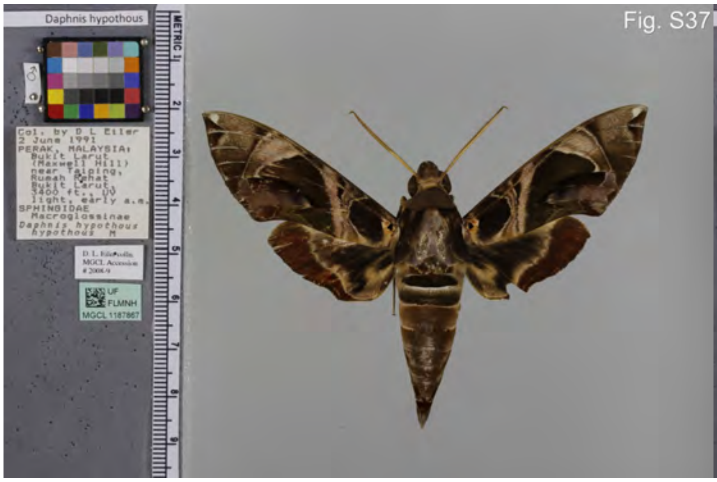

Daphnis_hypothous_MGCL_1187867_D.JPG

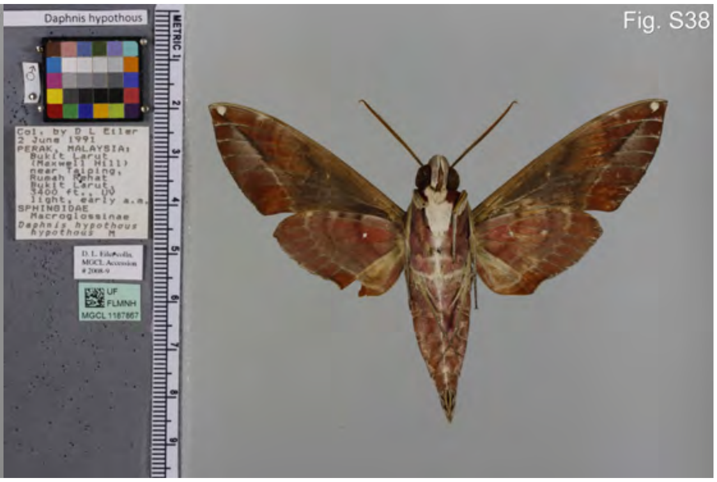

Daphnis_hypothous_MGCL_1187867_V.JPG

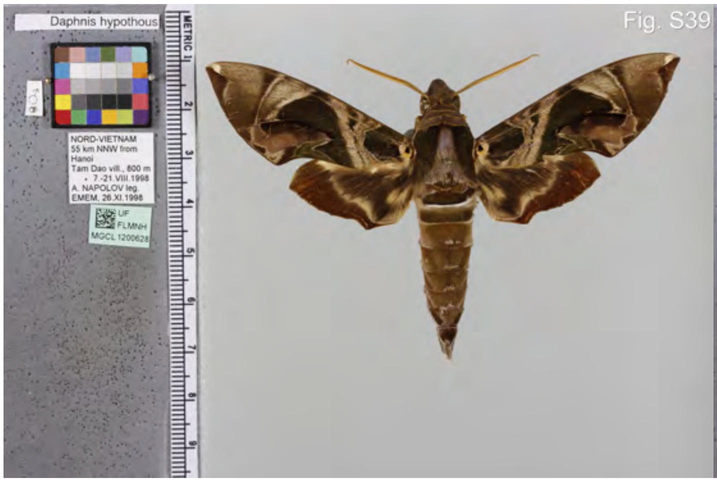

Daphnis_hypothous_MGCL_1200628_D.JPG

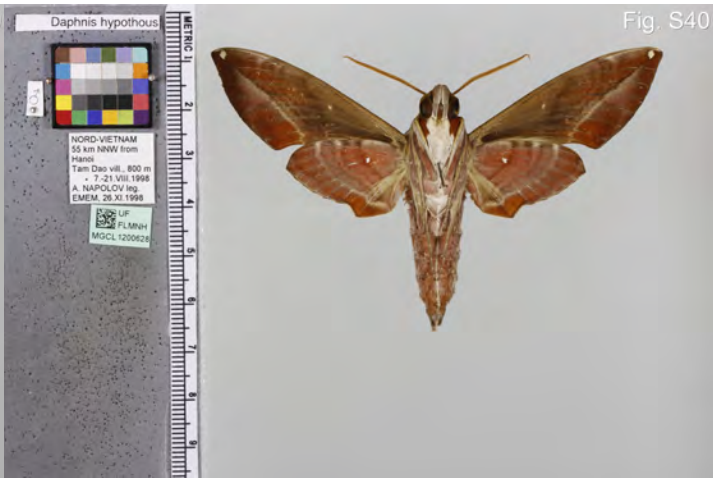

Daphnis_hypothous_MGCL_1200628_V.JPG

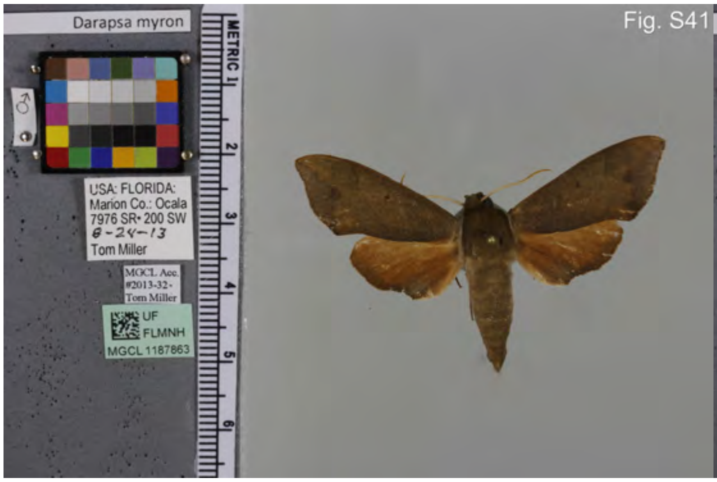

Darapsa_myron_MGCL_1187863_D.JPG

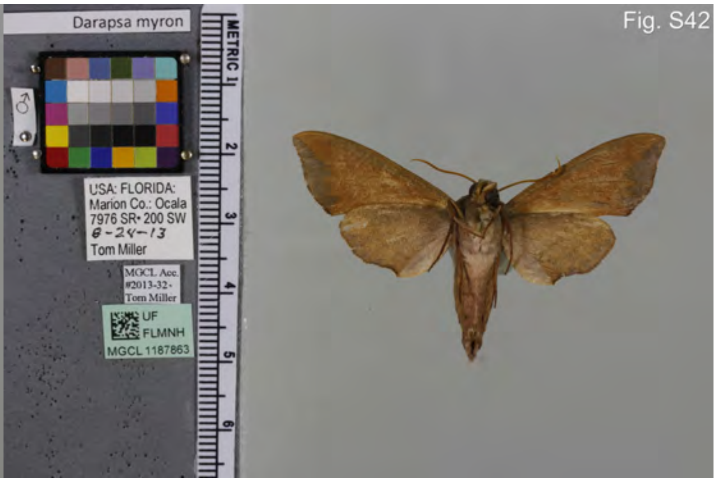

Darapsa_myron_MGCL_1187863_V.JPG

Habitus images of MGCL voucher specimens

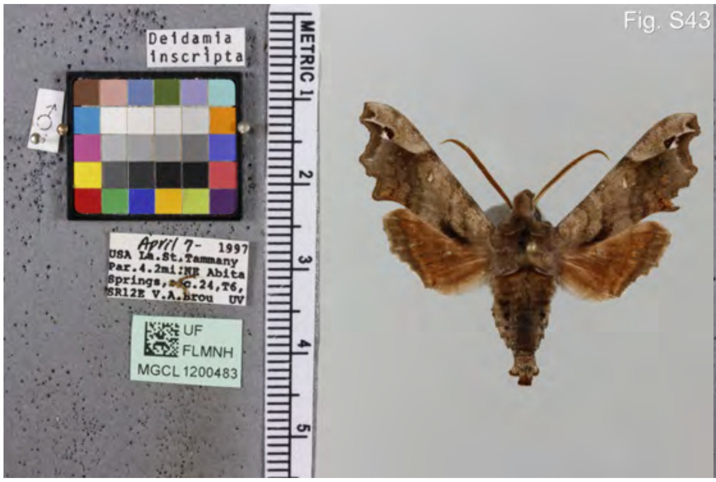

Deidamia_inscriptum_MGCL_1200483_D.JPG

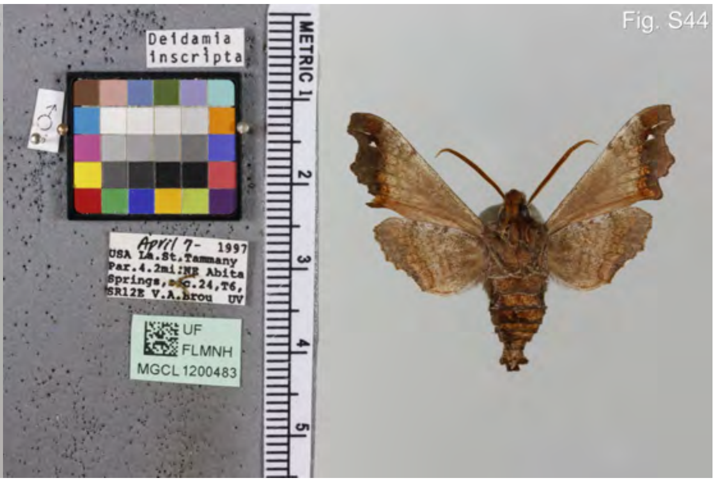

Deidamia_inscriptum_MGCL_1200483_V.JPG

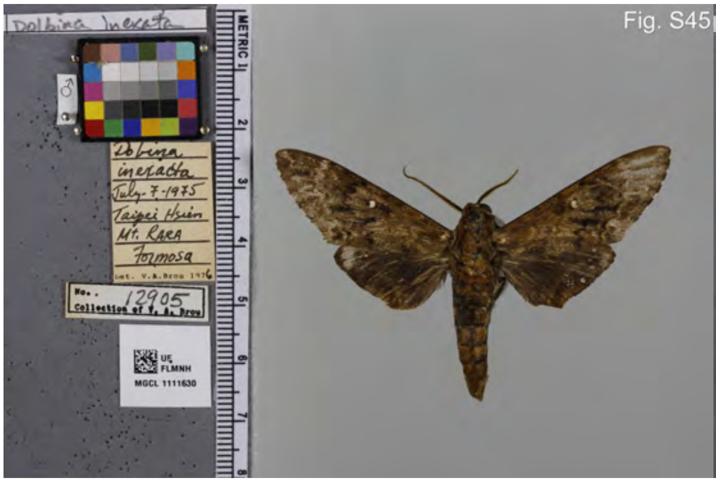

Dolbina_inexata_MGCL_1111630_D.JPG

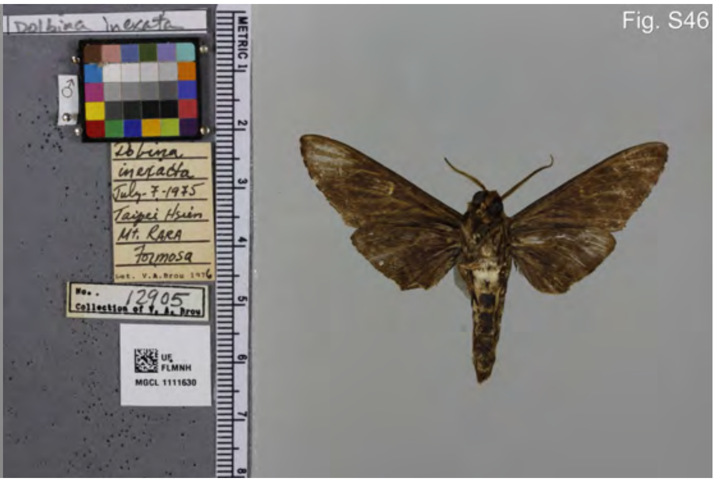

Dolbina_inexata_MGCL_1111630_V.JPG

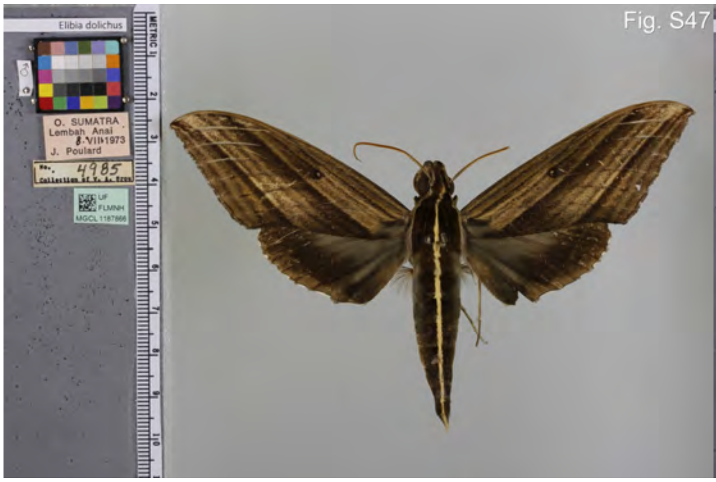

Elibia_dolichus_MGCL_1187866_D.JPG

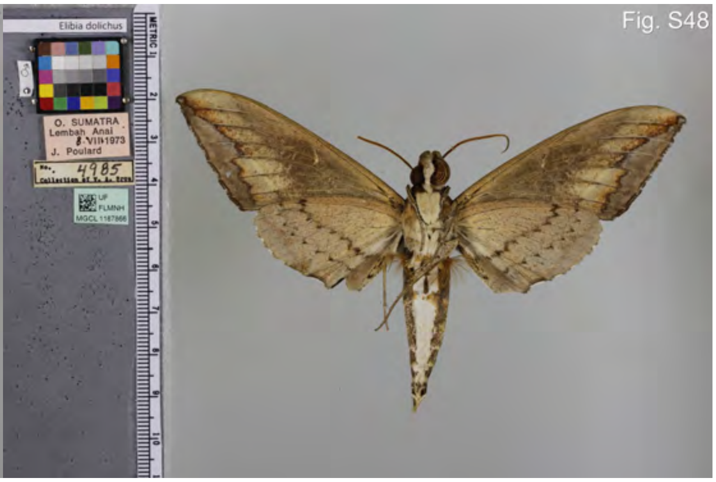

Elibia_dolichus_MGCL_1187866_V.JPG

Habitus images of MGCL voucher specimens

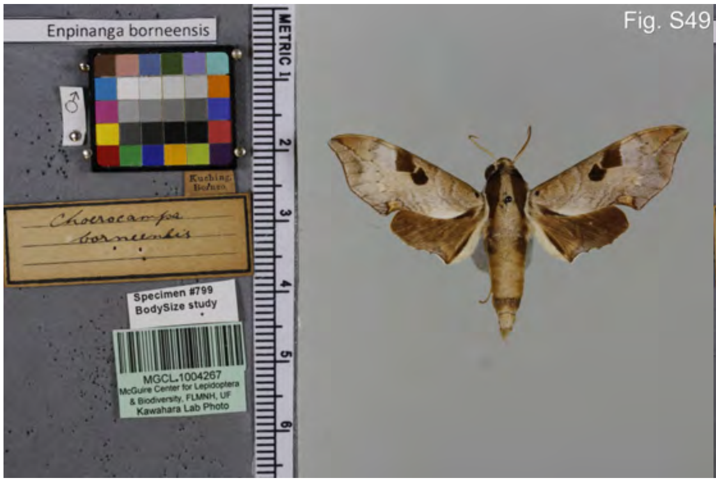

Enpinanga_borneensis_MGCL_1004267_D.JPG

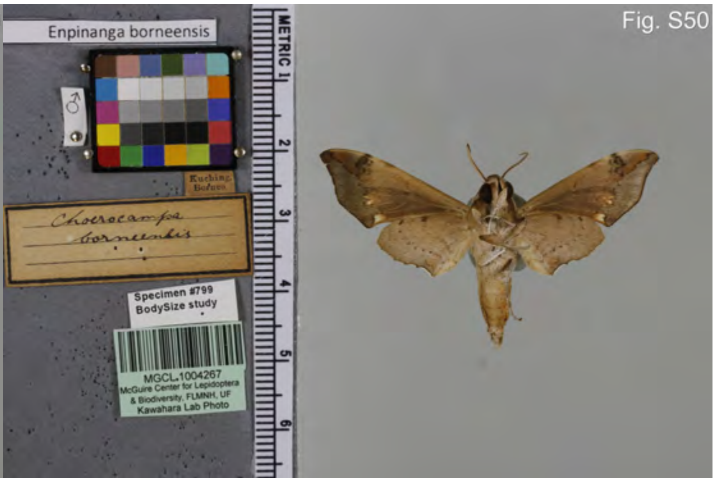

Enpinanga_borneensis_MGCL_1004267_V.JPG

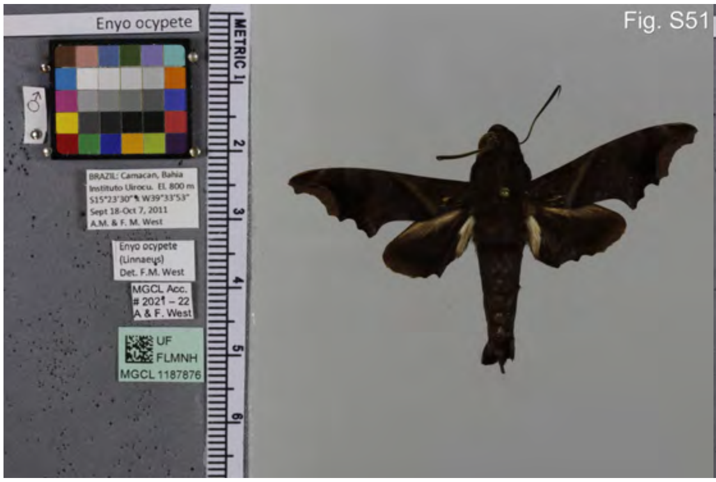

Enyo_ocypete_MGCL_1187876_D.JPG

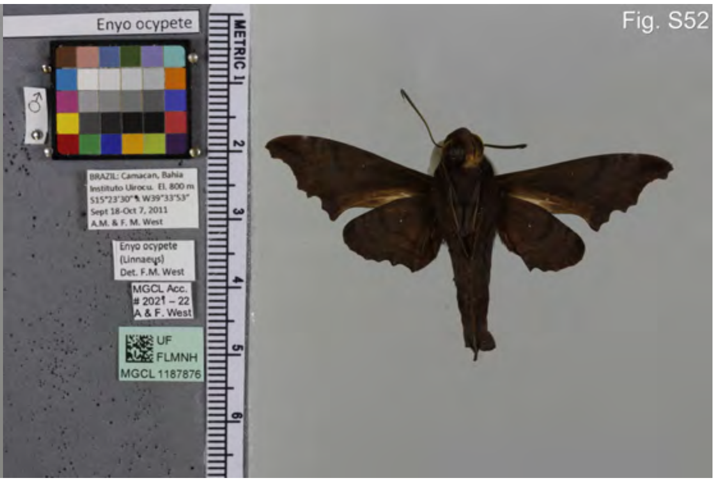

Enyo_ocypete_MGCL_1187876_V.JPG

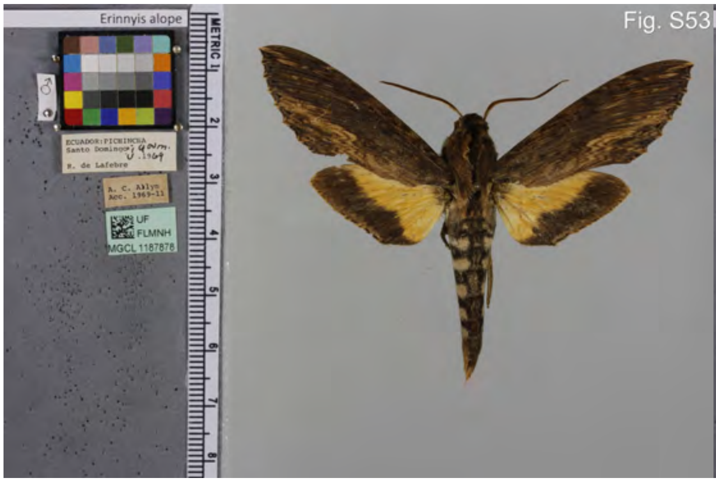

Erinnyis_alope_MGCL_1187878_D.JPG

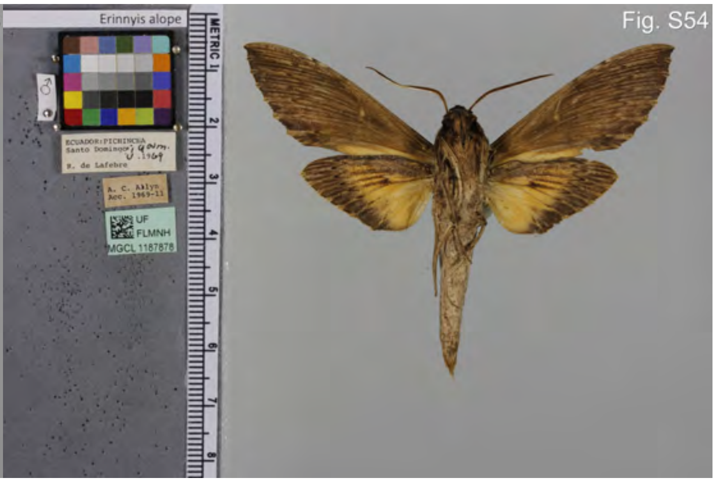

Erinnyis_alope_MGCL_1187878_V.JPG

Habitus images of MGCL voucher specimens

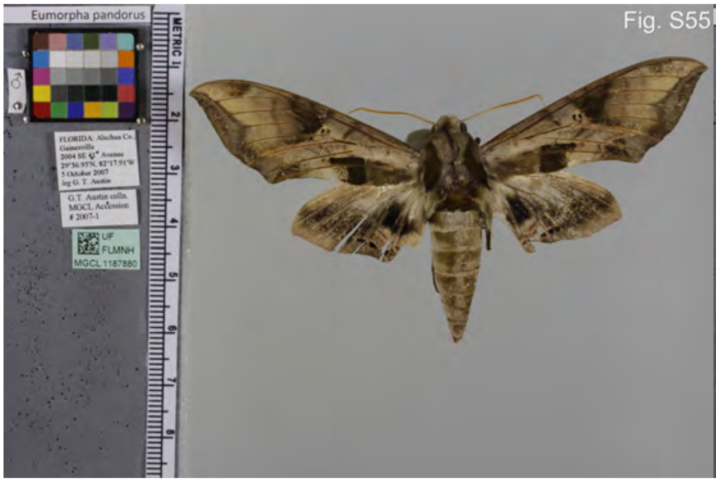

Eumorpha_pandorus_MGCL_1187880_D.JPG

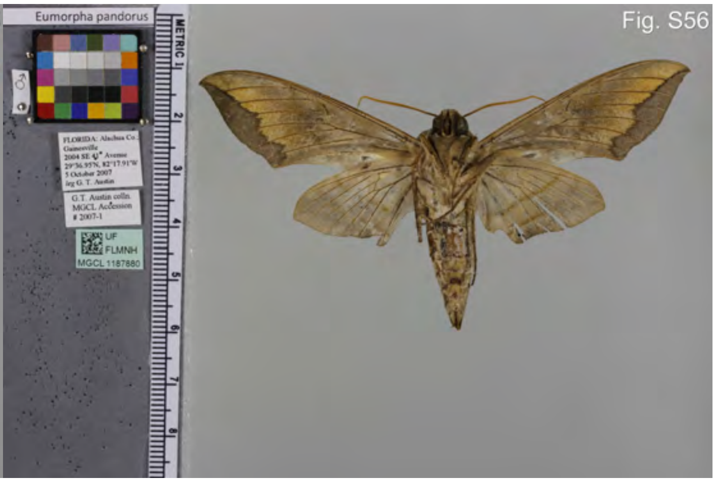

Eumorpha_pandorus_MGCL_1187880_V.JPG

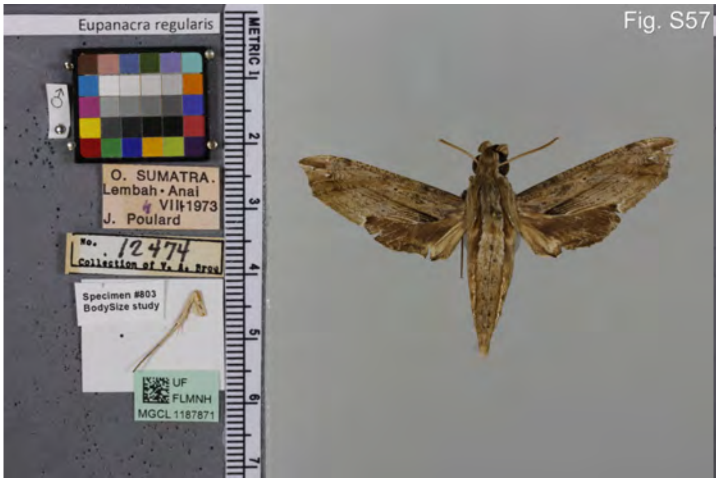

Eupanacra_regularis_MGCL_1187871_D.JPG

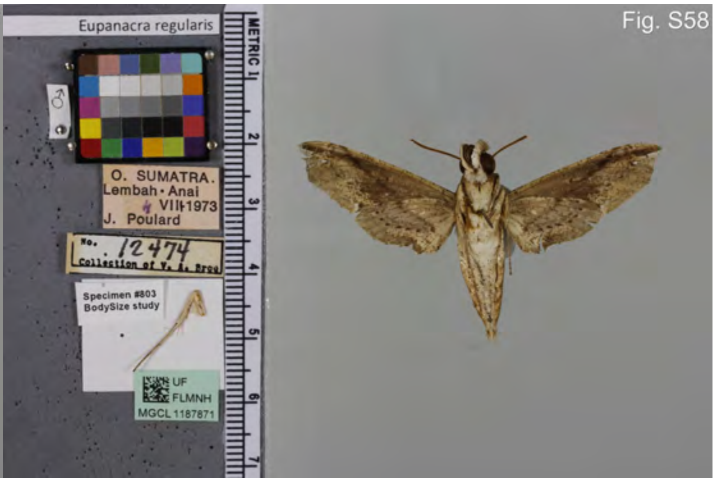

Eupanacra_regularis_MGCL_1187871_V.JPG

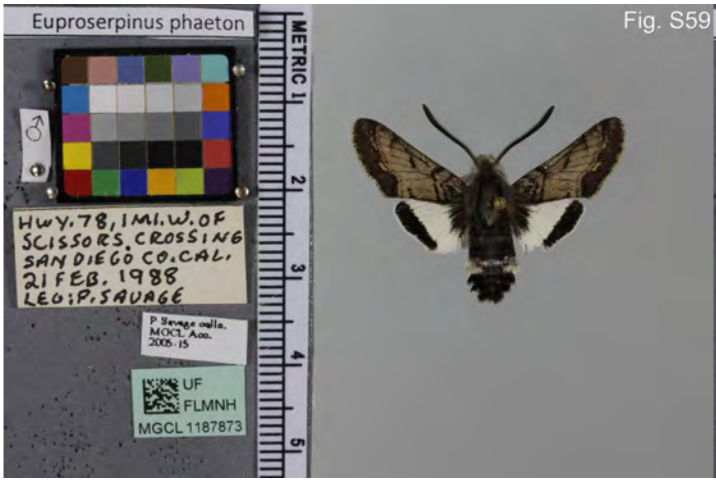

Euproserpinus_phaeton_MGCL_1187873_D.JPG

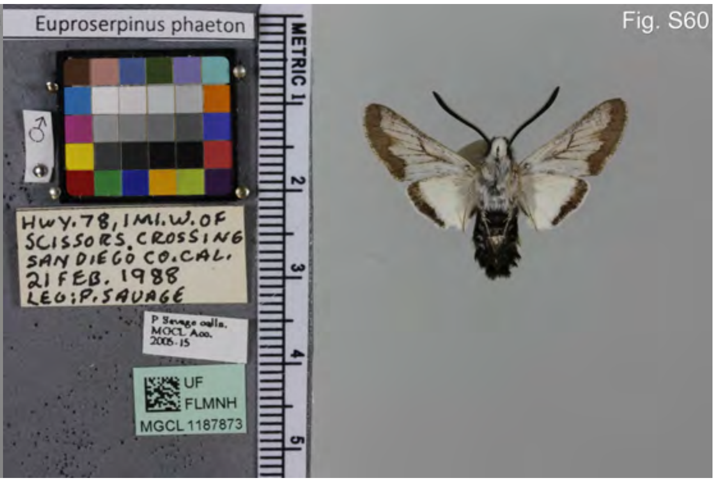

Euproserpinus_phaeton_MGCL_1187873_V.JPG

Habitus images of MGCL voucher specimens

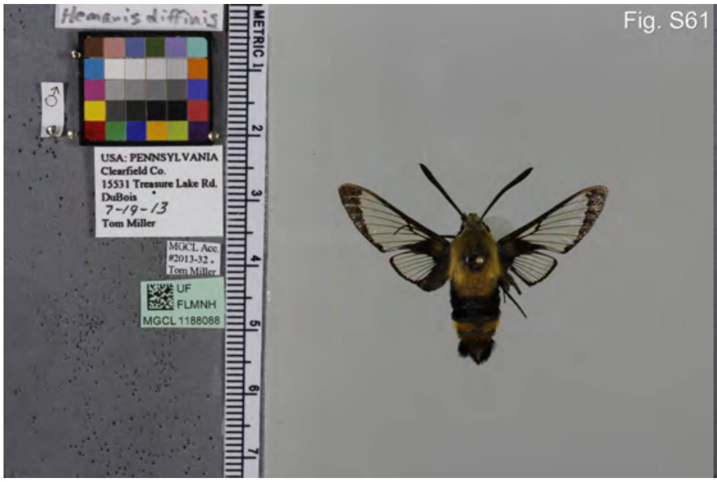

Hemaris_diffinis_MGCL_1188088_D.JPG

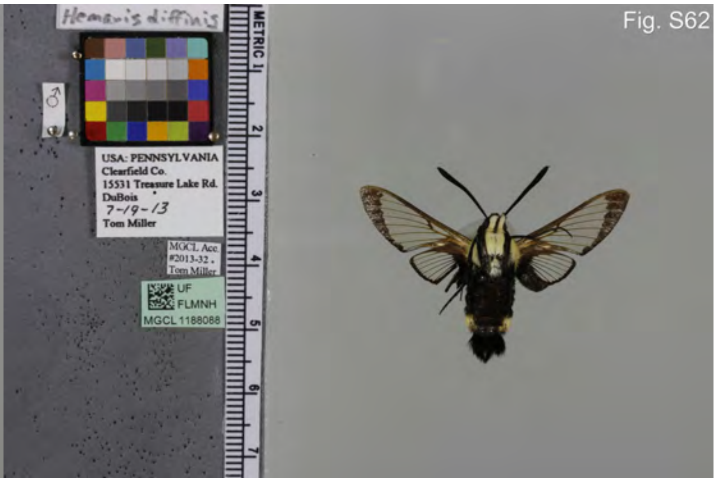

Hemaris_diffinis_MGCL_1188088_V.JPG

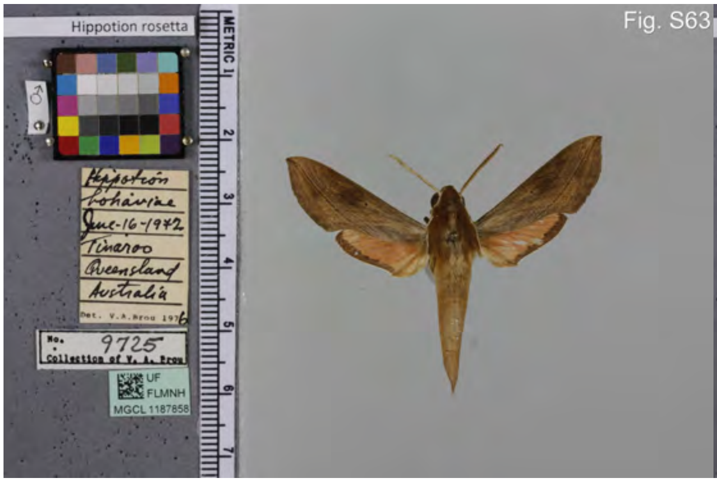

Hippotion_rosetta_MGCL_1187858_D.JPG

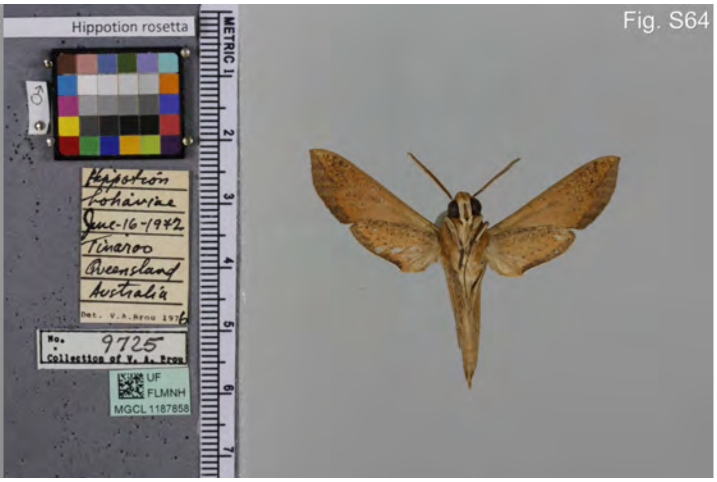

Hippotion_rosetta_MGCL_1187858_V.JPG

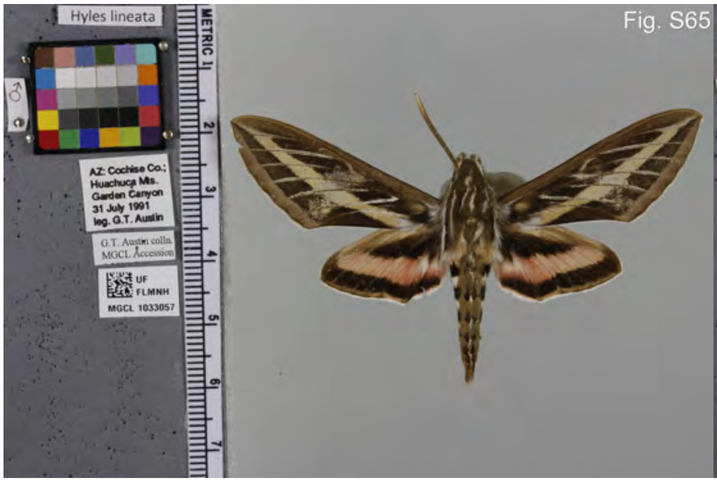

Hyles_lineata_MGCL_1033057_D.JPG

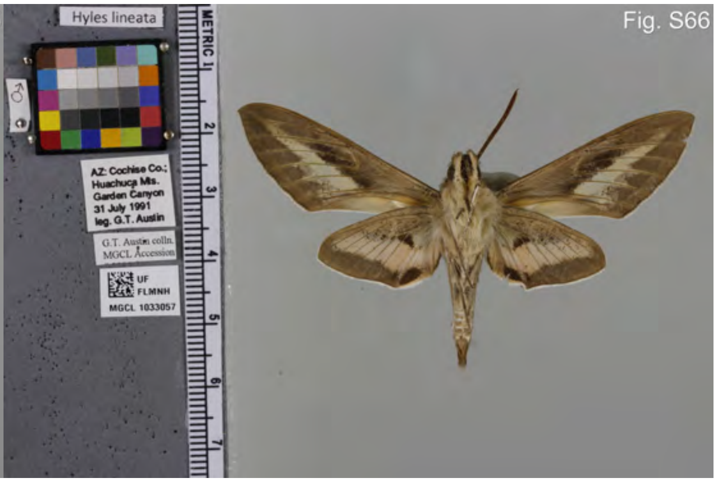

Hyles_lineata_MGCL_1033057_V.JPG

Habitus images of MGCL voucher specimens

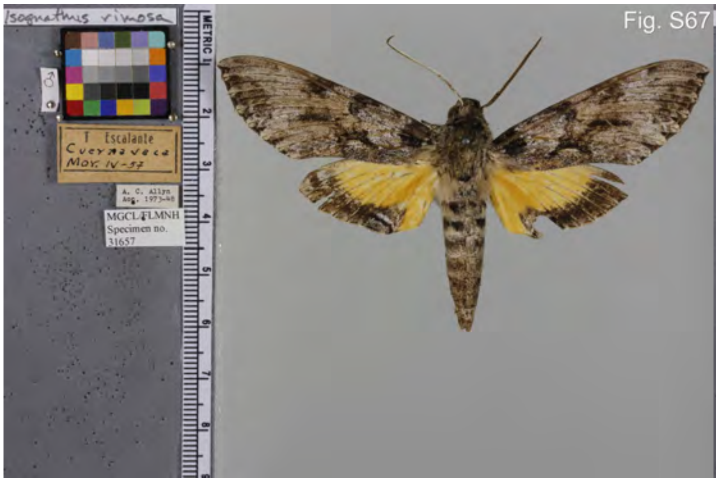

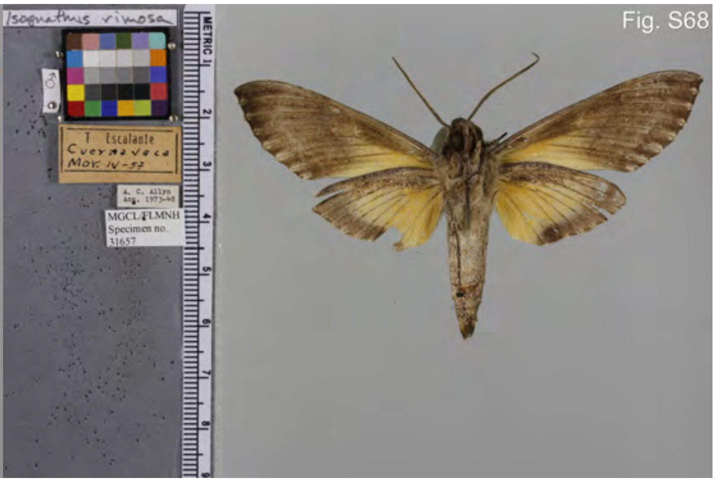

Isognathus_rimosa_MGCL_31657_D.JPG

Isognathus_rimosa_MGCL_31657_V.JPG

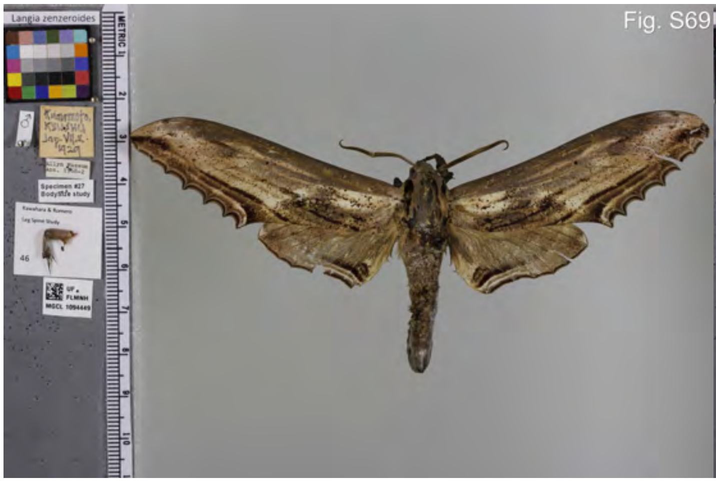

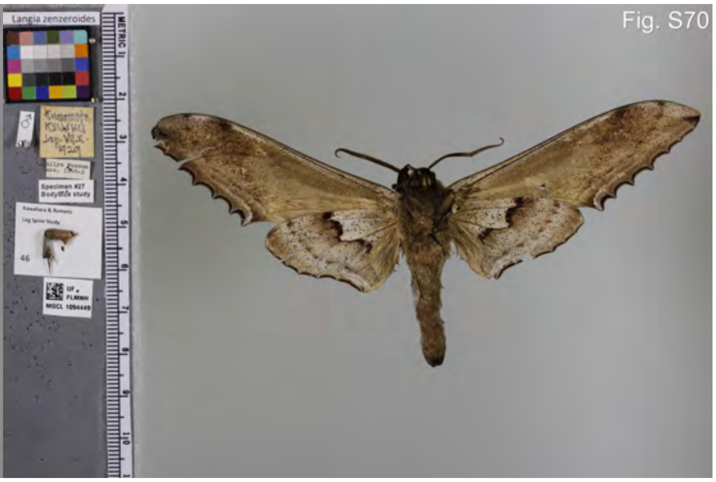

Langia_zenzeroides_MGCL_1094449_D.JPG

Langia_zenzeroides_MGCL_1094449_V.JPG

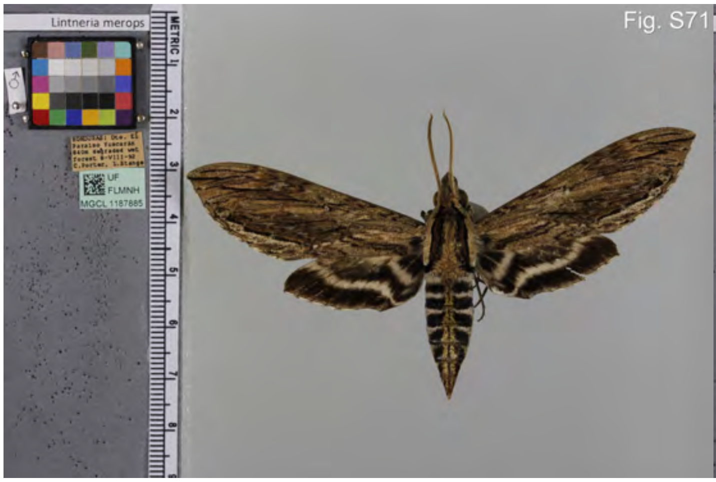

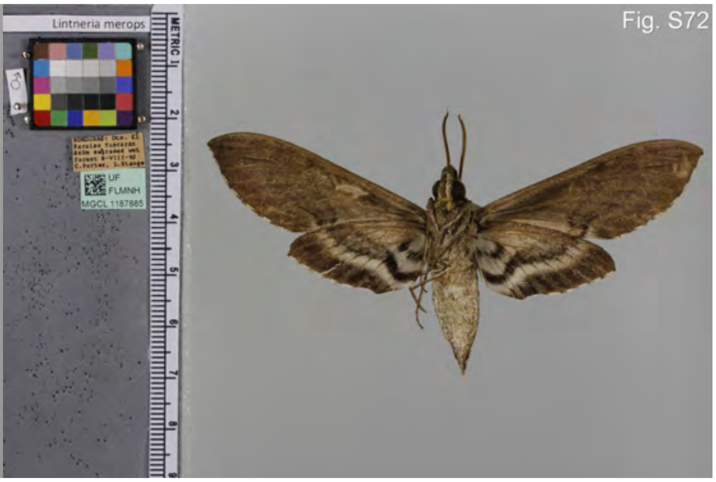

Lintneria_merops_MGCL_1187885_D.JPG

Lintneria_merops_MGCL_1187885_V.JPG

Habitus images of MGCL voucher specimens

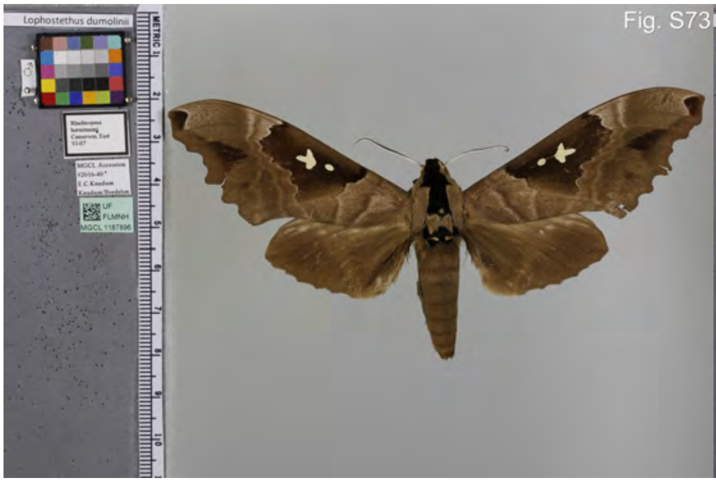

Lophostethus_dumolinii_MGCL_1187896_D.JPG

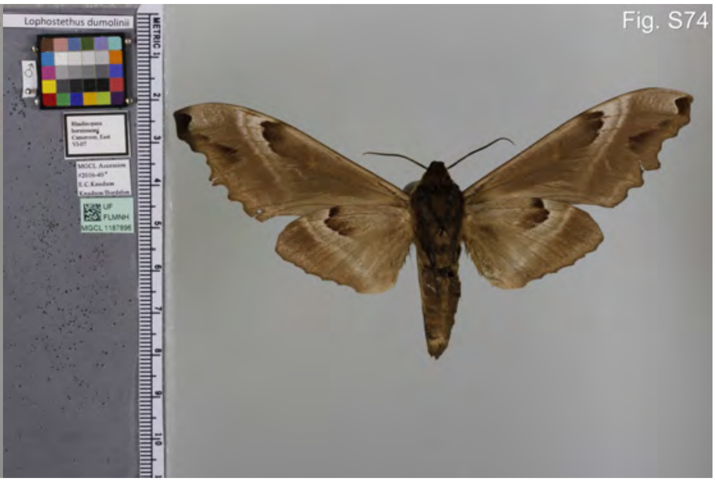

Lophostethus_dumolinii_MGCL_1187896_V.JPG

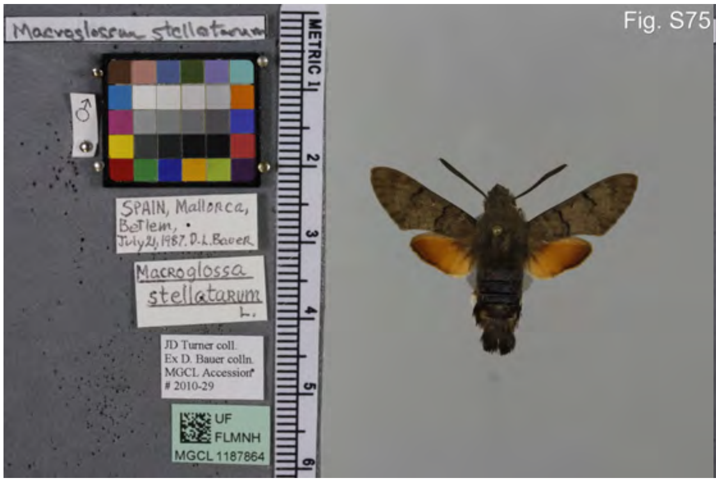

Macroglossum_stellatarum_MGCL_1187864_D.JPG

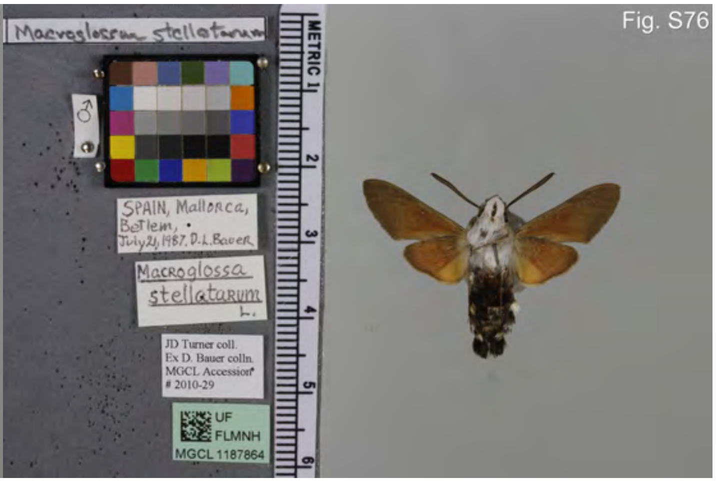

Macroglossum_stellatarum_MGCL_1187864_V.JPG

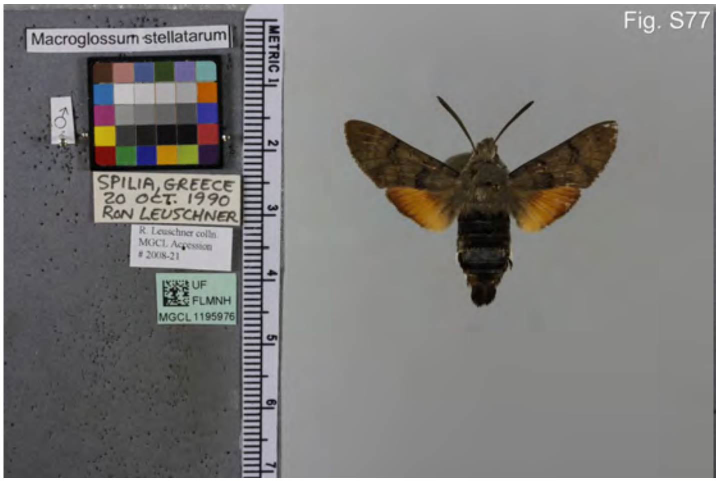

Macroglossum_stellatarum_MGCL_1195976_D.JPG

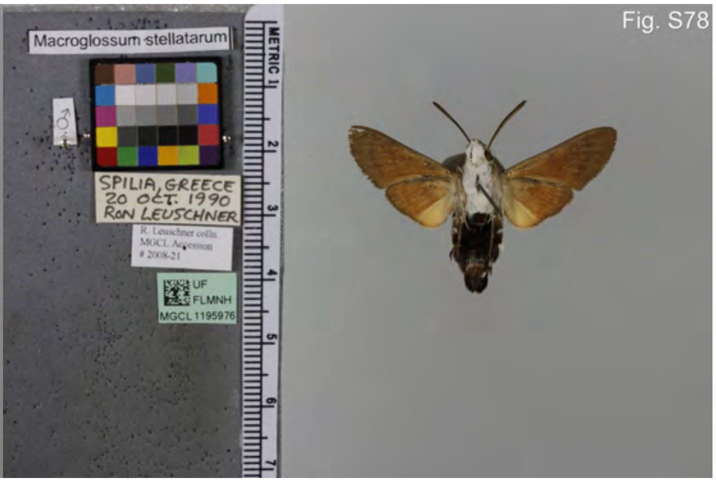

Macroglossum_stellatarum_MGCL_1195976_V.JPG

Habitus images of MGCL voucher specimens

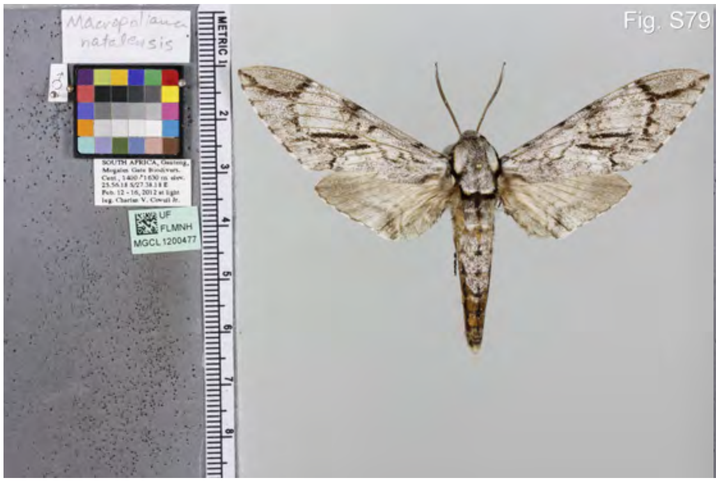

Macropoliana_natalensis_MGCL_1200477_D.JPG

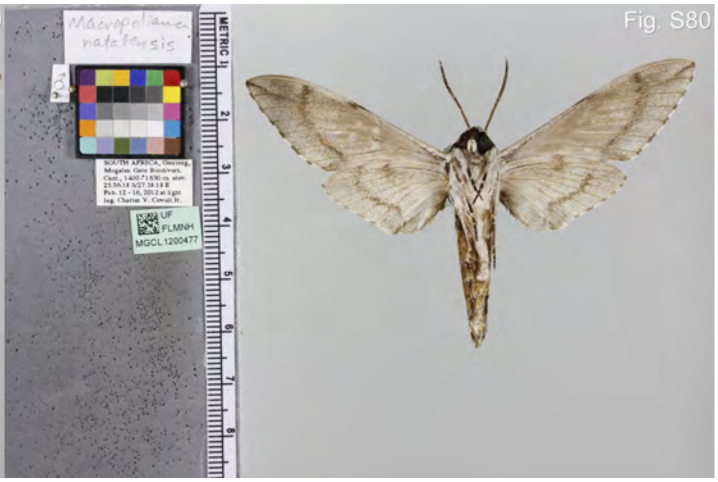

Macropoliana_natalensis_MGCL_1200477_V.JPG

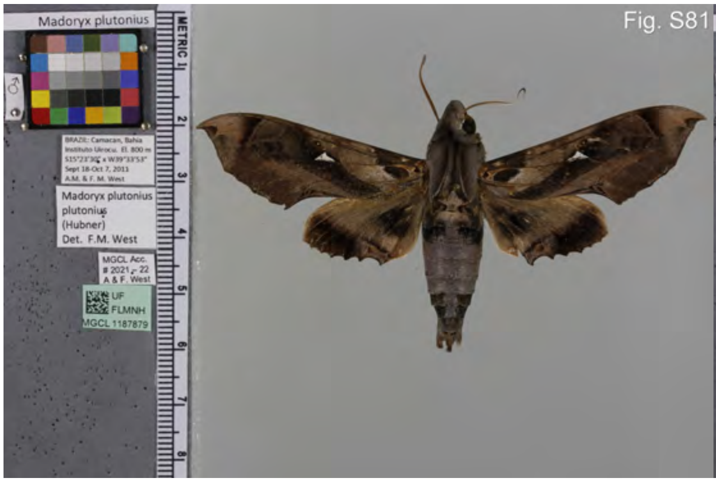

Madoryx_plutonius_MGCL_1187879_D.JPG

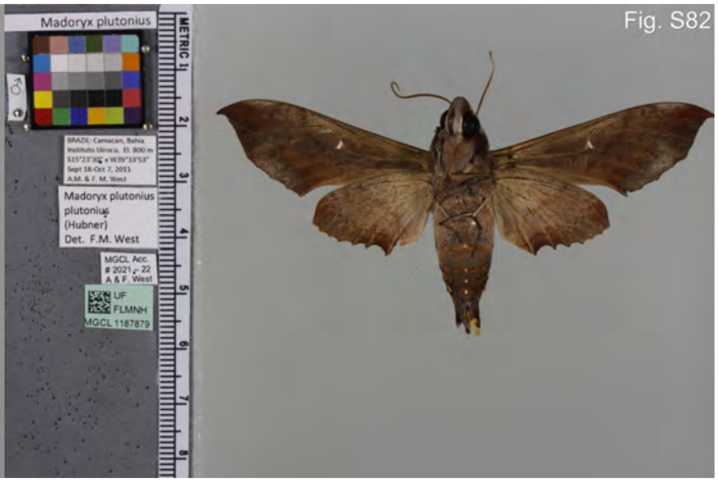

Madoryx_plutonius_MGCL_1187879_V.JPG

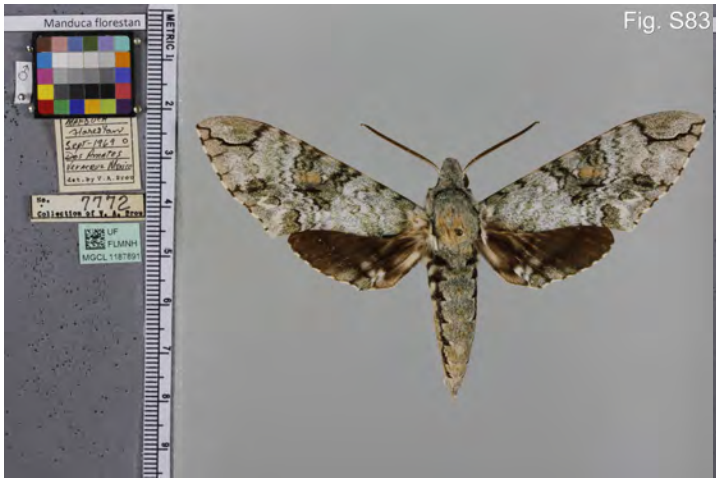

Manduca_florestan_MGCL_1187891_D.JPG

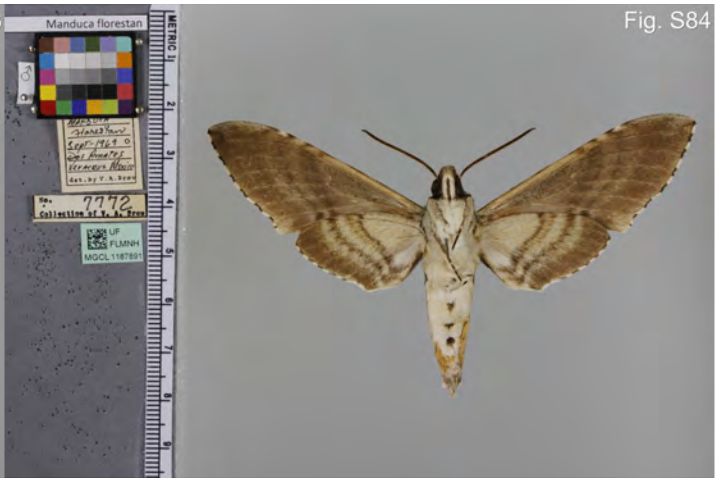

Manduca_florestan_MGCL_1187891_V.JPG

Habitus images of MGCL voucher specimens

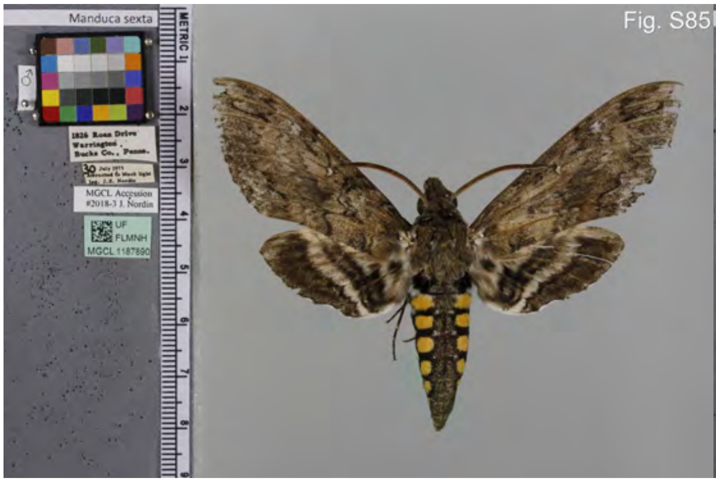

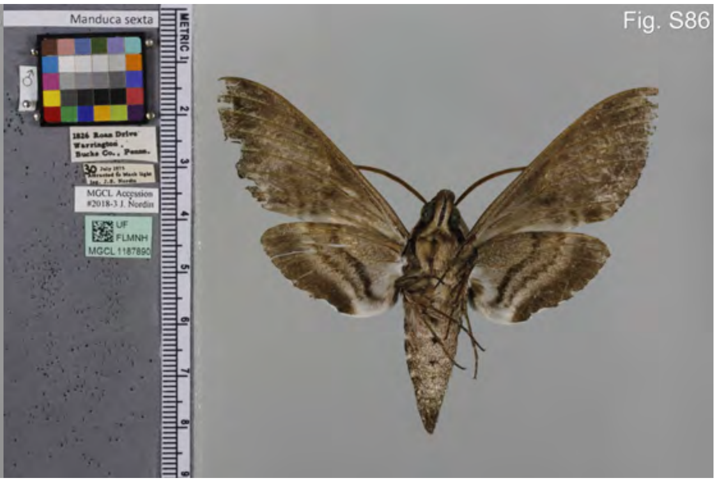

Manduca_sexta_MGCL_1187890_D.JPG

Manduca_sexta_MGCL_1187890_V.JPG

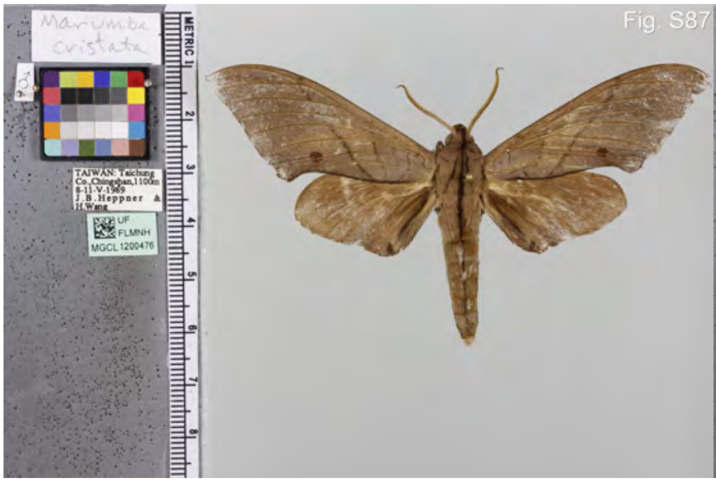

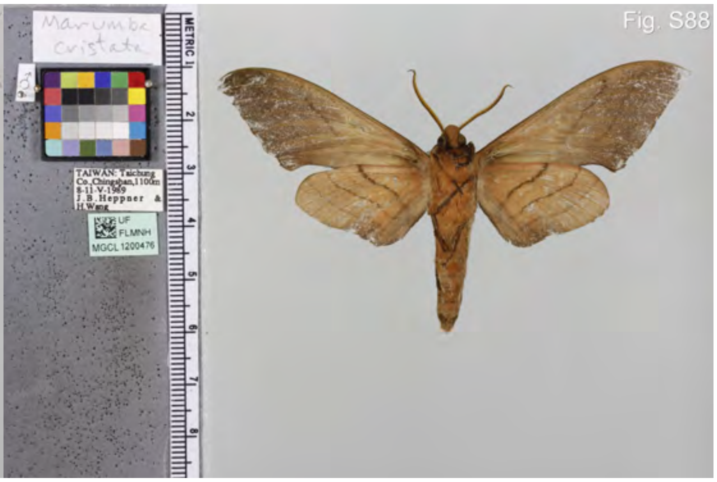

Marumba_cristata_MGCL_1200476_D.JPG

Marumba_cristata_MGCL_1200476_V.JPG

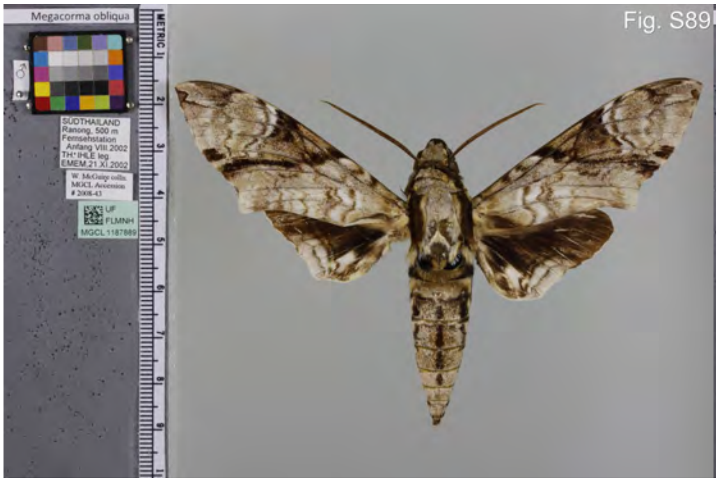

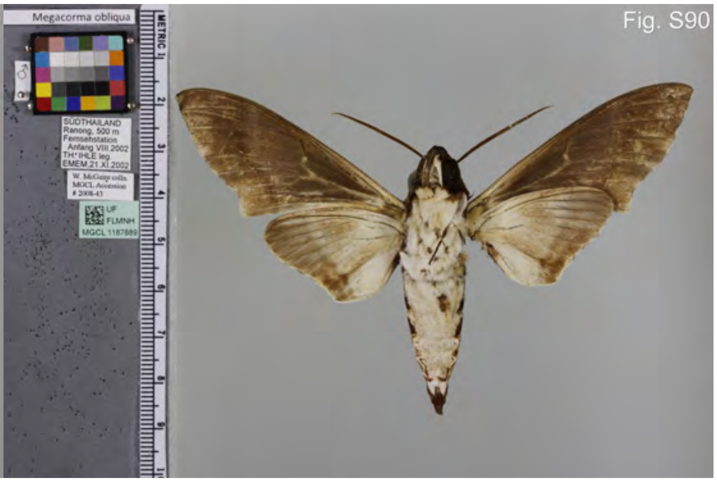

Megacorma_obliqua_MGCL_1187889_D.JPG

Megacorma_obliqua_MGCL_1187889_V.JPG

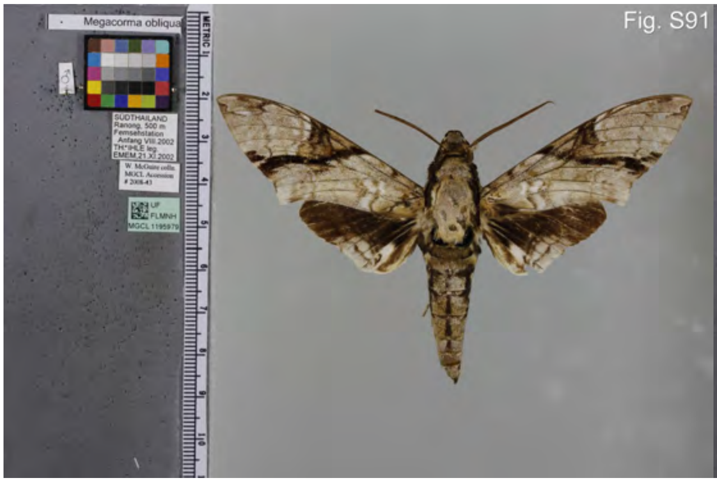

Megacorma_obliqua_MGCL_1195979_D.JPG

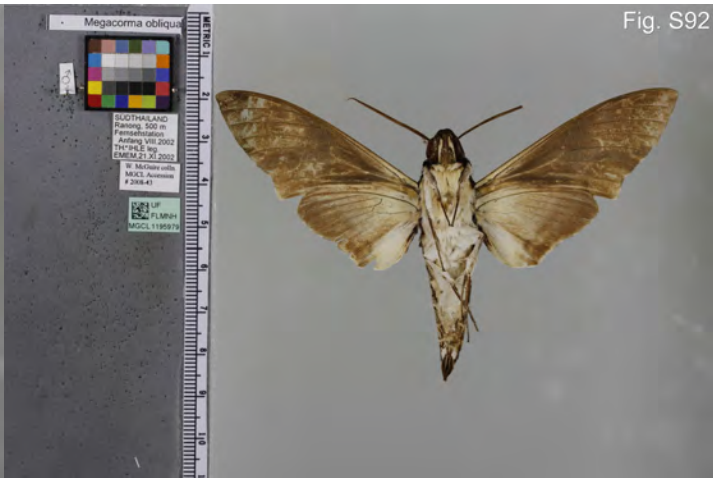

Megacorma_obliqua_MGCL_1195979_V.JPG

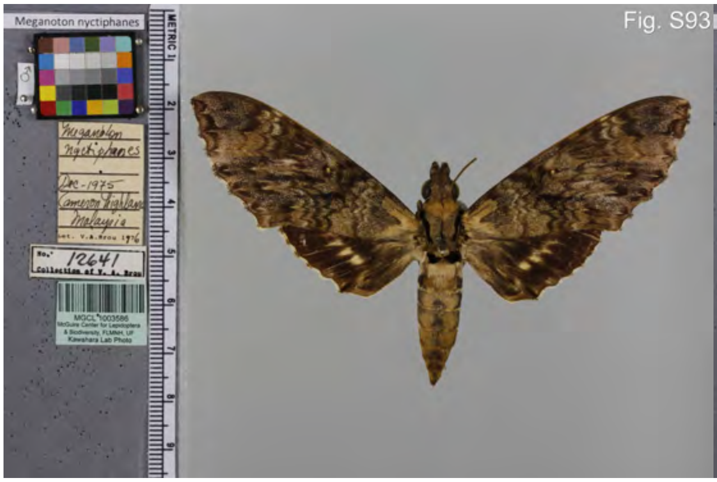

Meganoton_nyctiphanes_MGCL_1003586_D.JPG

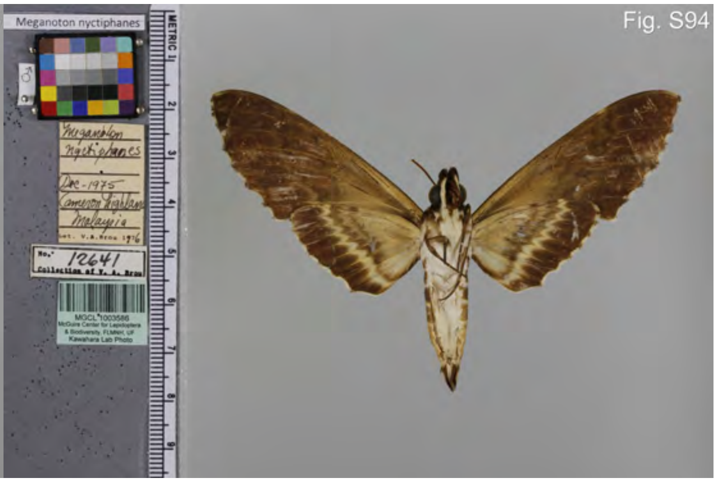

Meganoton_nyctiphanes_MGCL_1003586_V.JPG

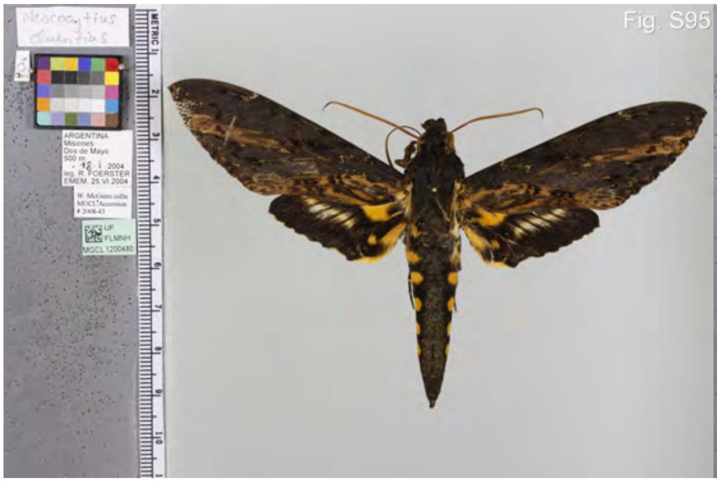

Neococytius_cluentius_MGCL_1200480_D.JPG

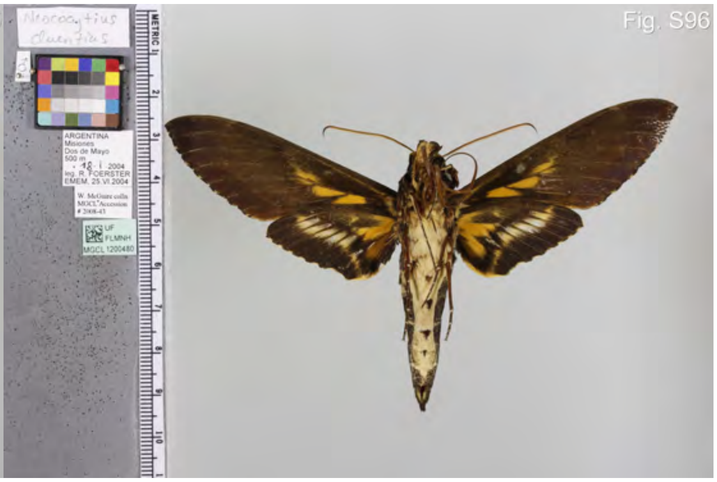

Neococytius_cluentius_MGCL_1200480_V.JPG

Habitus images of MGCL voucher specimens

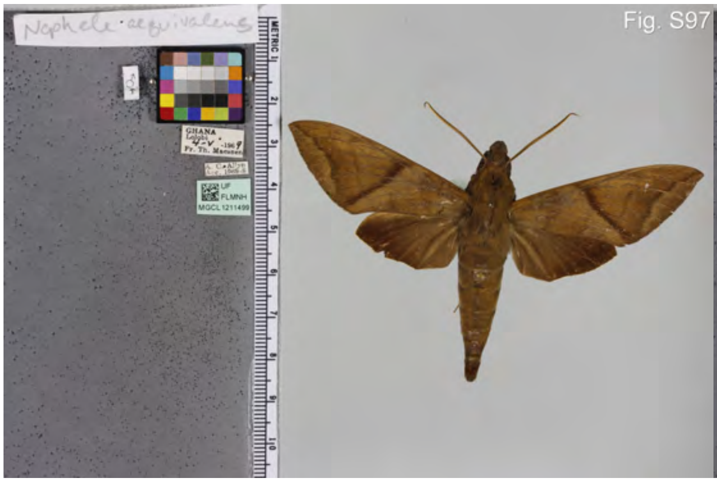

Nephele_aequivalens_MGCL_1211499_D.JPG

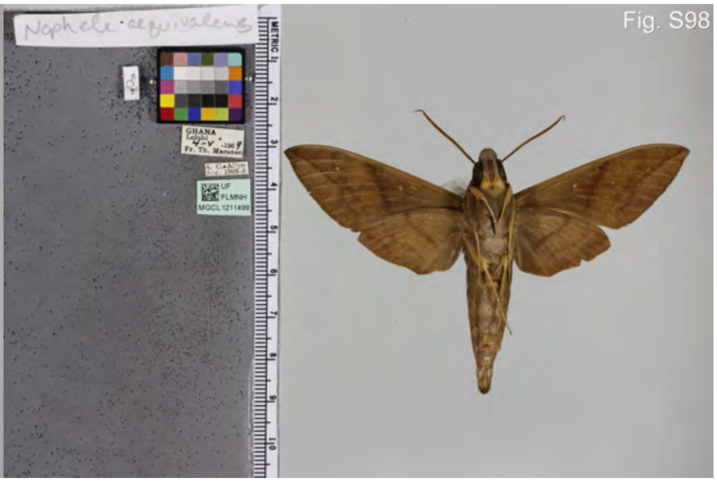

Nephele_aequivalens_MGCL_1211499_V.JPG

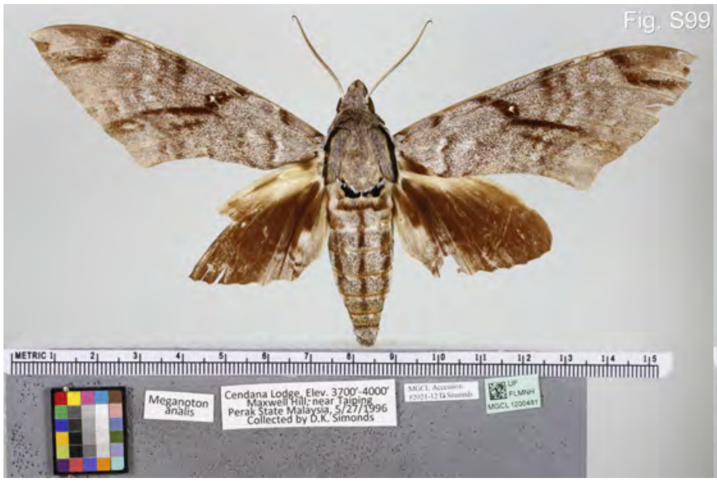

Notonagemia_analis_MGCL_1200481_D.JPG

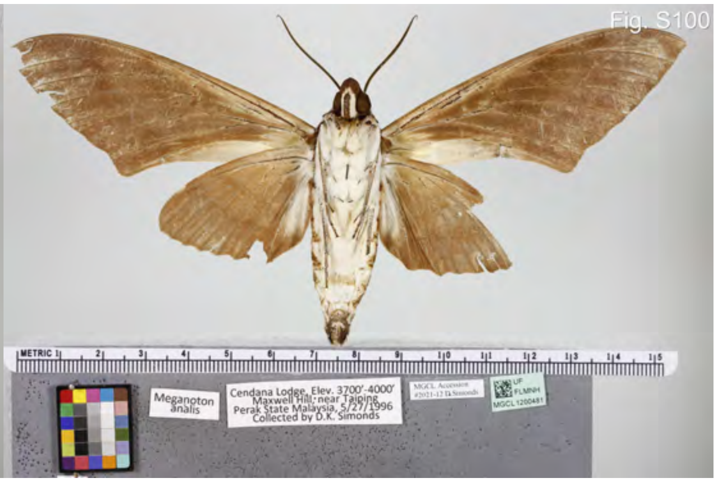

Notonagemia_analis_MGCL_1200481_V.JPG

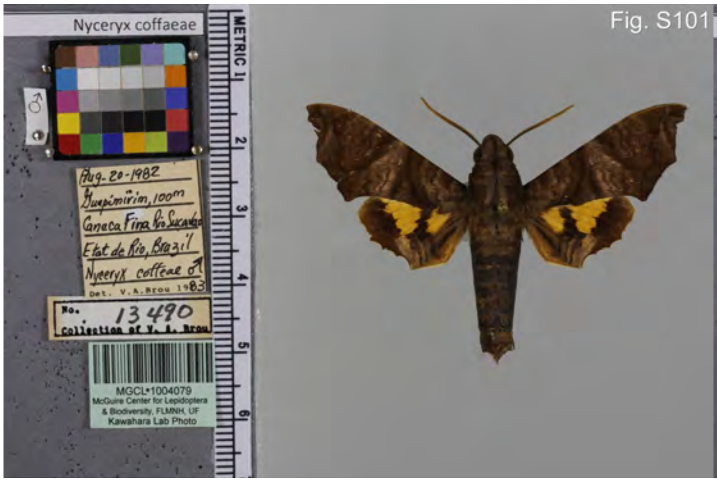

Nyceryx_coffaeae_MGCL_1004079_D.JPG

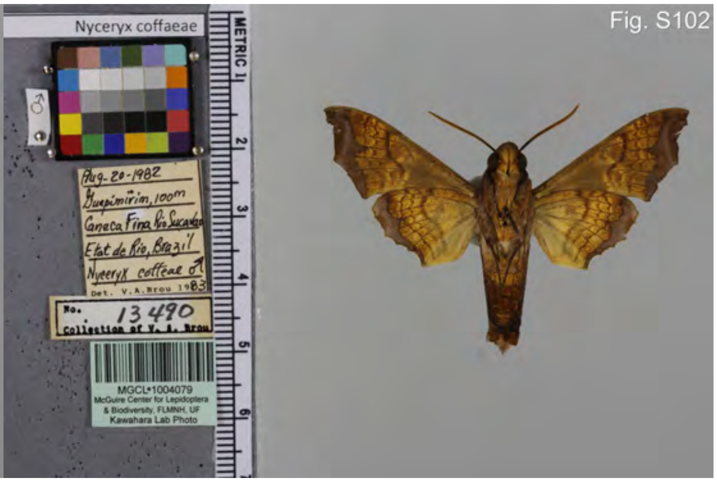

Nyceryx_coffaeae_MGCL_1004079_V.JPG

Habitus images of MGCL voucher specimens

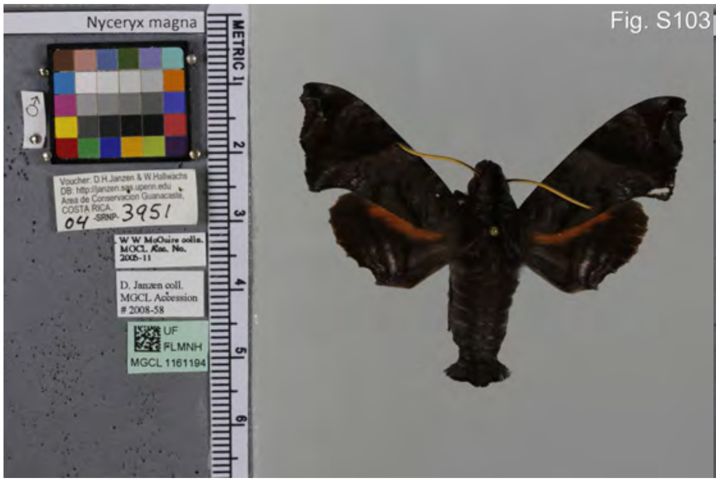

Nyceryx_magna_MGCL_1161194_D.JPG

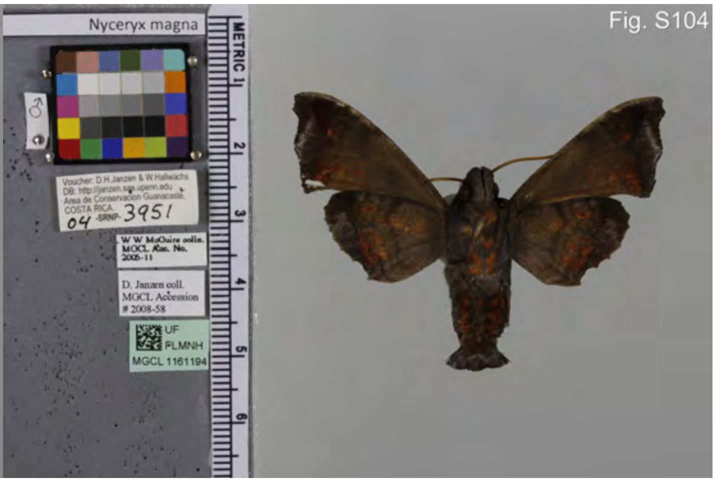

Nyceryx_magna_MGCL_1161194_V.JPG

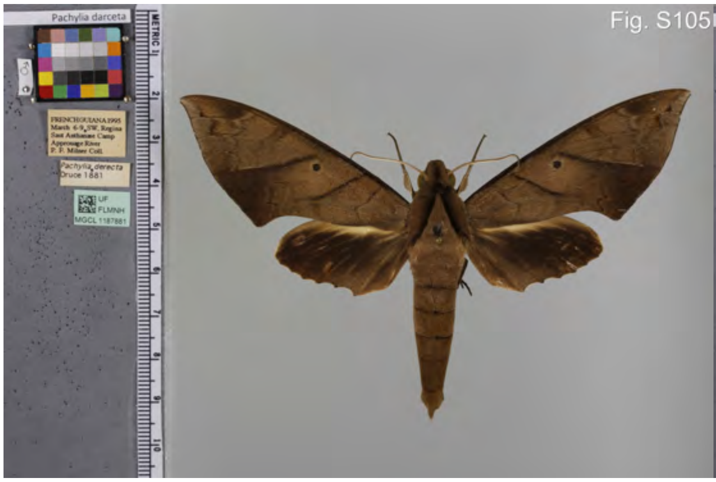

Pachylia_darceta_MGCL_1187881_D.JPG

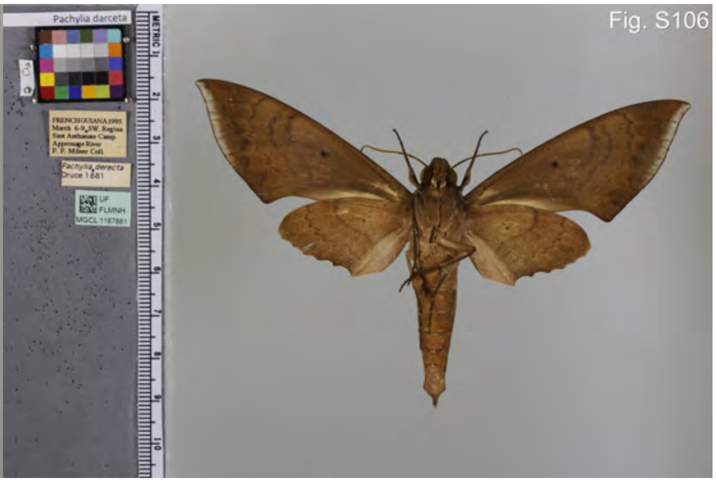

Pachylia_darceta_MGCL_1187881_V.JPG

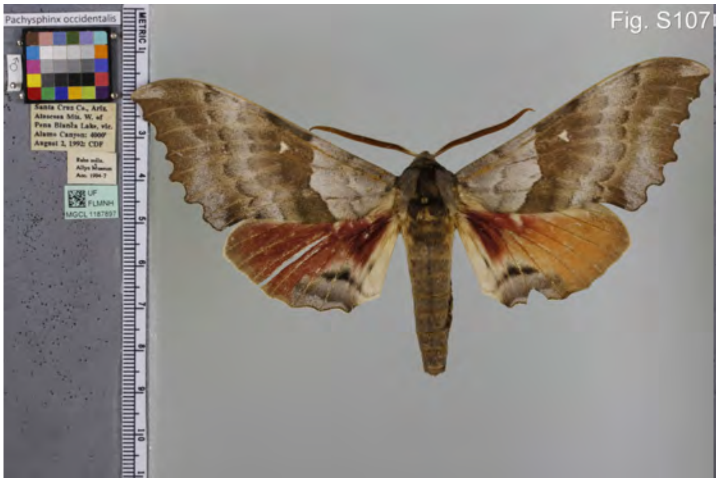

Pachysphinx_occidentalis_MGCL_1187897_D.JPG

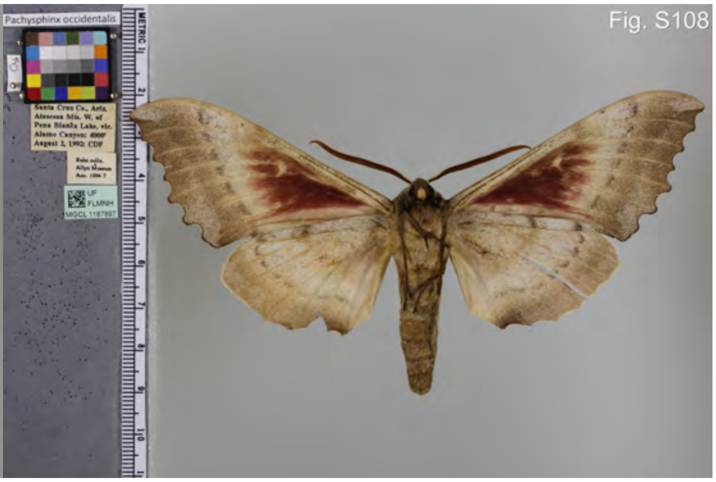

Pachysphinx_occidentalis_MGCL_1187897_V.JPG

Habitus images of MGCL voucher specimens

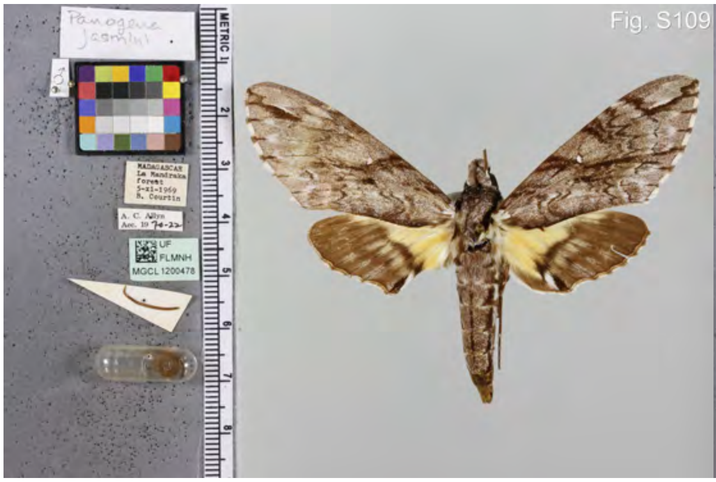

Panogena_jasmini_MGCL_1200478_D.JPG

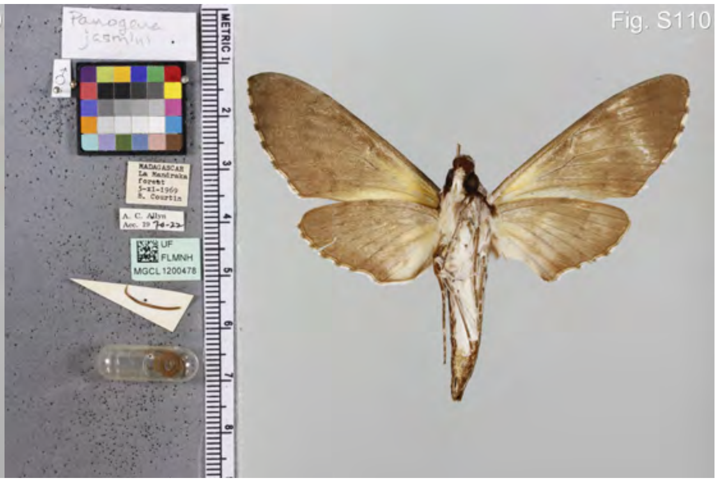

Panogena_jasmini_MGCL_1200478_V.JPG

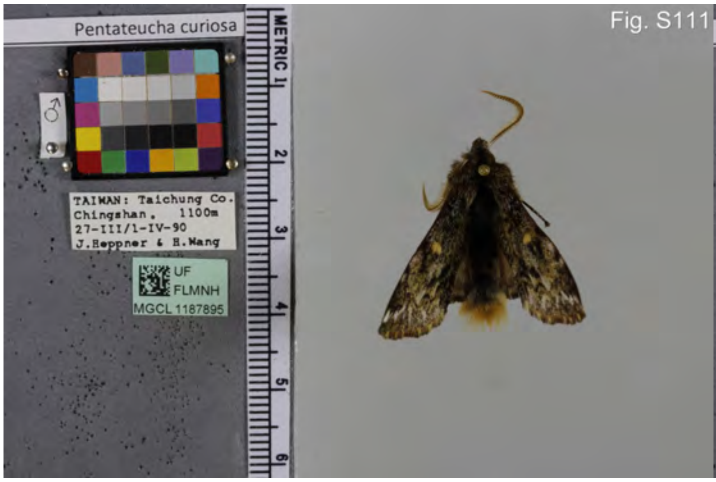

Pentateucha_curiosa_MGCL_1187895_D.JPG

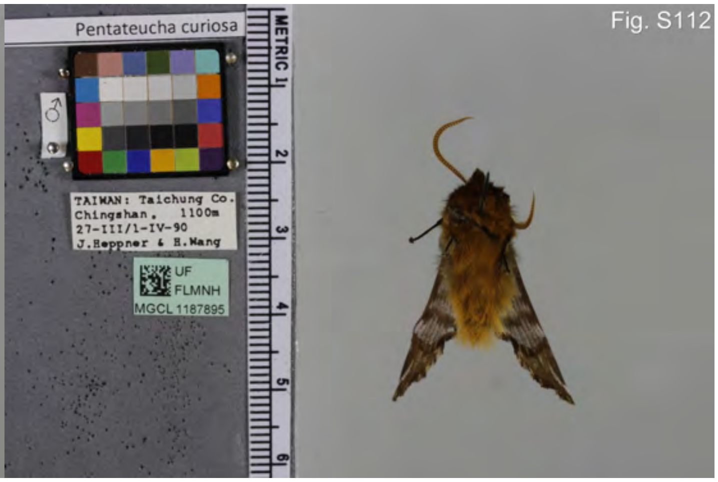

Pentateucha_curiosa_MGCL_1187895_V.JPG

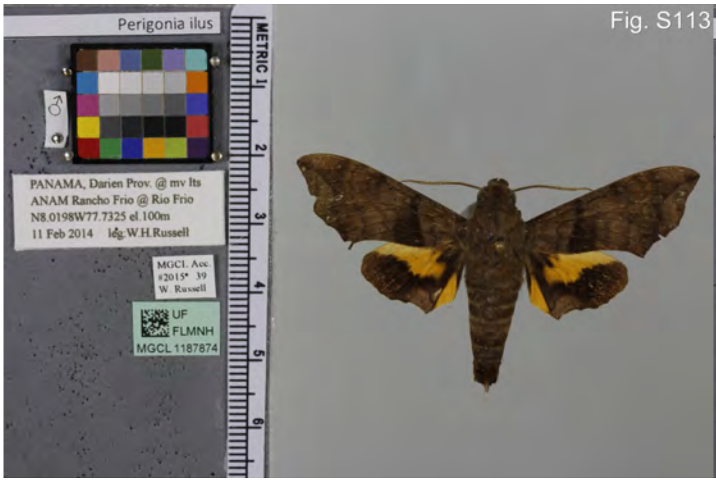

Perigonia_ilus_MGCL_1187874_D.JPG

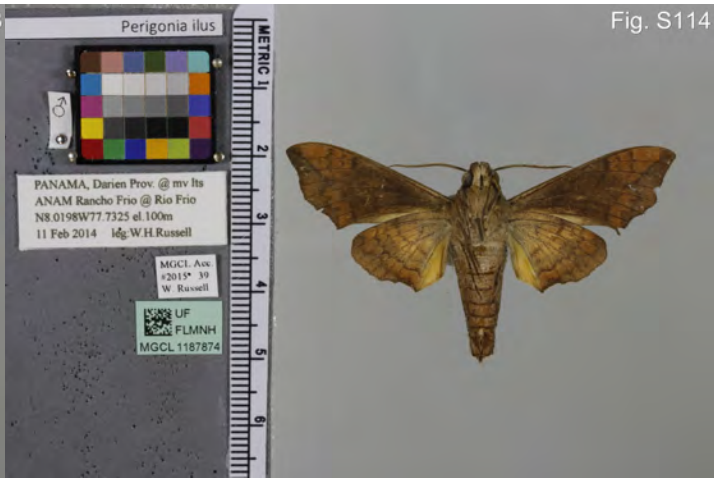

Perigonia_ilus_MGCL_1187874_V.JPG

Habitus images of MGCL voucher specimens

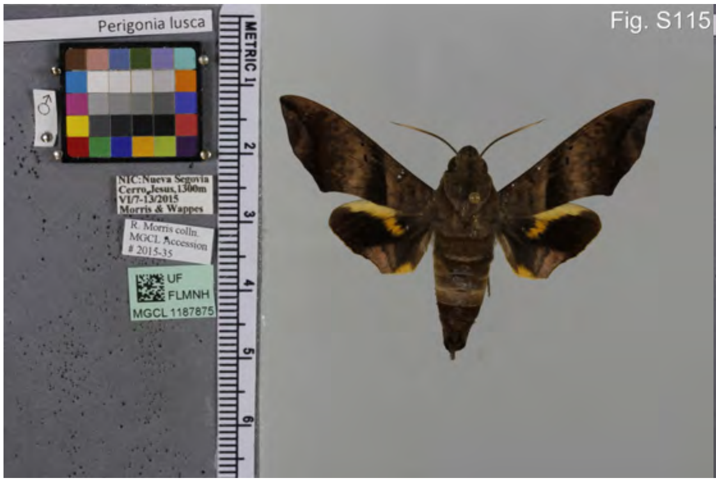

Perigonia_lusca_MGCL_1187875_D.JPG

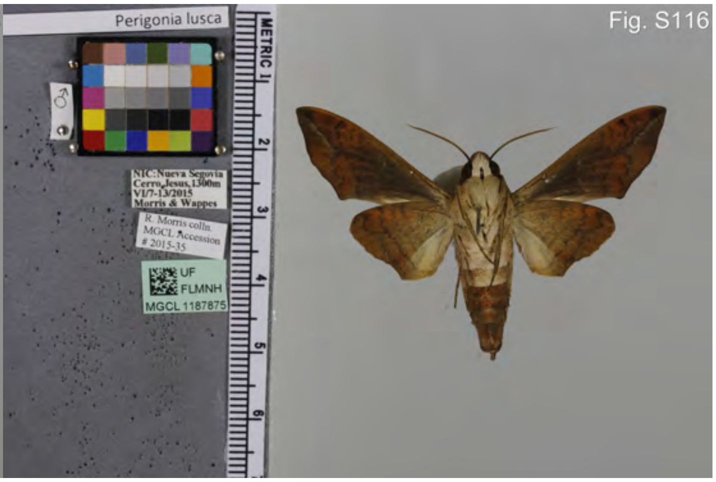

Perigonia_lusca_MGCL_1187875_V.JPG

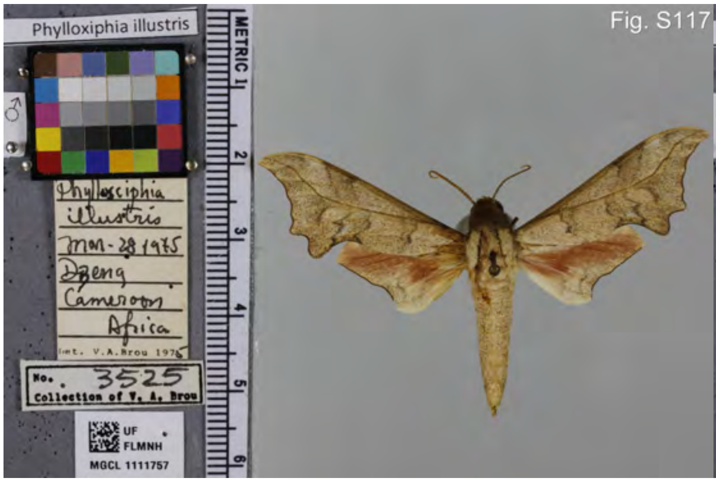

Phylloxiphia_illustris_MGCL_1111757_D.JPG

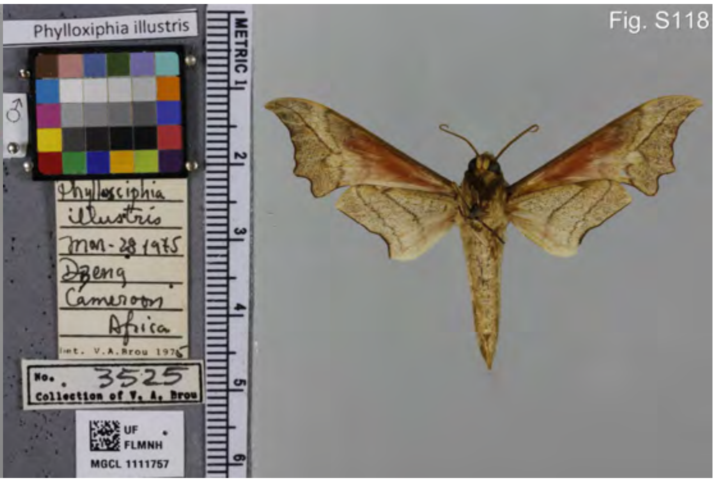

Phylloxiphia_illustris_MGCL_1111757_V.JPG

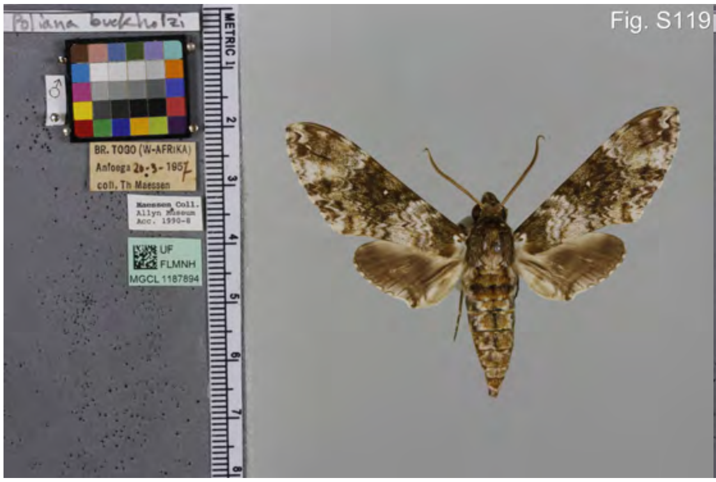

Poliana_buckholzi_MGCL_1187894_D.JPG

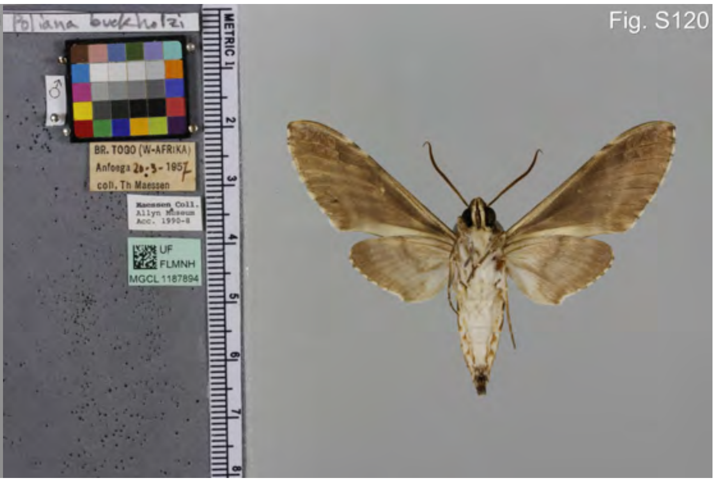

Poliana_buckholzi_MGCL_1187894_V.JPG

Habitus images of MGCL voucher specimens

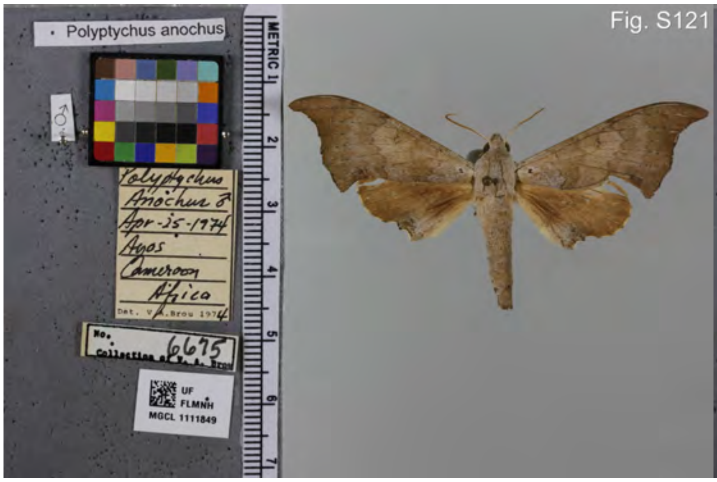

Polyptychus_anochus_MGCL_1111849_D.JPG

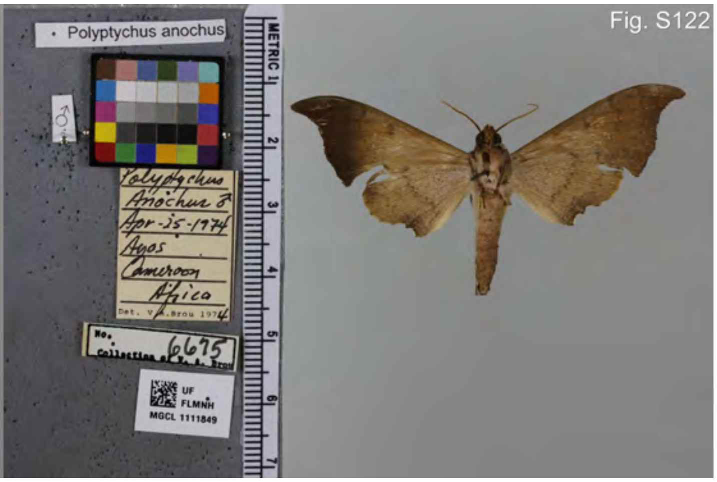

Polyptychus_anochus_MGCL_1111849_V.JPG

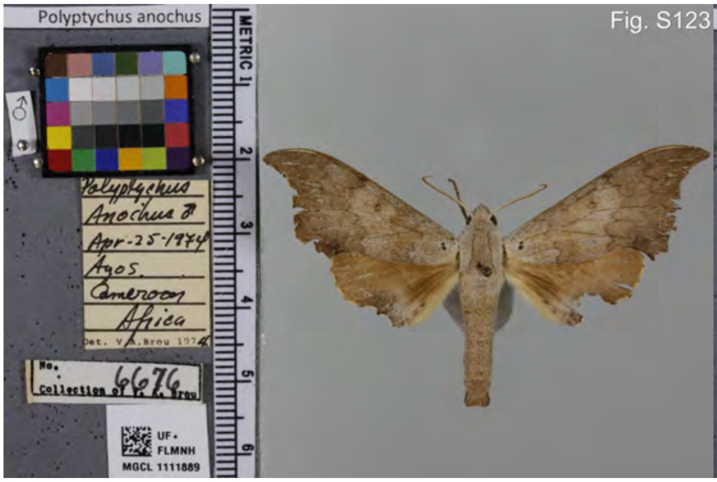

Polyptychus_anochus_MGCL_1111889_D.JPG

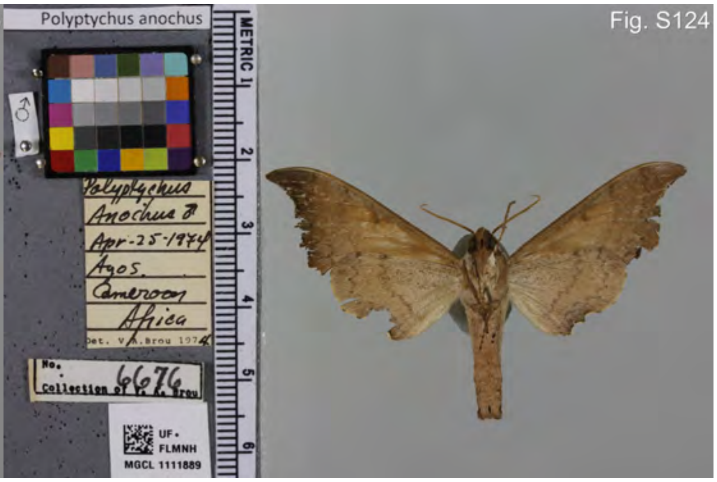

Polyptychus_anochus_MGCL_1111889_V.JPG

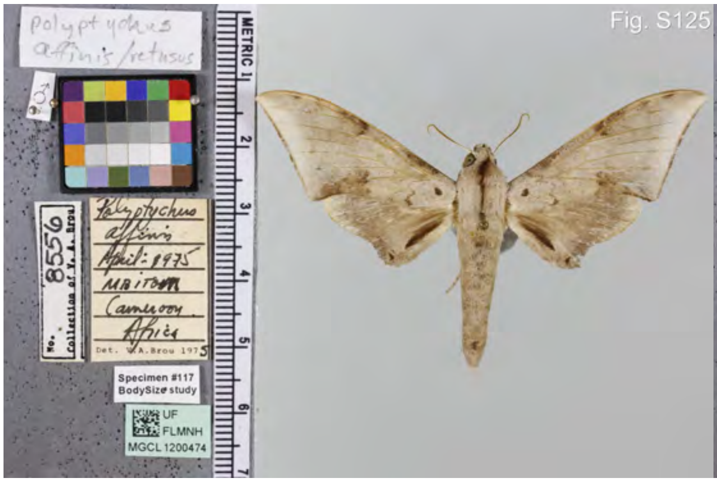

Polyptychus_retusus_MGCL_1200474_D.JPG

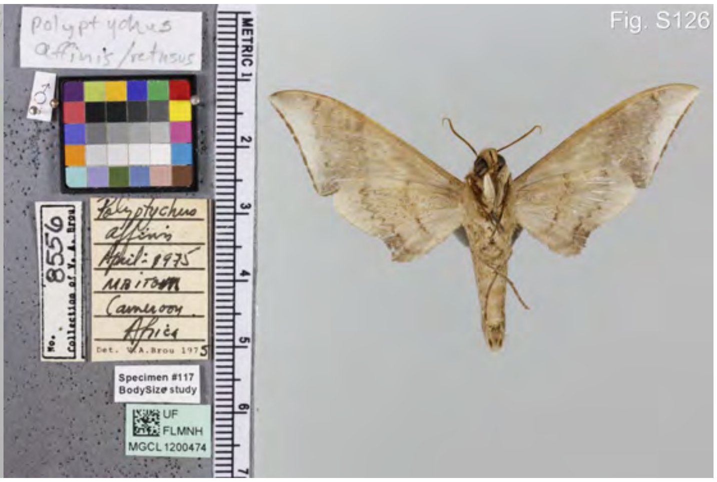

Polyptychus_retusus_MGCL_1200474_V.JPG

Habitus images of MGCL voucher specimens

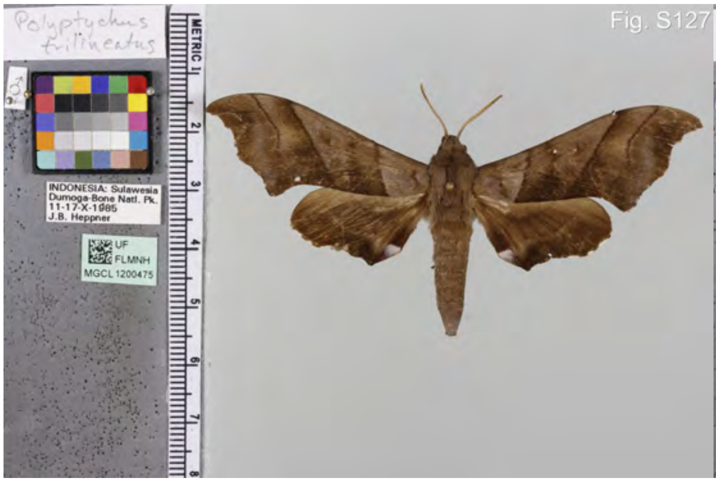

Polyptychus_trilineatus_MGCL_1200475_D.JPG

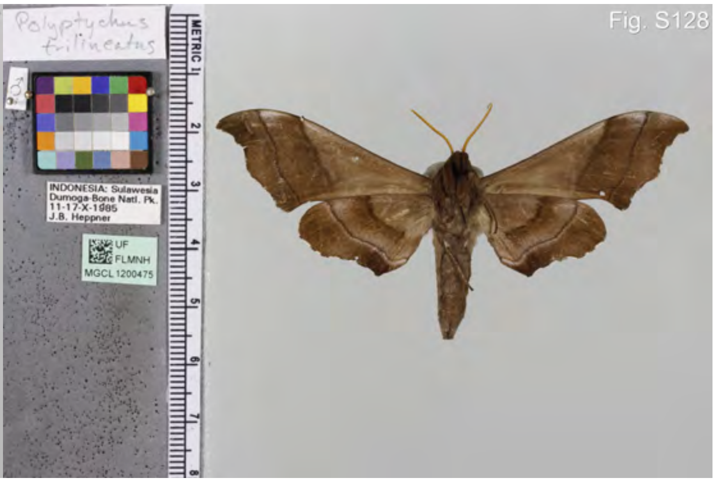

Polyptychus_trilineatus_MGCL_1200475_V.JPG

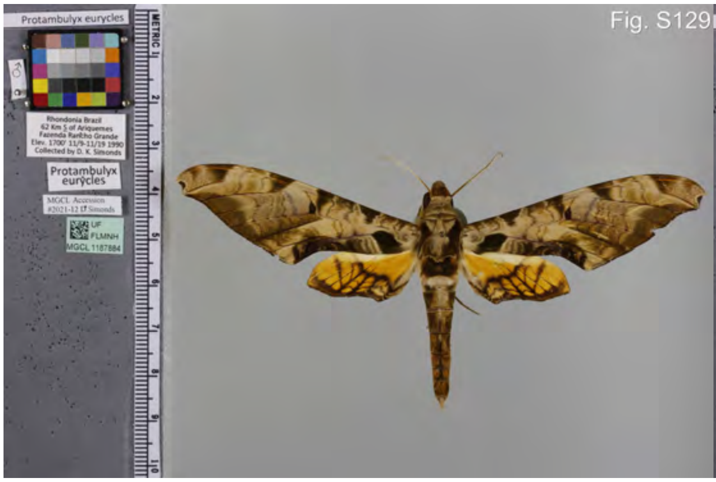

Protambulyx_eurycles_MGCL_1187884_D.JPG

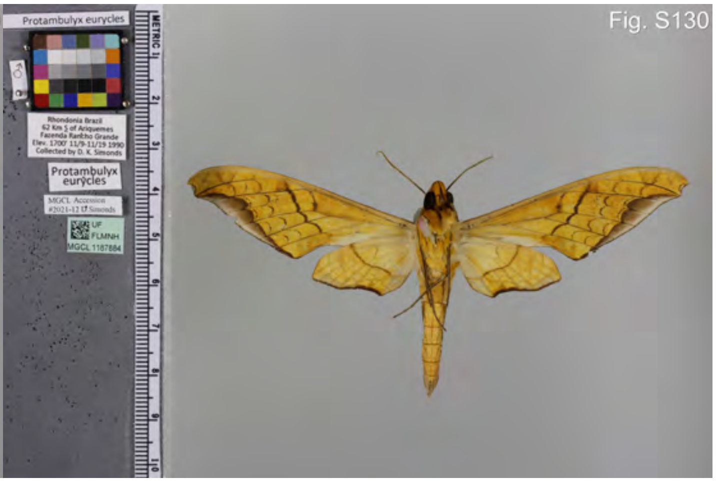

Protambulyx_eurycles_MGCL_1187884_V.JPG

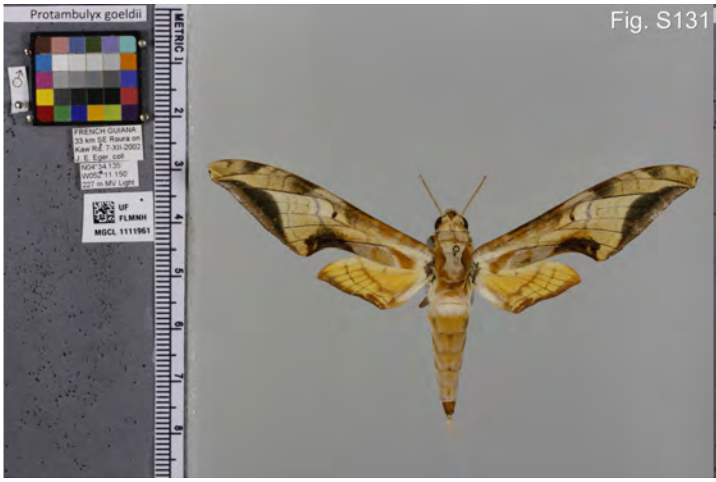

Protambulyx_goeldii_MGCL_1111961_D.JPG

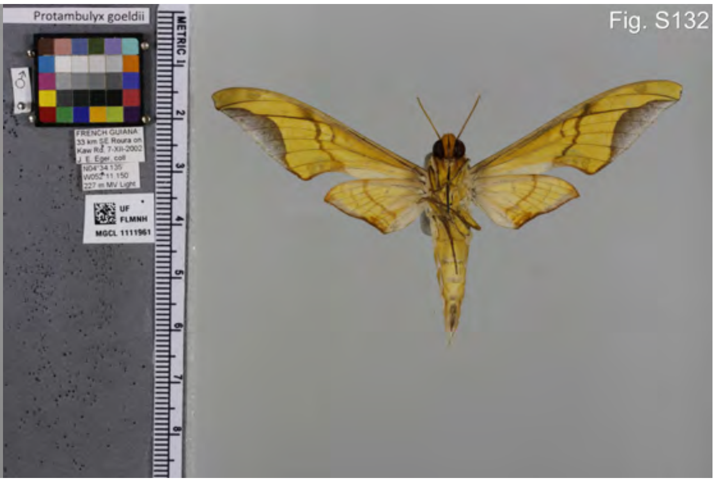

Protambulyx_goeldii_MGCL_1111961_V.JPG

Habitus images of MGCL voucher specimens

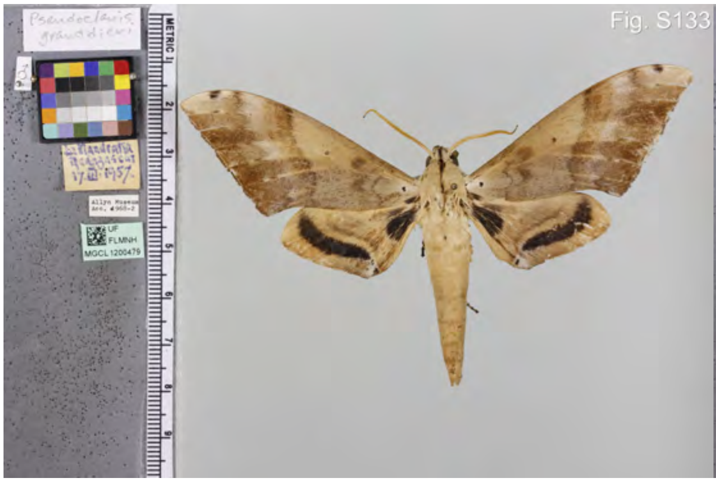

Pseudoclanis_grandidieri_MGCL_1200479_D.JPG

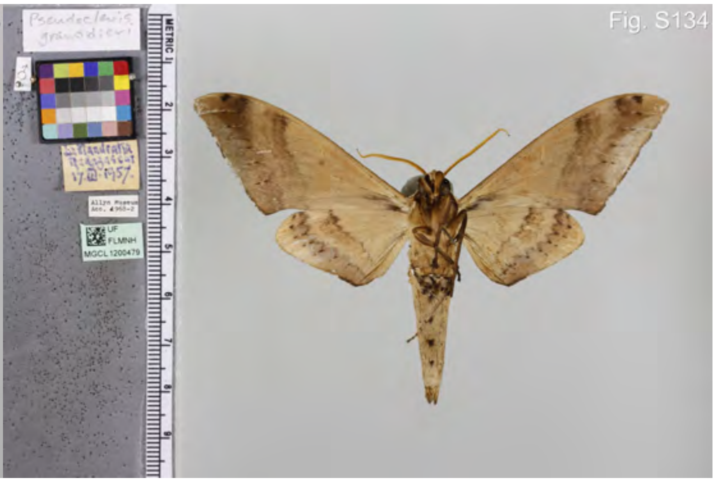

Pseudoclanis_grandidieri_MGCL_1200479_V.JPG

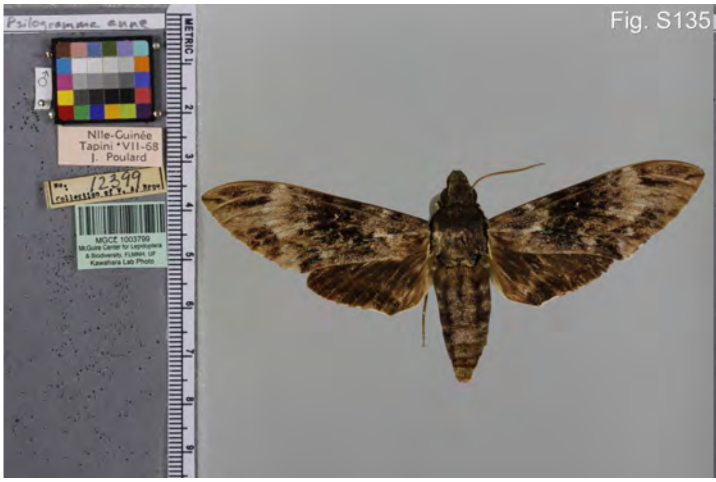

Psilogramma_anne_MGCL_1003799_D.JPG

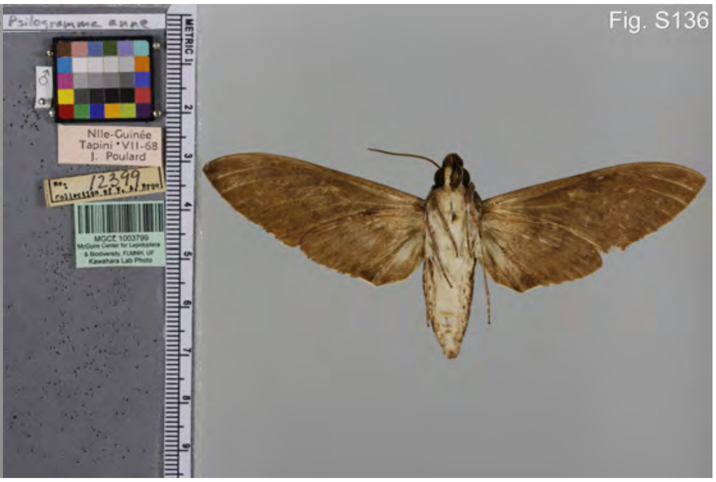

Psilogramma_anne_MGCL_1003799_V.JPG

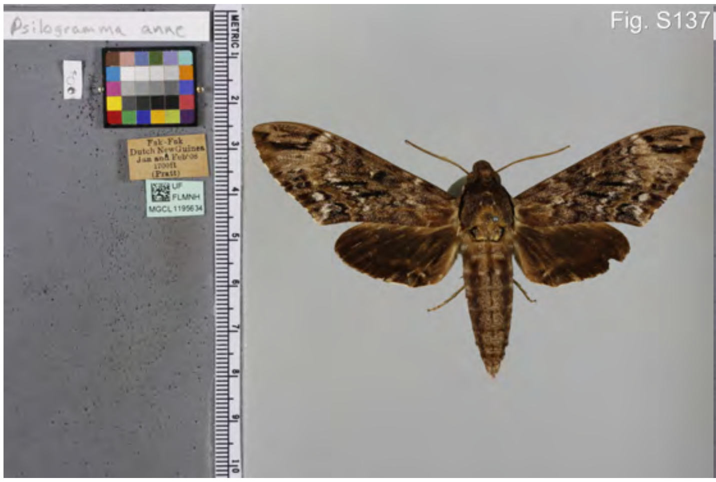

Psilogramma_anne_MGCL_1195634_D.JPG

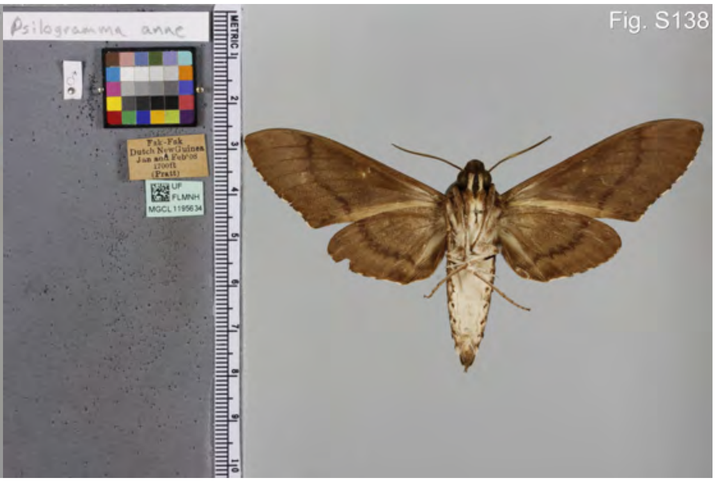

Psilogramma_anne_MGCL_1195634_V.JPG

Habitus images of MGCL voucher specimens

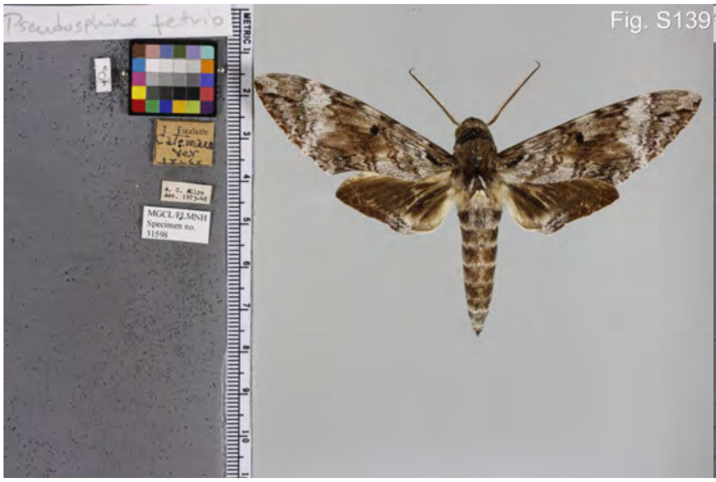

Fig. S139

Pseudosphinx_tetrio_MGCL_31598_D.JPG

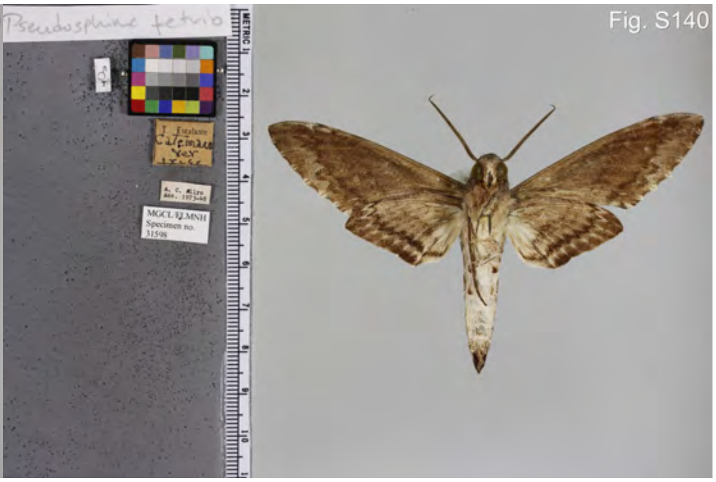

Fig. S140

Pseudosphinx_tetrio_MGCL_31598_V.JPG

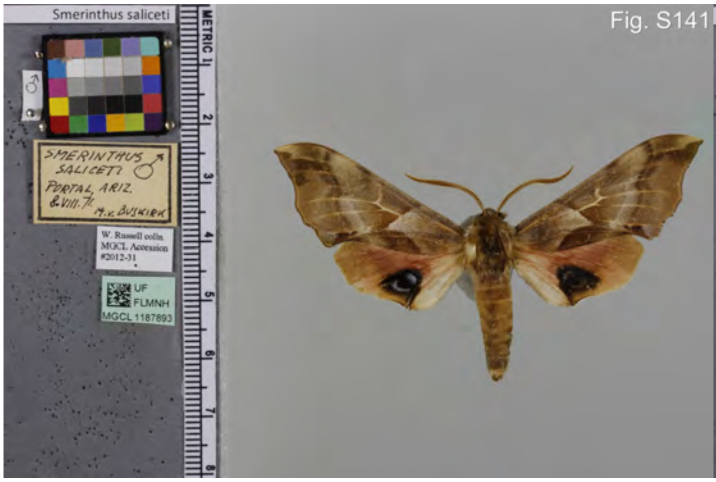

Fig. S141

Smerinthus_saliceti_MGCL_1187893_D.JPG

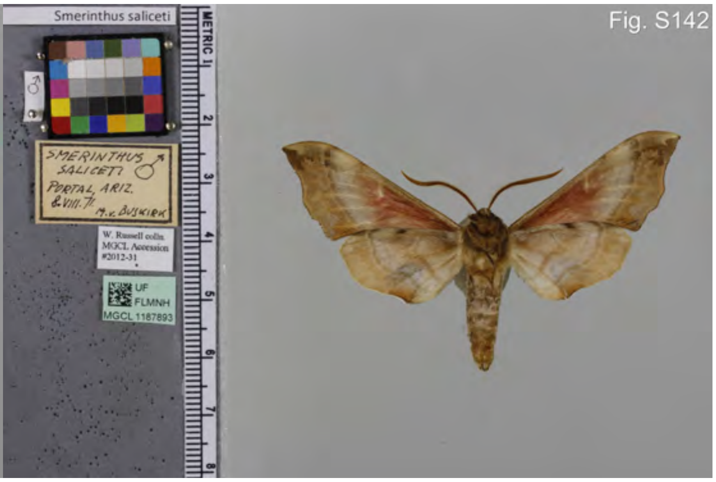

Fig. S142

Smerinthus_saliceti_MGCL_1187893_V.JPG

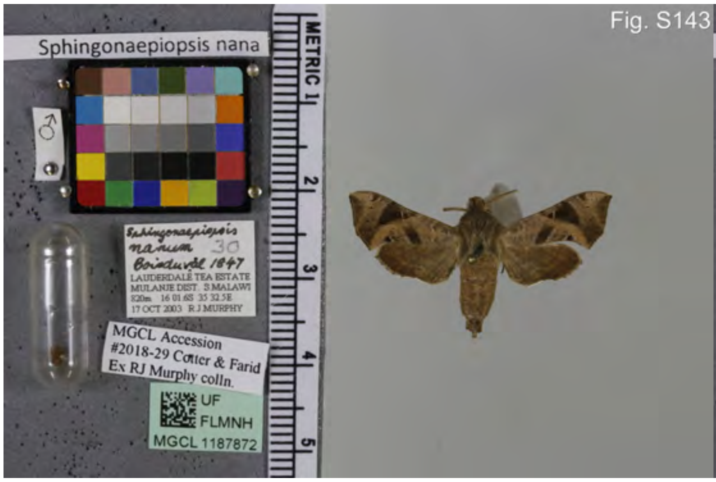

Fig. S143

Sphingonaepiopsis_nana_MGCL_1187872_D.JPG

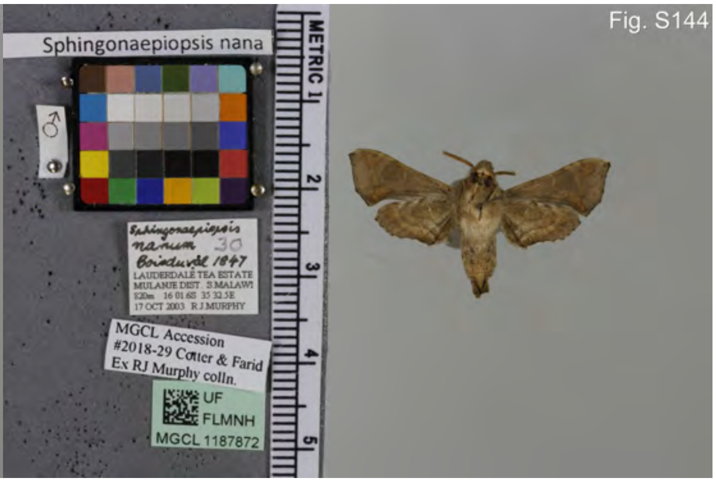

Fig. S144

Sphingonaepiopsis_nana_MGCL_1187872_V.JPG

Habitus images of MGCL voucher specimens

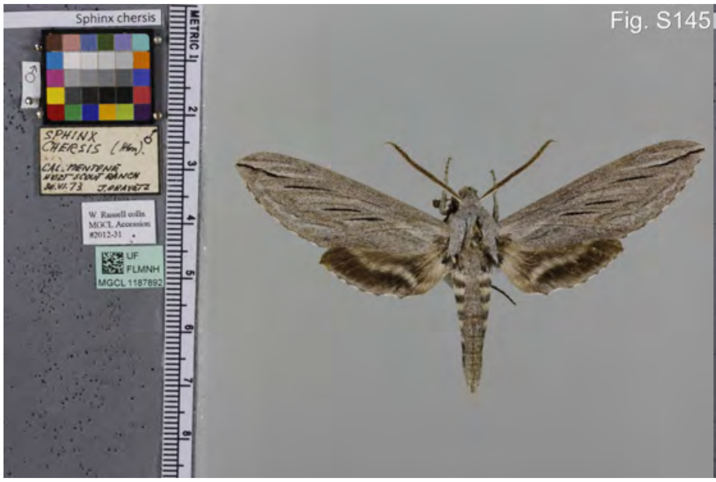

Sphinx_chersis_MGCL_1187892_D.JPG

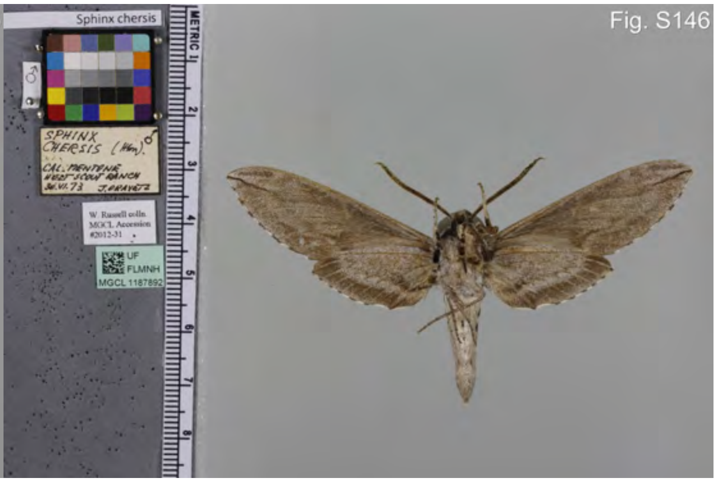

Sphinx_chersis_MGCL_1187892_V.JPG

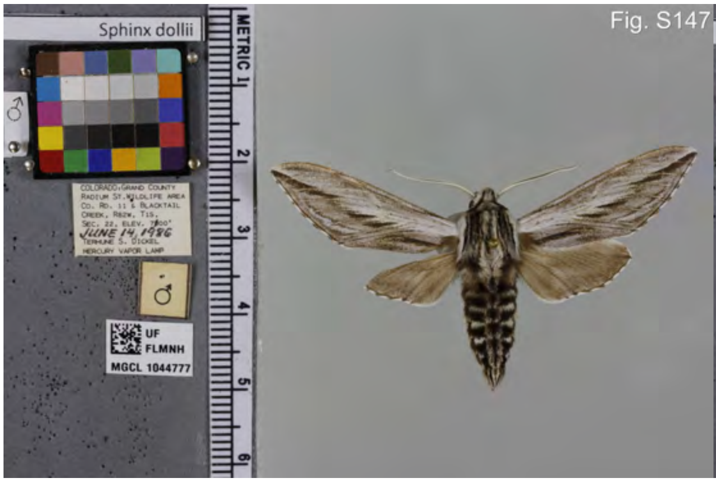

Sphinx_dollii_MGCL_1044777_D.JPG

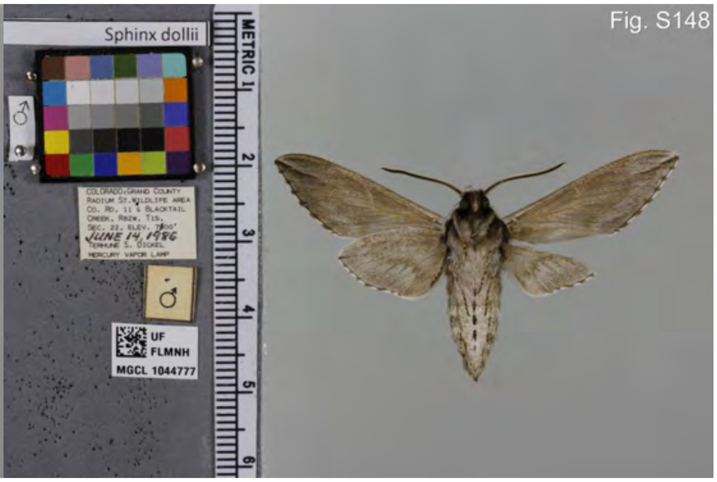

Sphinx_dollii_MGCL_1044777_V.JPG

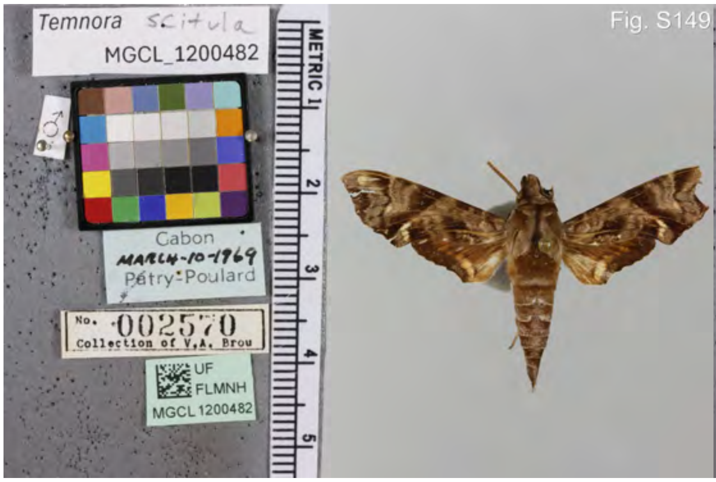

Temnora_scitula_MGCL_1200482_D.JPG

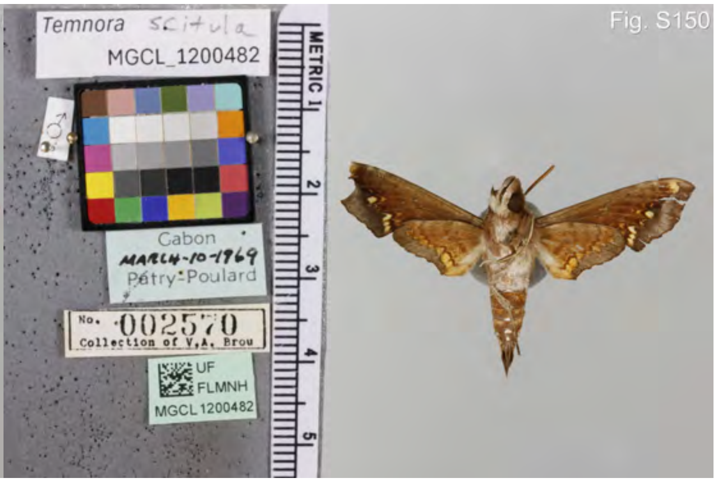

Temnora_scitula_MGCL_1200482_V.JPG

Habitus images of MGCL voucher specimens

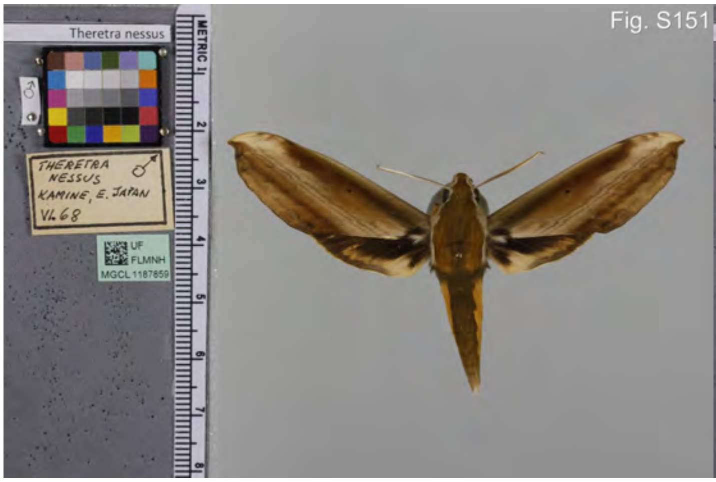

Theretra_nessus_MGCL_1187859_D.JPG

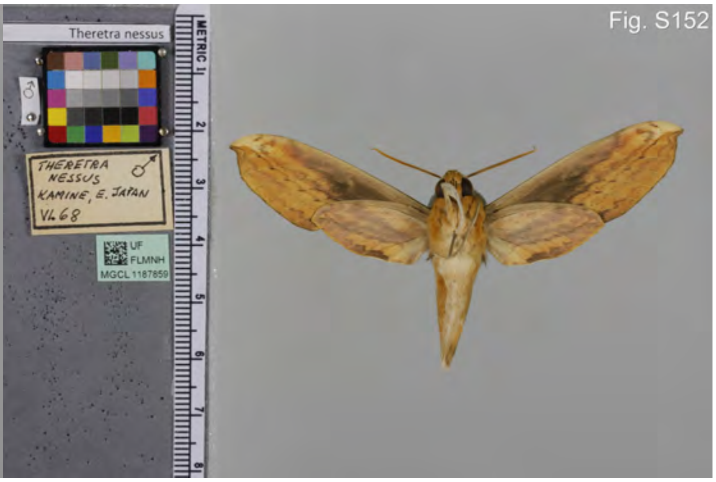

Theretra_nessus_MGCL_1187859_V.JPG

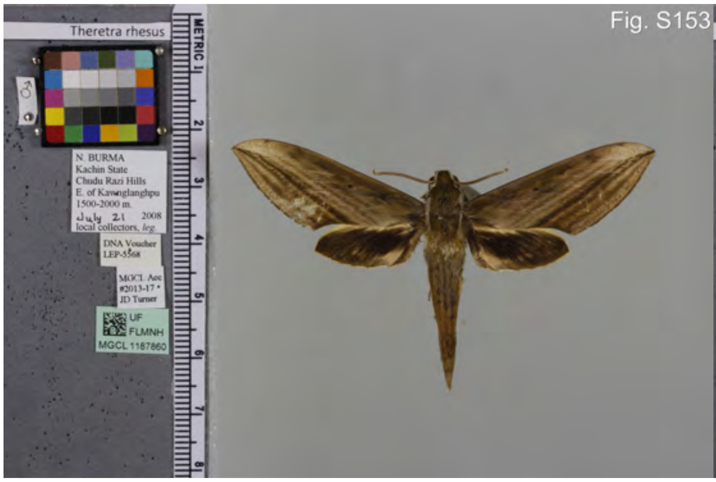

Theretra_rhesus_MGCL_1187860_D.JPG

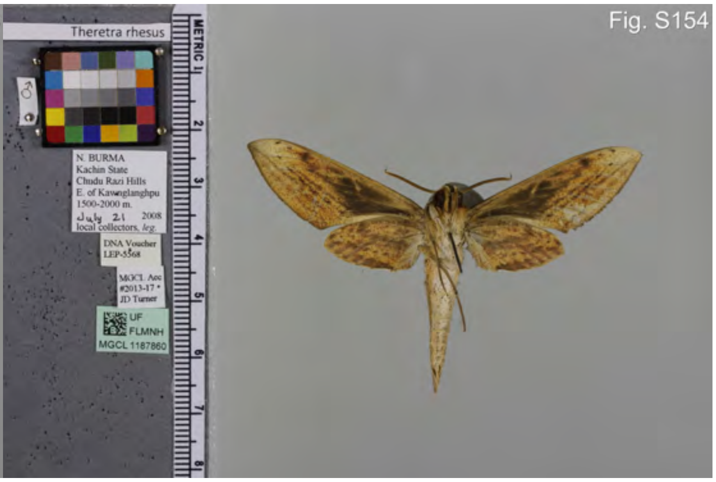

Theretra_rhesus_MGCL_1187860_V.JPG

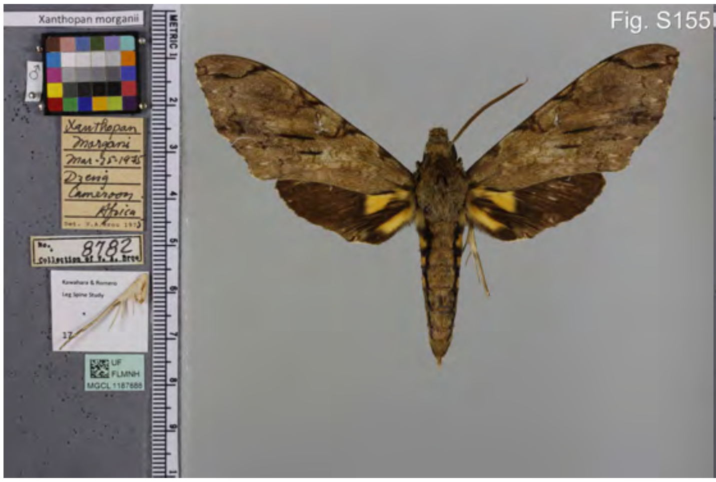

Xanthopan_morganii_MGCL_1187888_D.JPG

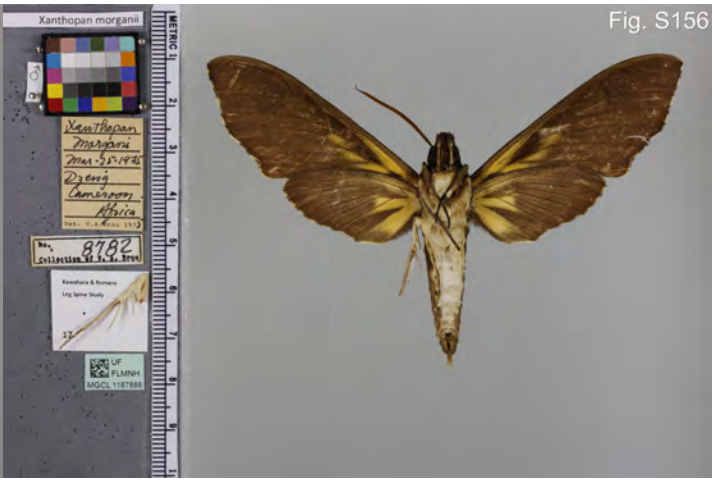

Xanthopan_morganii_MGCL_1187888_V.JPG

Habitus images of MGCL voucher specimens

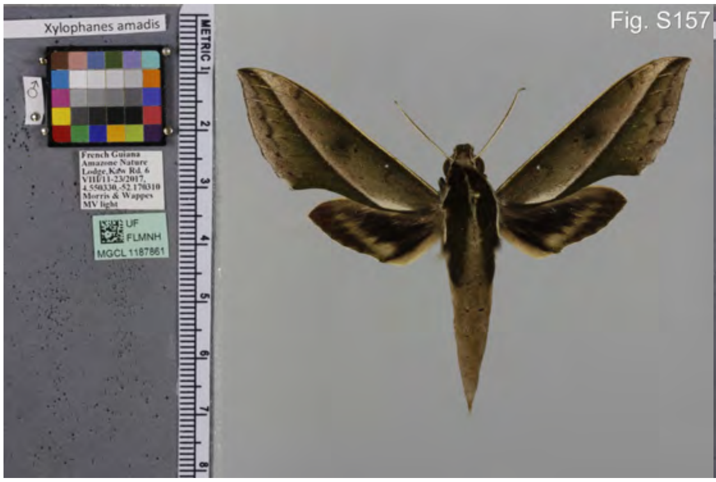

Fig. S157

Xylophanes_amadis_MGCL_1187861_D.JPG

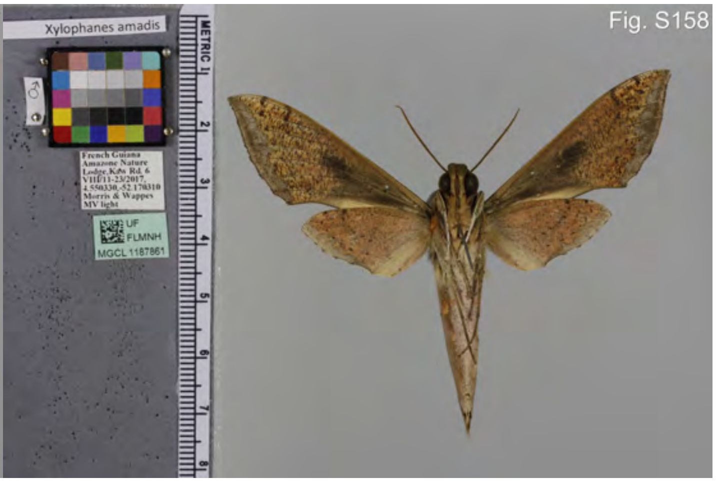

Fig. S158

Xylophanes_amadis_MGCL_1187861_V.JPG

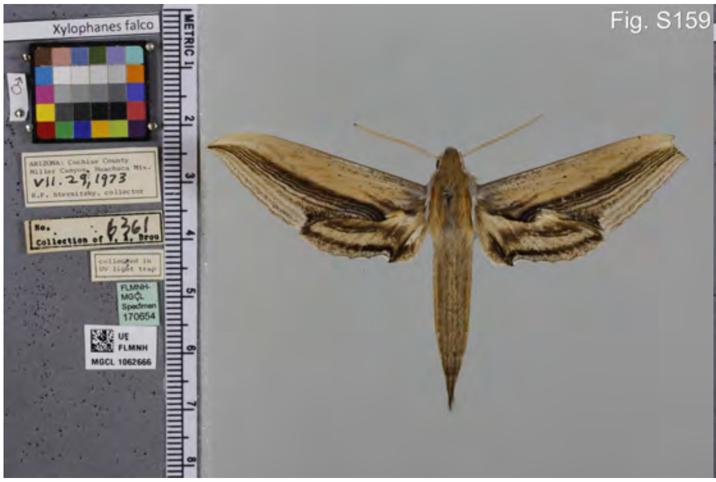

Fig. S159

Xylophanes_falco_MGCL_1062666_D.JPG

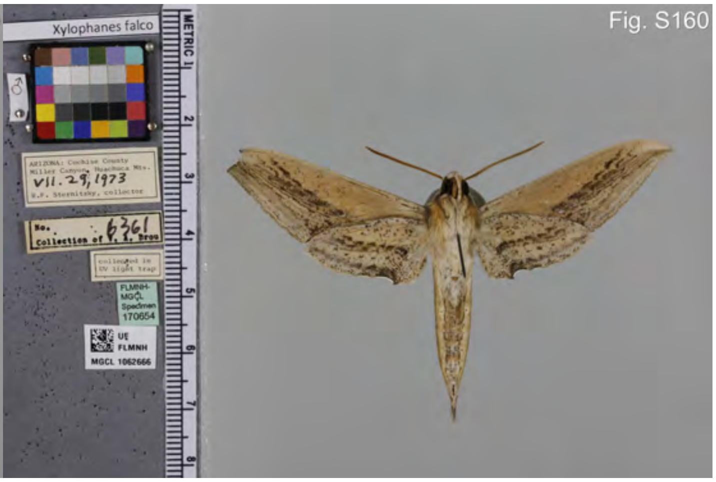

Fig. S160

Xylophanes_falco_MGCL_1062666_V.JPG

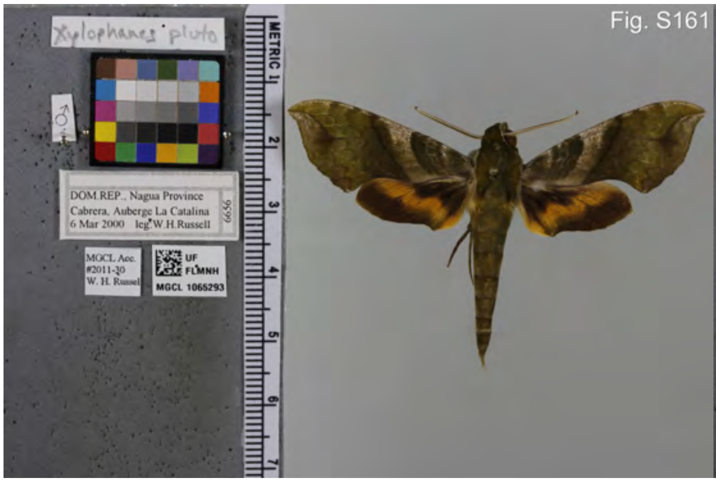

Fig. S161

Xylophanes_pluto_MGCL_1065293_D.JPG

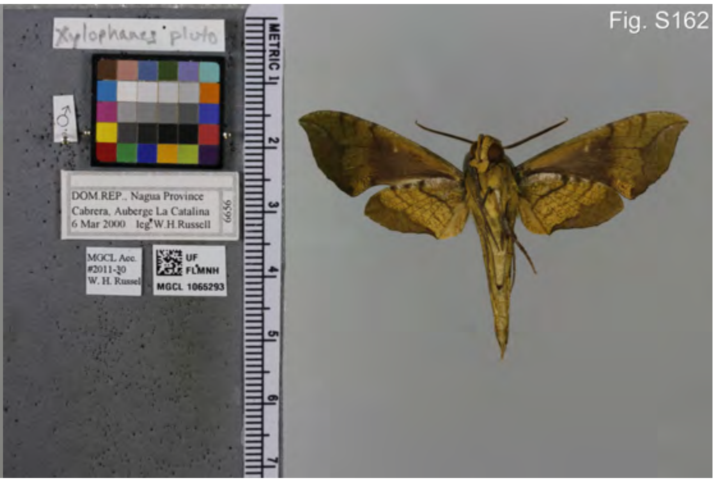

Fig. S162

Xylophanes_pluto_MGCL_1065293_V.JPG

Habitus images of MGCL voucher specimens

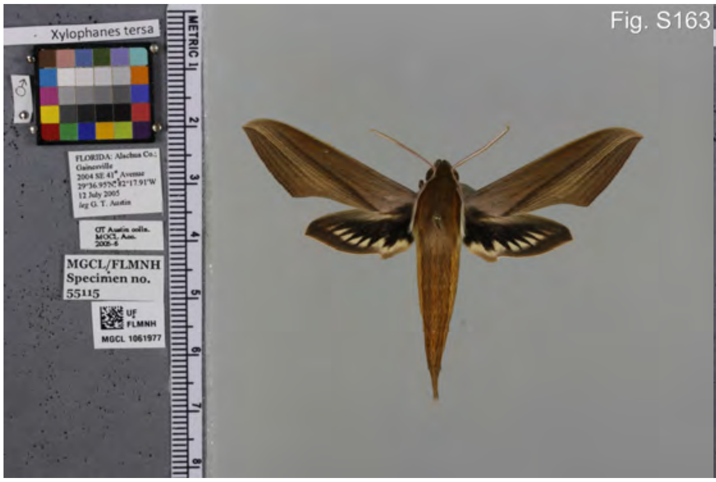

Fig. S163

Xylophanes_tersa_MGCL_1061977_D.JPG

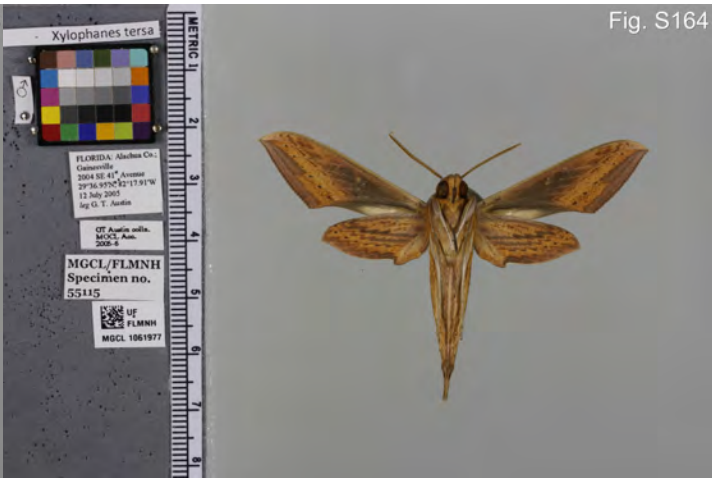

Fig. S164

Xylophanes_tersa_MGCL_1061977_V.JPG

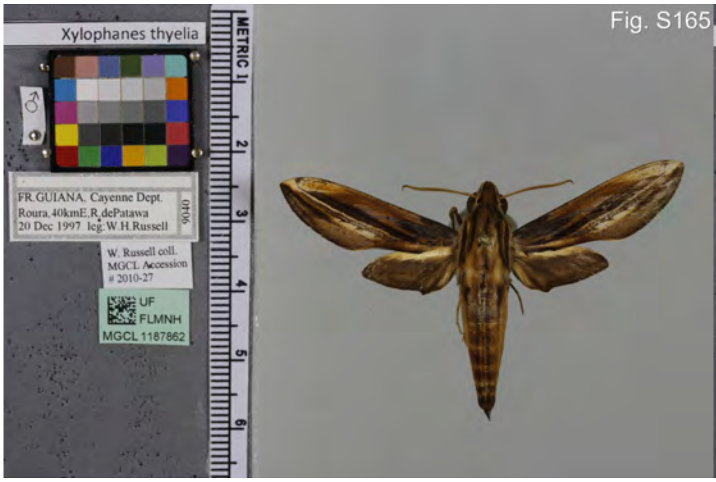

Fig. S165

Xylophanes_thyelia_MGCL_1187862_D.JPG

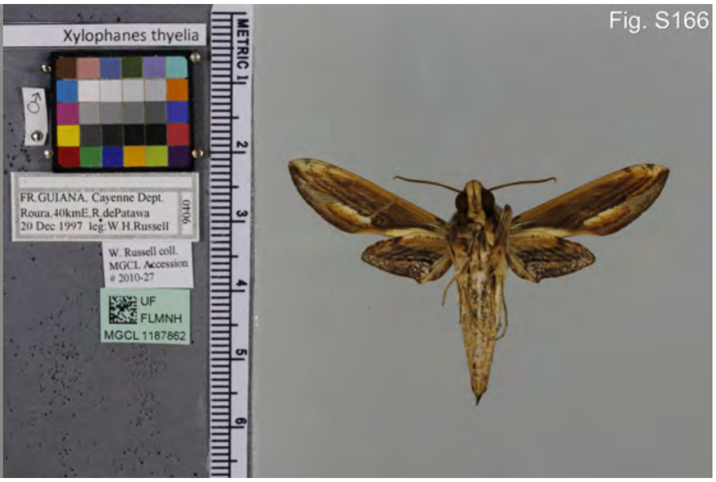

Fig. S166

Xylophanes_thyelia_MGCL_1187862_V.JPG

Adhemarius gannascus

scale-on-scale

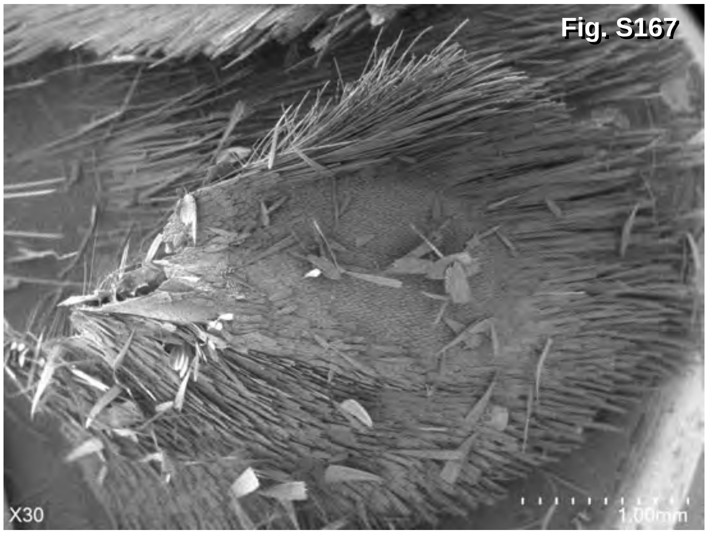

GP0025_Adhemarius_gannascus_LeftValve_30x.tif

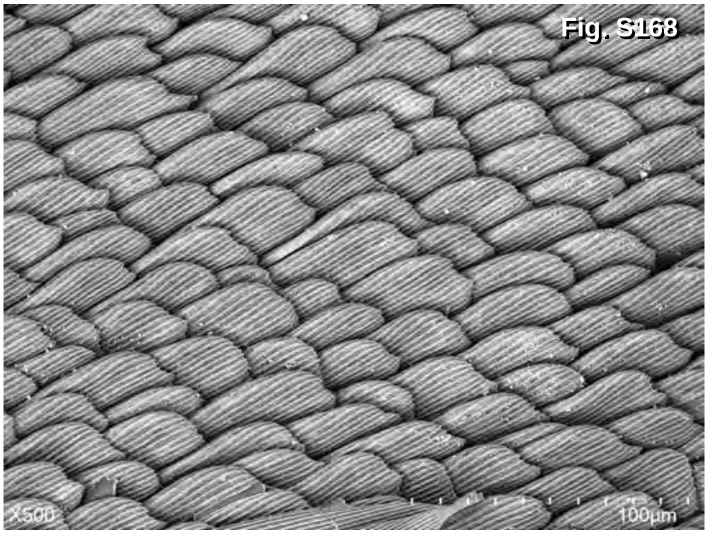

GP0025_Adhemarius_gannascus_LeftValve_500x.tif

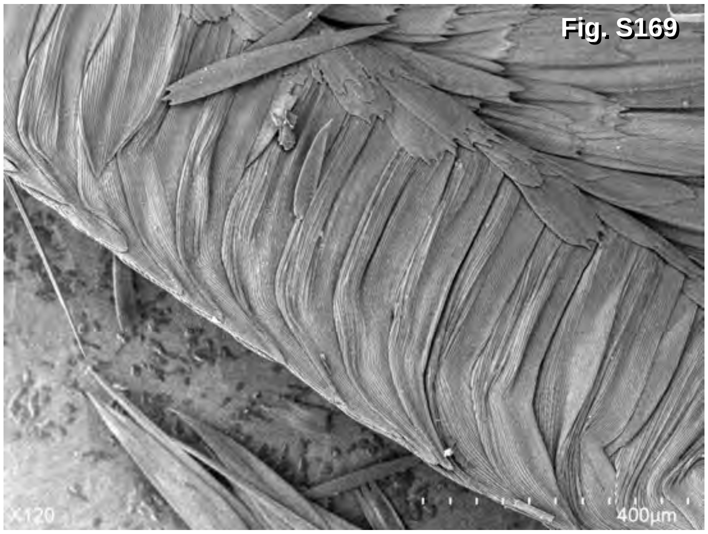

GP0025_Adhemarius_gannascus_T8scales_120x.tif

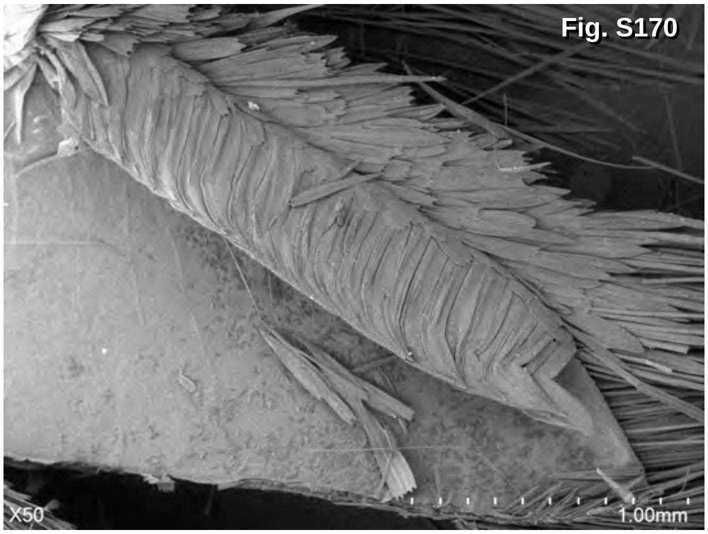

GP0025_Adhemarius_gannascus_T8scales_50x.tif

Ambulyx moorei

scale-on-scale

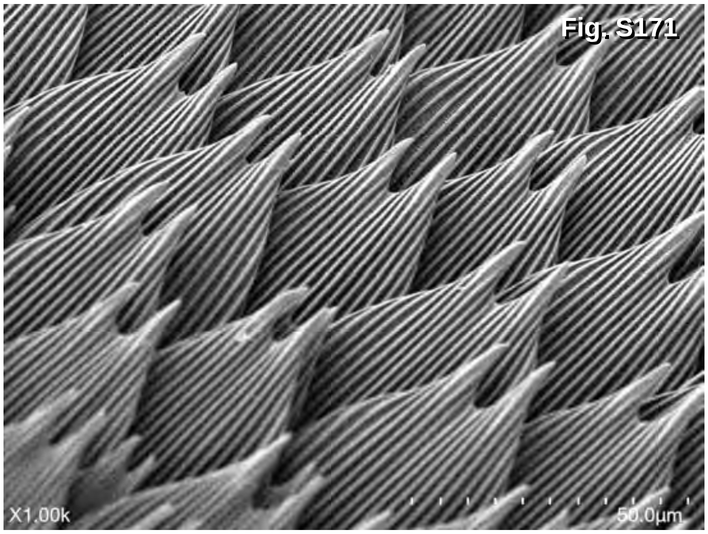

GP0061_Ambulyx_moorei_LeftValve_1000x.tif

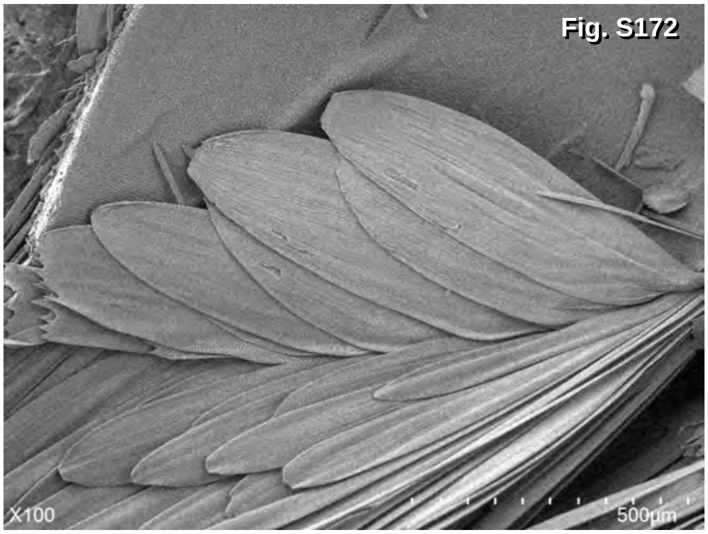

GP0061_Ambulyx_moorei_T8scales_100x.tif

Ambulyx pryeri

scale-on-scale

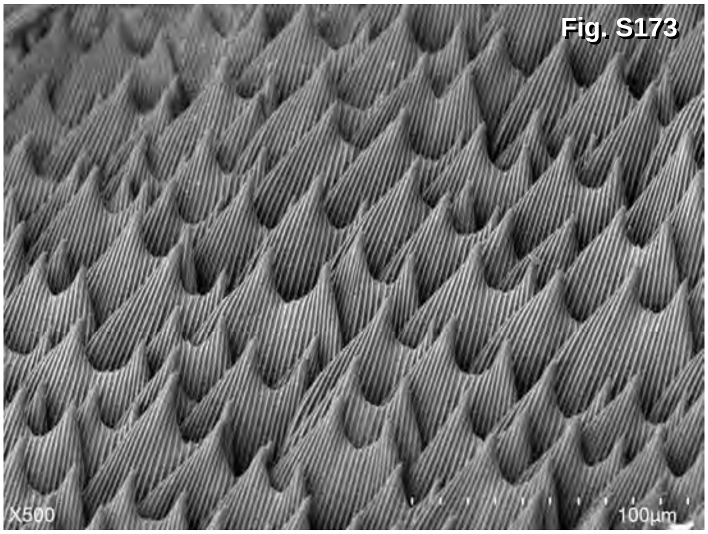

GP0021_Ambulyx_pryeri_LeftValve_500x.tif

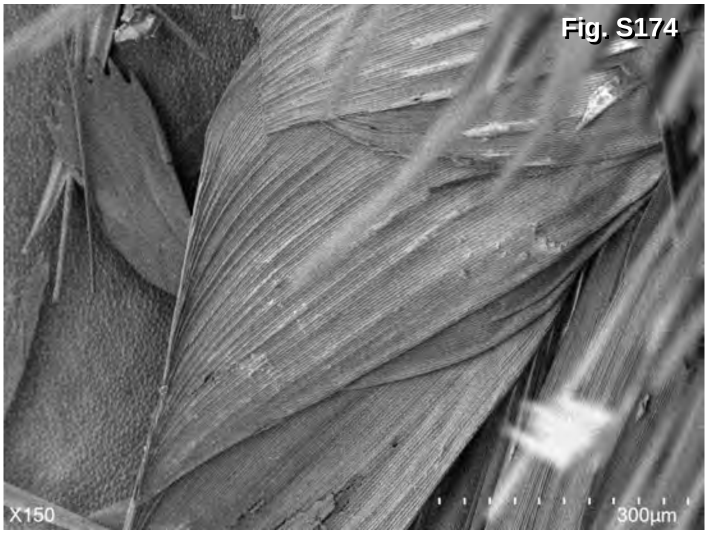

GP0021_Ambulyx_pryeri_T8scales_150x.tif

Amplypterus panopus

scale-on-scale

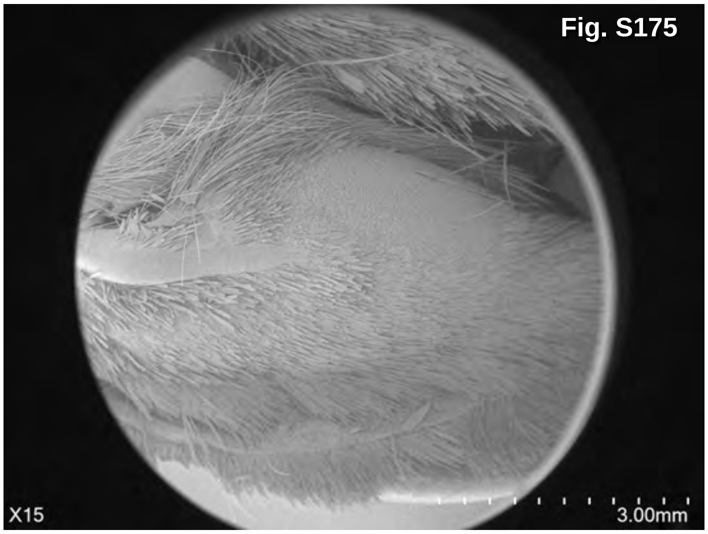

GP0022_Amplypterus_panopus_LeftValve_15x.tif

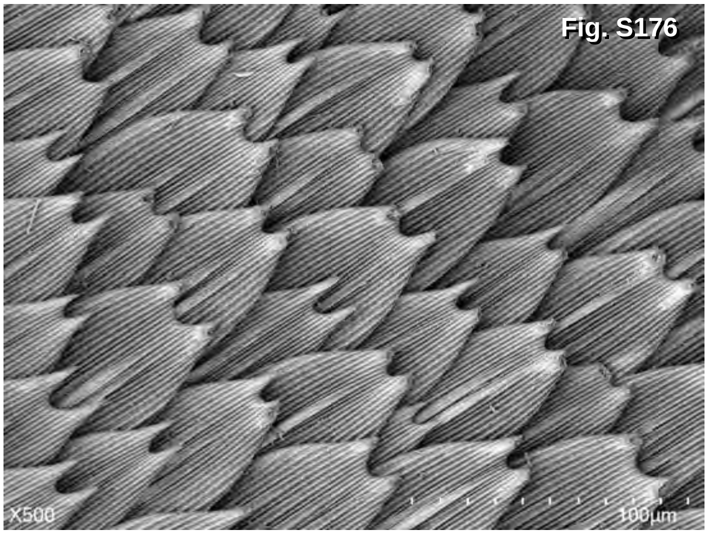

GP0022_Amplypterus_panopus_LeftValve_500x.tif

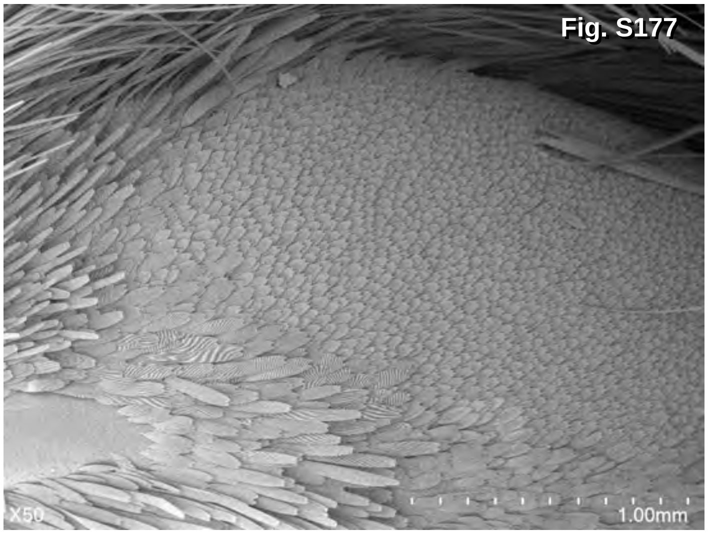

GP0022_Amplypterus_panopus_LeftValve_50x.tif

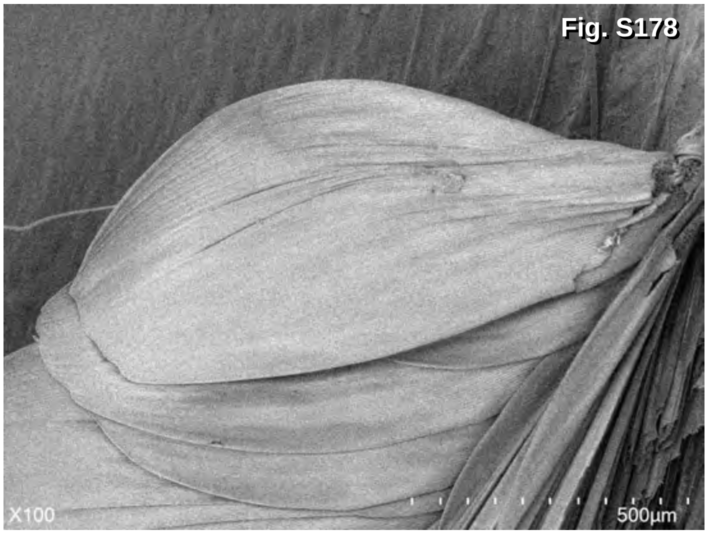

GP0022_Amplypterus_panopus_T8_100x.tif

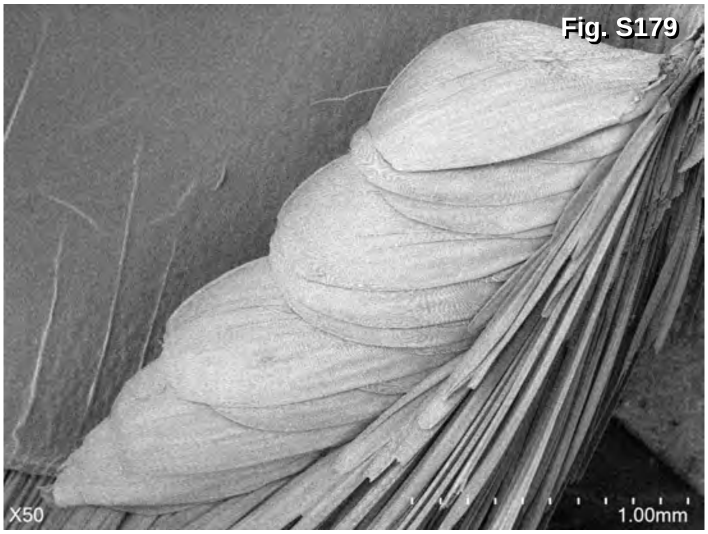

GP0022_Amplypterus_panopus_T8_50x.tif

Meganoton nyctiphanes

scale-on-scale

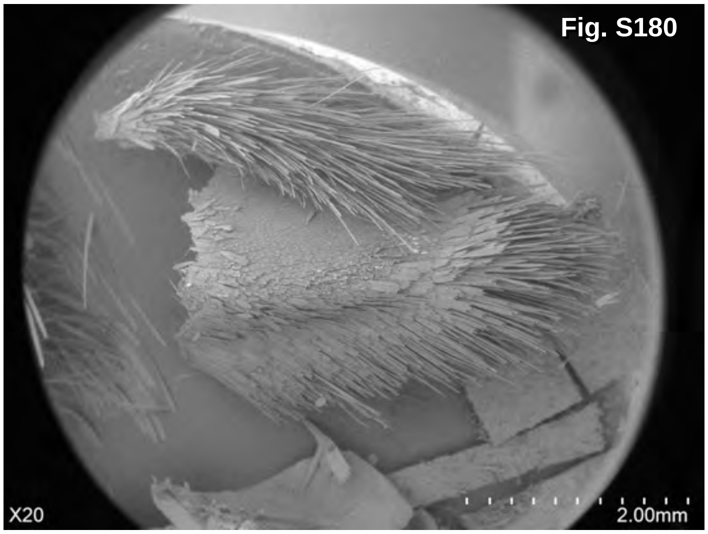

GP0043_Meganoton_nyctiphanes_LeftValve_20x.tif

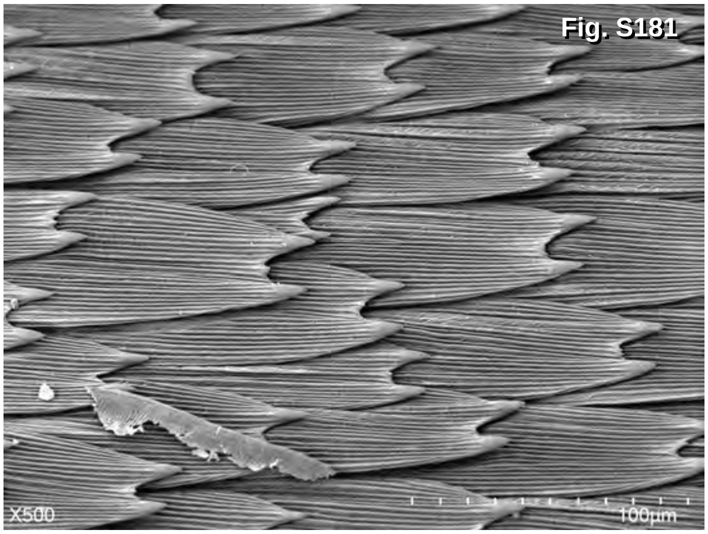

GP0043_Meganoton_nyctiphanes_LeftValve_500x.tif

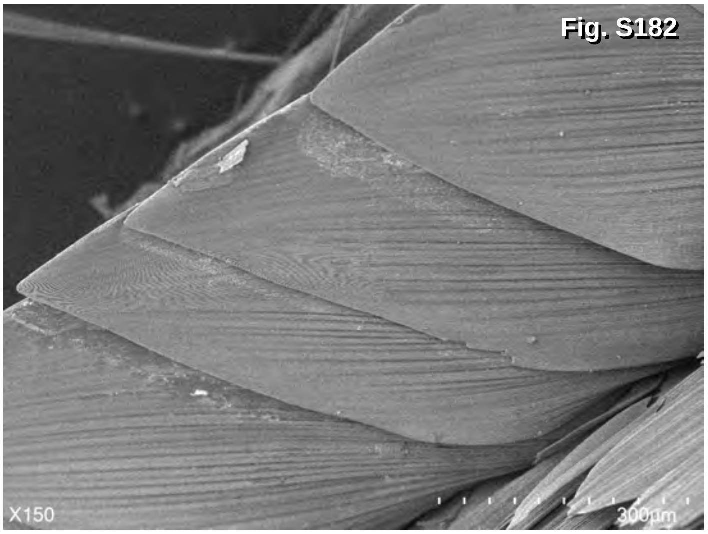

GP0043_Meganoton_nyctiphanes_T8scales_150x.tif

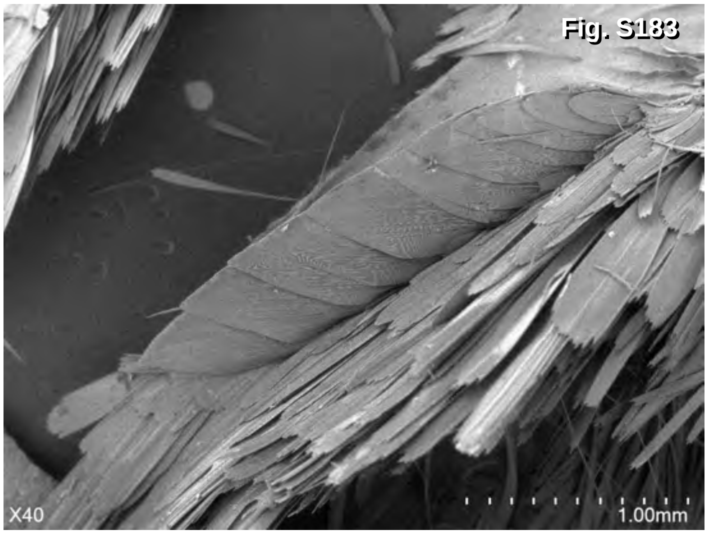

GP0043_Meganoton_nyctiphanes_T8scales_40x.tif

Polyptychus anochus

scale-on-scale

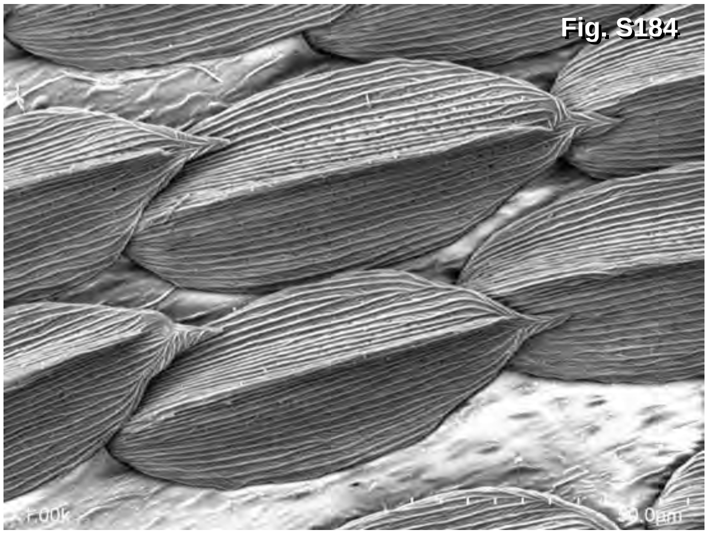

GP0028_Polyptychus_anochus_LeftValve_1000x.tif

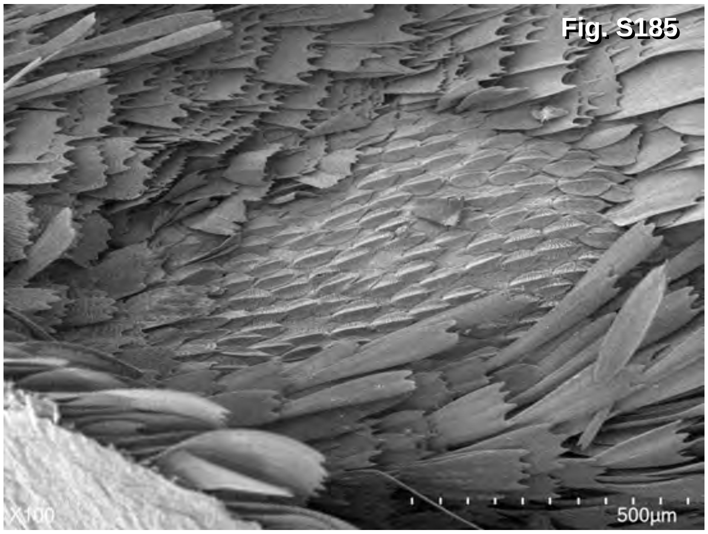

GP0028_Polyptychus_anochus_LeftValve_100x.tif

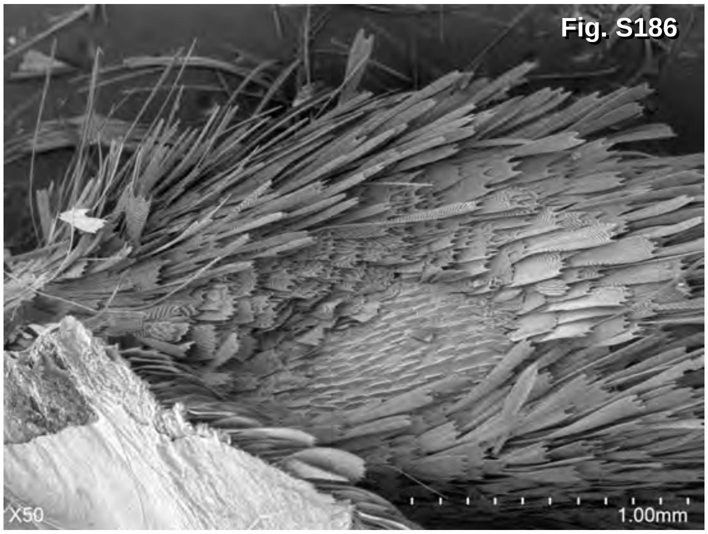

GP0028_Polyptychus_anochus_LeftValve_50x.tif

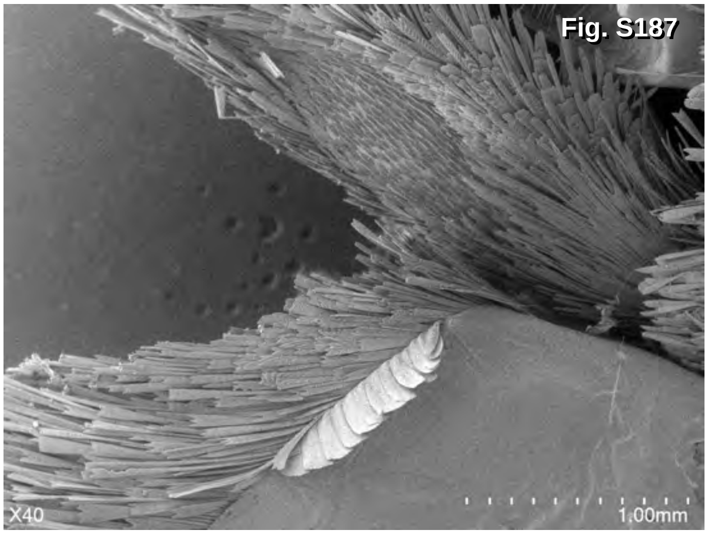

GP0071_Polyptychus_anochus_RightValve_T8_x40.tif

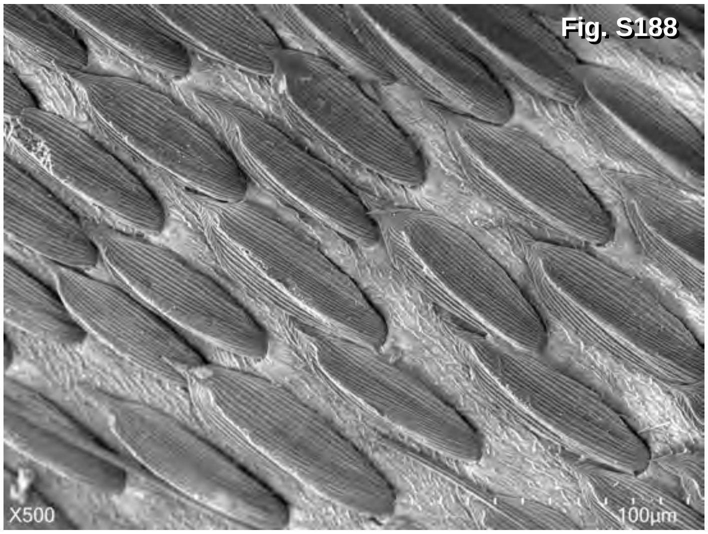

GP0071_Polyptychus_anochus_RightValve_x500.tif

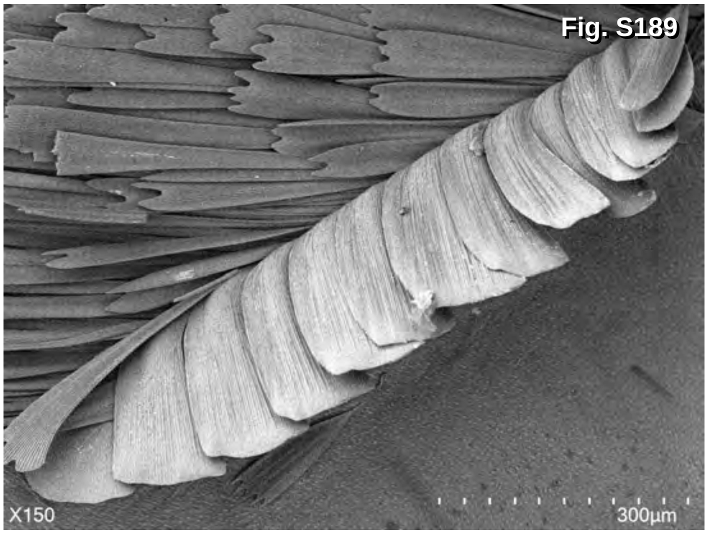

GP0071_Polyptychus_anochus_T8_x150.tif

Protambulyx eurycles

scale-on-scale

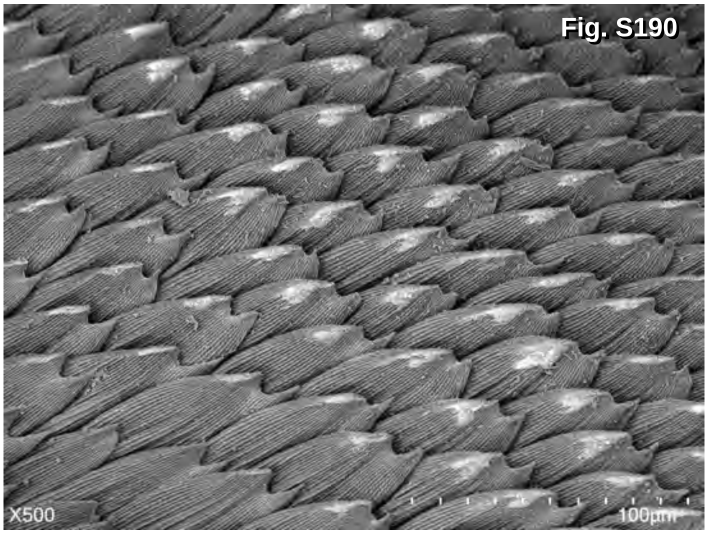

Fig. S190

GP0023_Protambulyx_eurycles_LeftValve_500x.tif

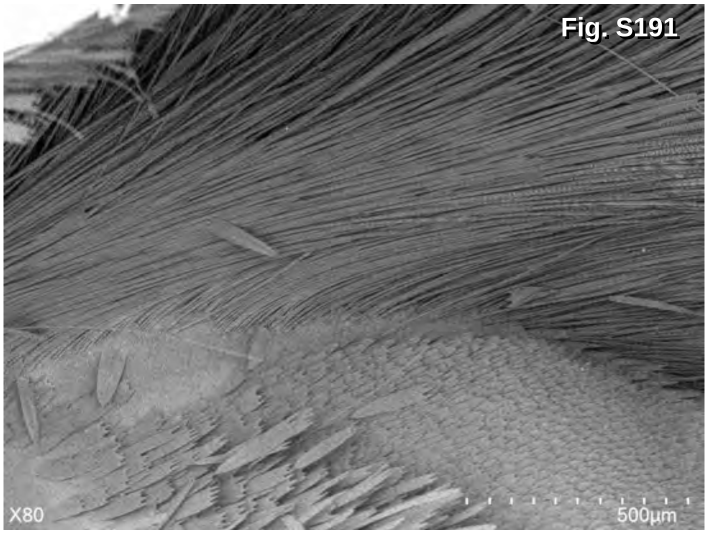

Fig. S191

GP0023_Protambulyx_eurycles_LeftValve_80x.tif

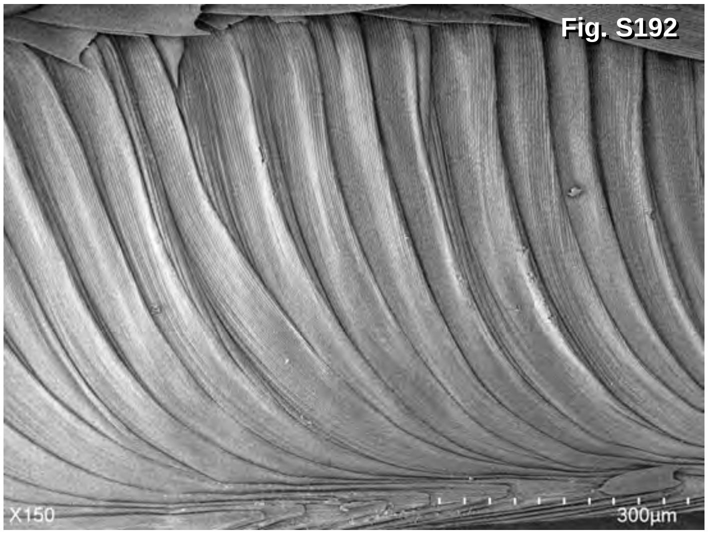

Fig. S192

GP0023_Protambulyx_eurycles_T8_150x.tif

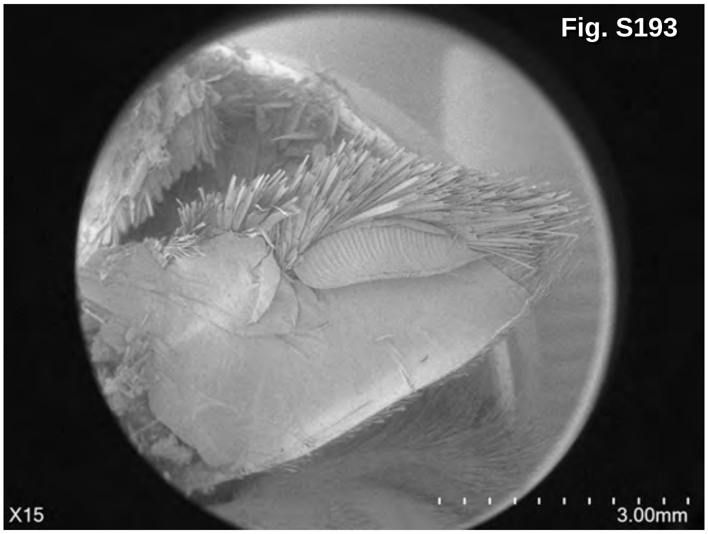

Fig. S193

GP0023_Protambulyx_eurycles_T8_15x.tif

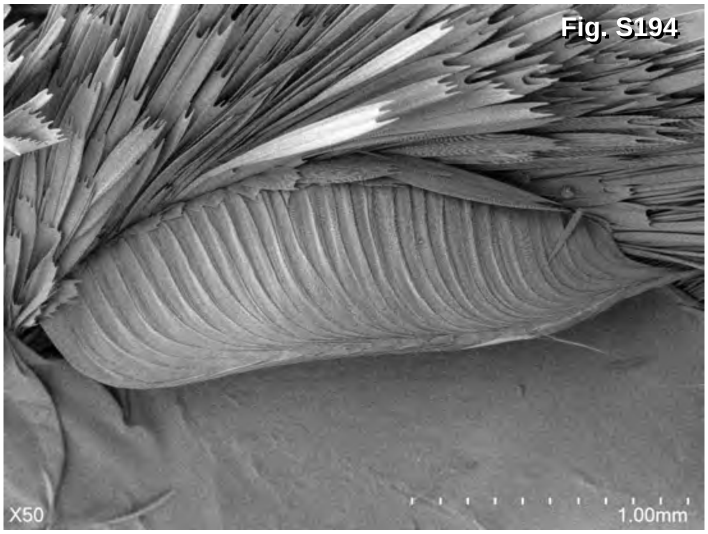

Fig. S194

GP0023_Protambulyx_eurycles_T8_50x.tif

Protambulyx goeldii

scale-on-scale

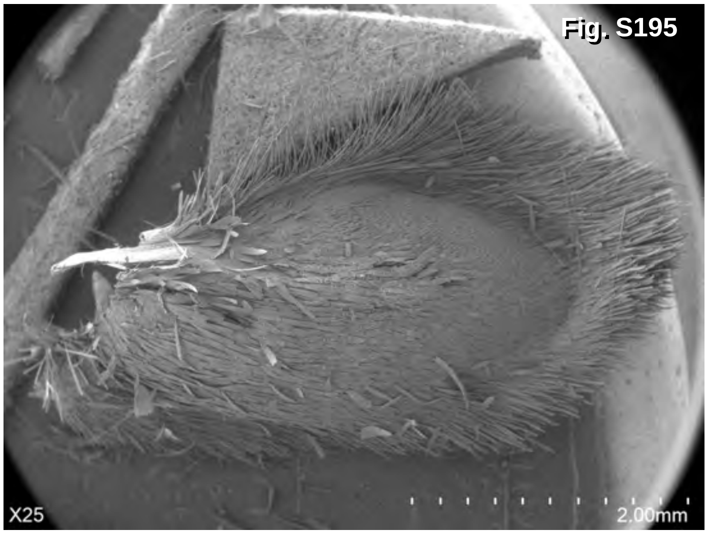

GP0026_Protambulyx_goeldii_25x.tif

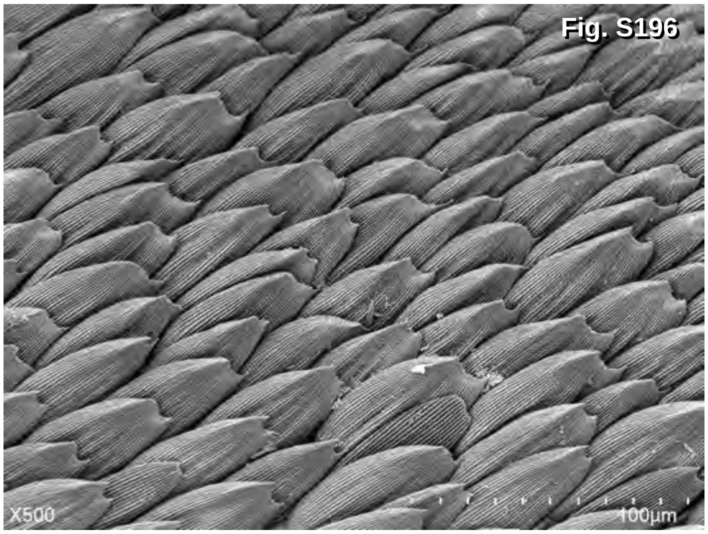

GP0026_Protambulyx_goeldii_LeftValve_500x.tif

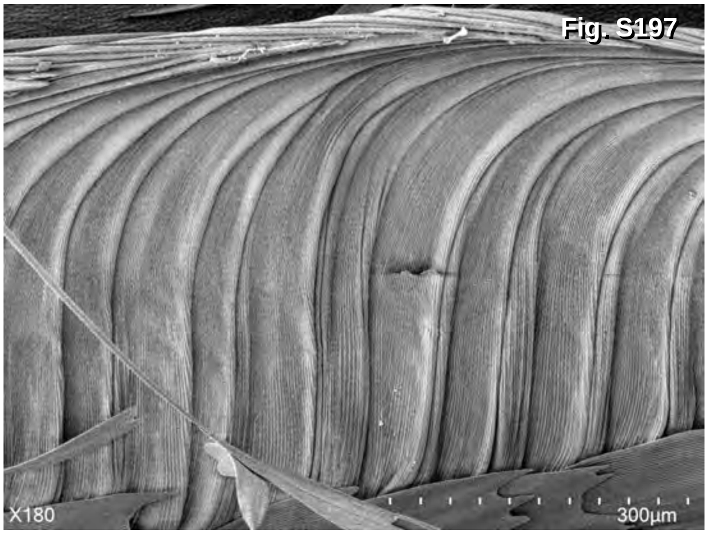

GP0026_Protambulyx_goeldii_T8scales_180x.tif

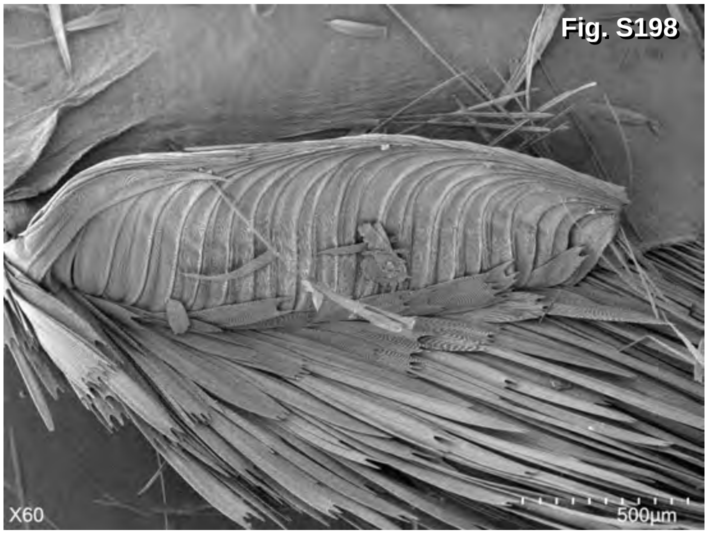

GP0026_Protambulyx_goeldii_T8scales_60x.tif

Agrius cingulata

scale-on-spine

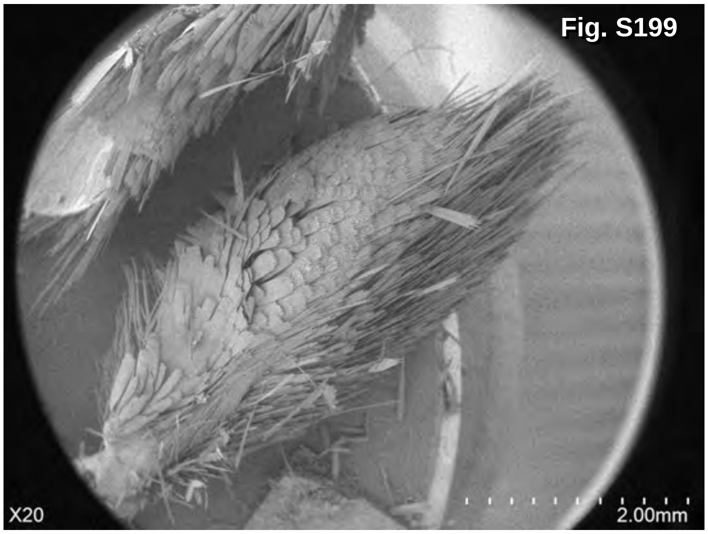

GP0049_Agrius_cingulata_LeftValve_20x.tif

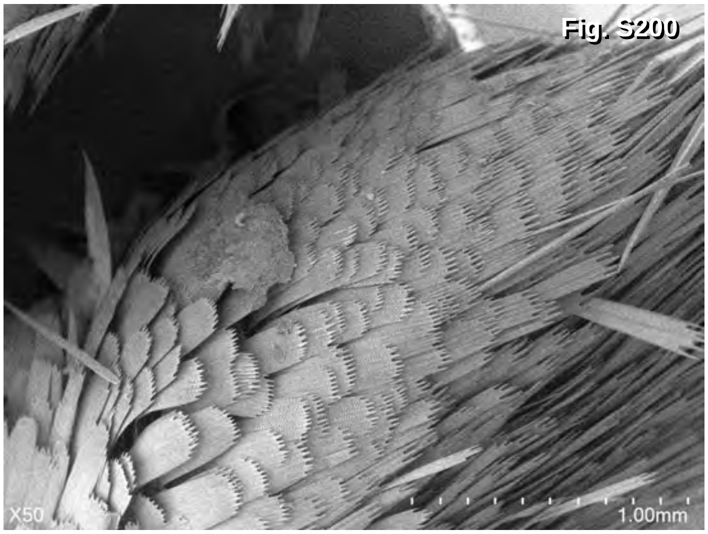

GP0049_Agrius_cingulata_LeftValve_50x.tif

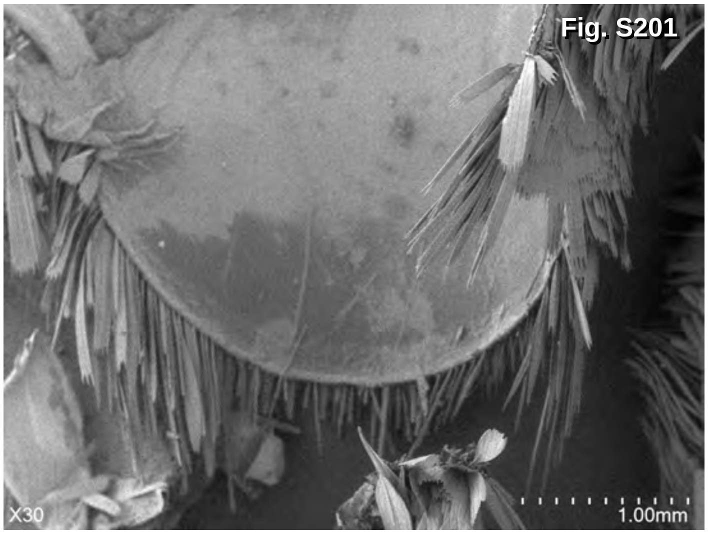

GP0049_Agrius_cingulata_T8_30x.tif

Agrius convolvuli

scale-on-spine

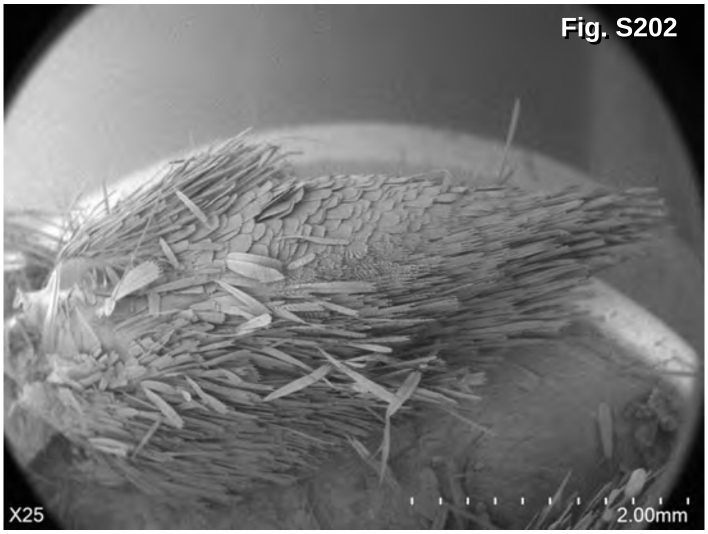

GP0046_Agrius_convolvuli_LeftValve_25x.tif

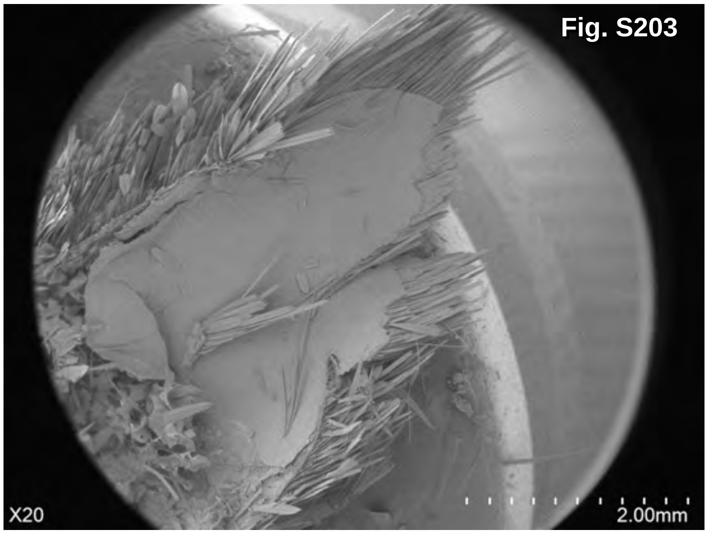

GP0046_Agrius_convolvuli_T8_20x.tif

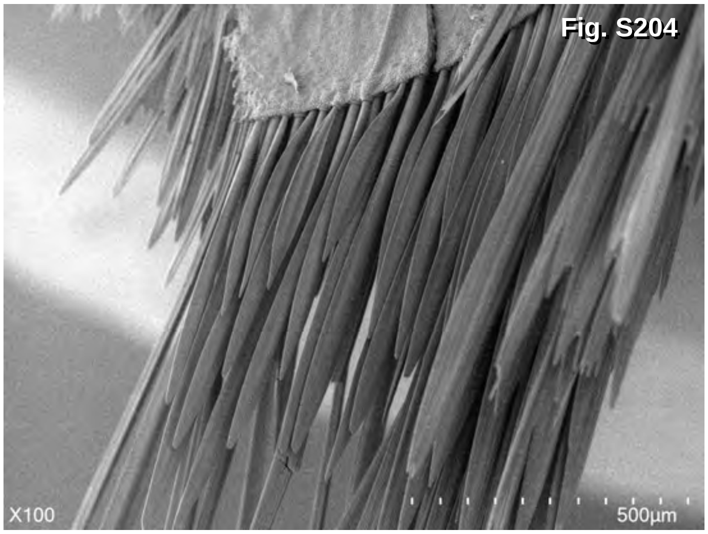

GP0046_Agrius_convolvuli_T8scales_1000x.tif

Megacorma obliqua

scale-on-spine

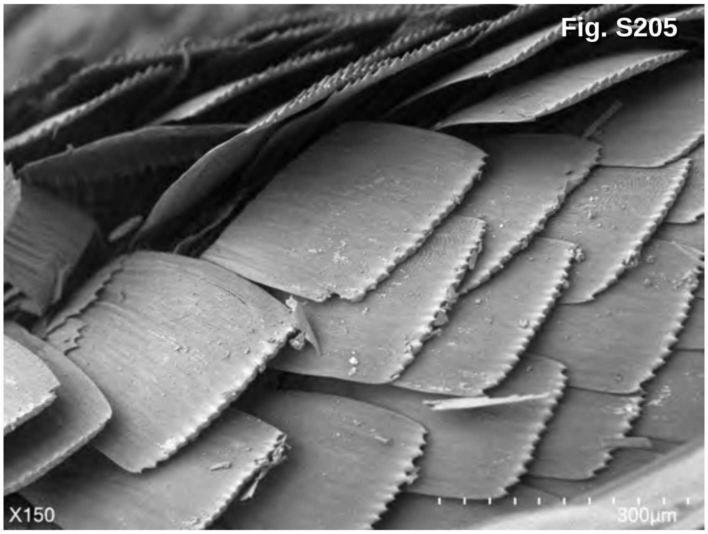

GP0044_Megacorma_obliqua_LeftValve_150x.tif

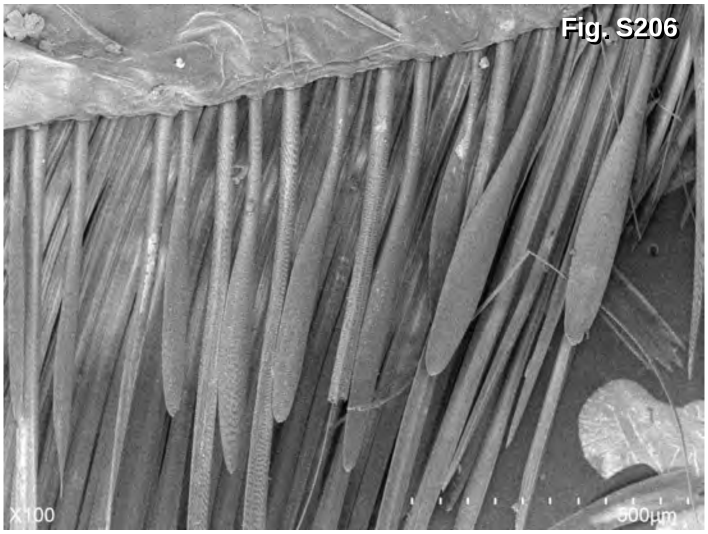

GP0044_Megacorma_obliqua_T8spines_100x.tif

Psilogramma anne

scale-on-spine

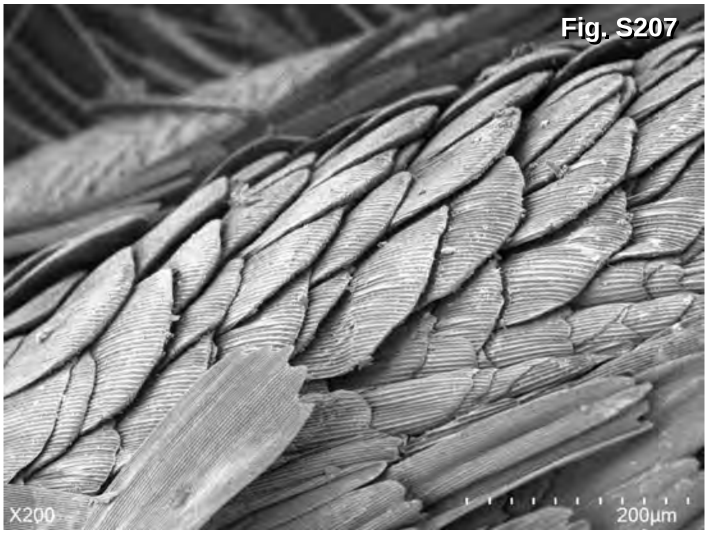

GP0051_Psilogramma_anne_LeftValve_200x.tif

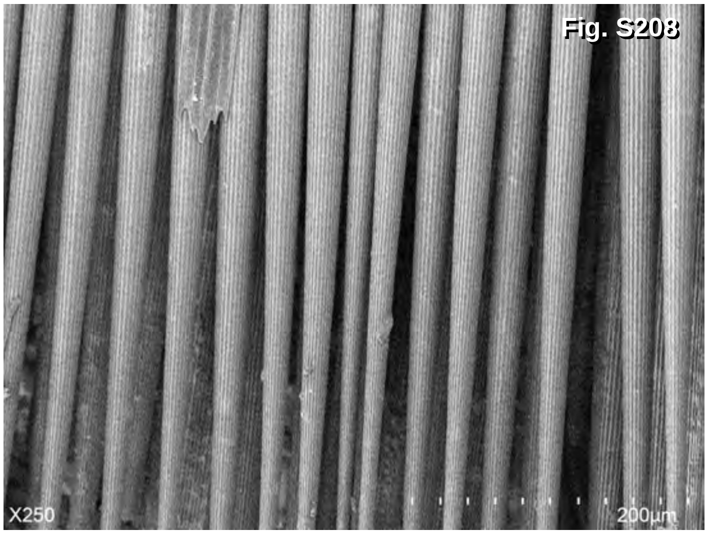

GP0051_Psilogramma_anne_T8scales_250x.tif

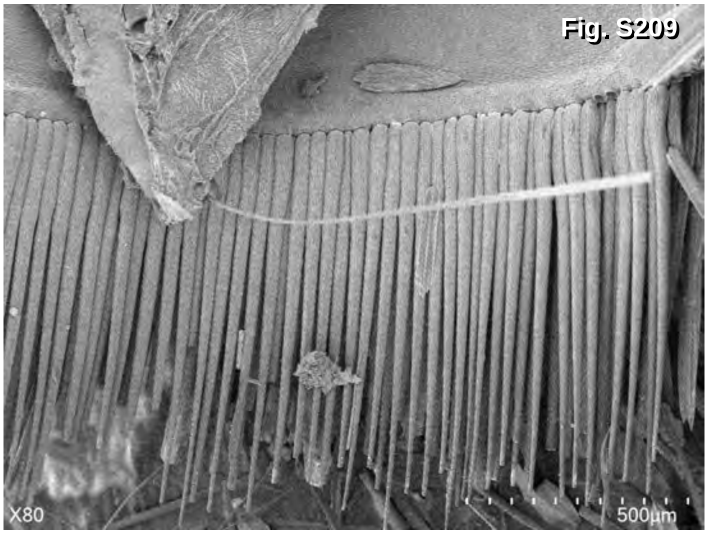

GP0051_Psilogramma_anne_T8spines_80x.tif

Xanthopan morganii

scale-on-spine

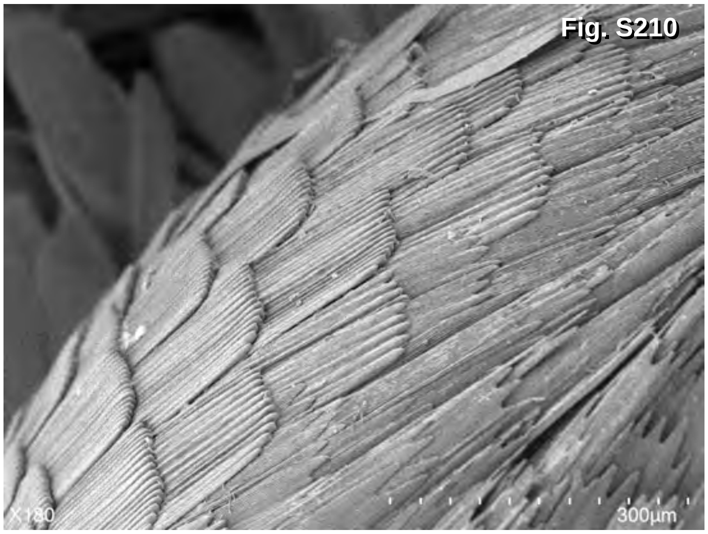

GP0045_Xanthopan_morganii_LeftValve_180x.tif

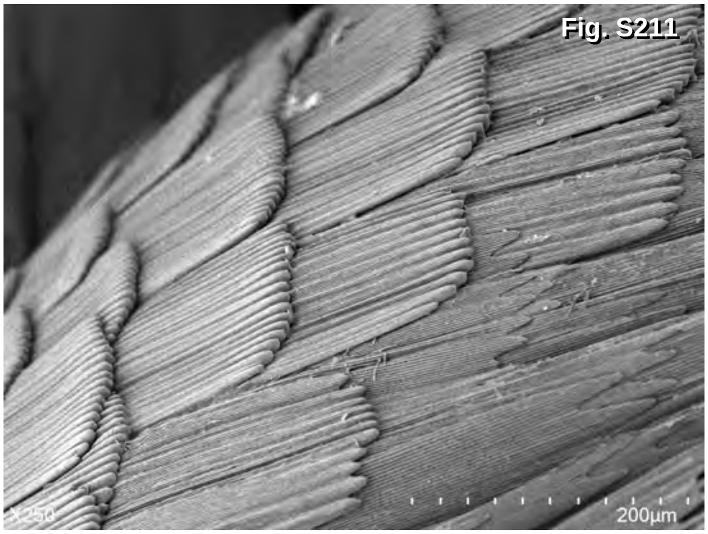

GP0045_Xanthopan_morganii_LeftValve_250x.tif

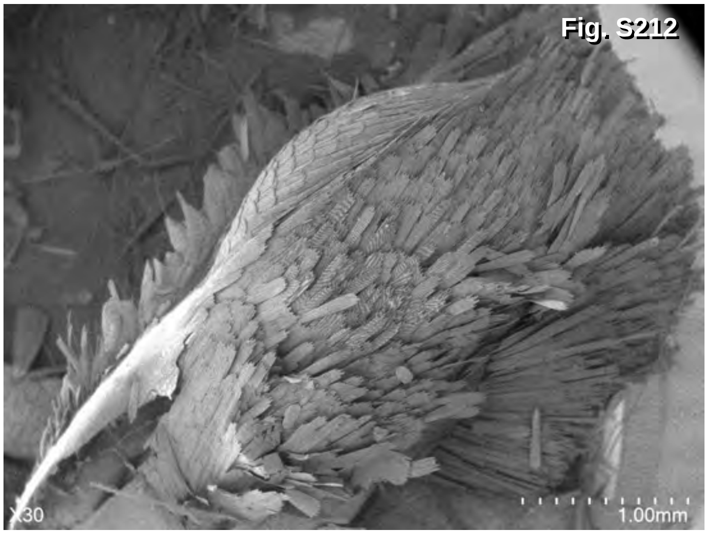

GP0045_Xanthopan_morganii_LeftValve_30x_2.tif

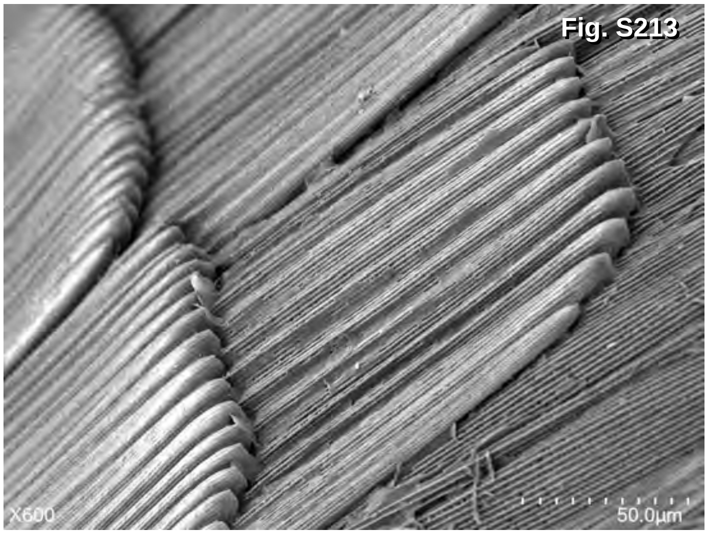

GP0045_Xanthopan_morganii_LeftValve_600x.tif

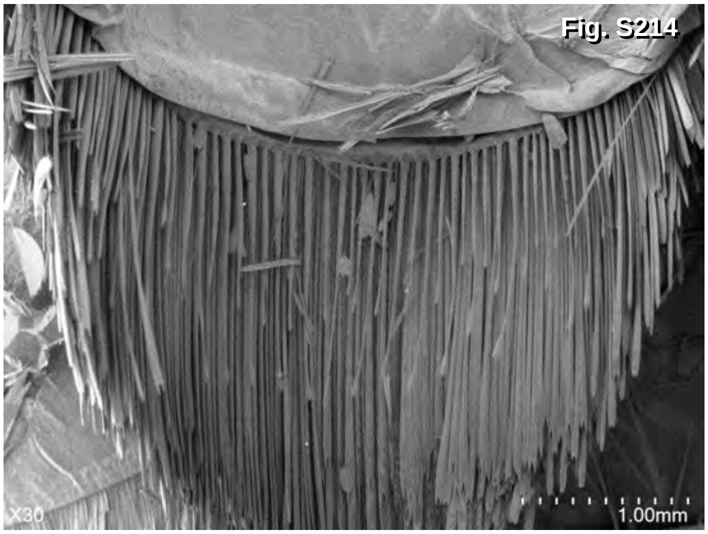

GP0045_Xanthopan_morganii_T8scales_30x.tif

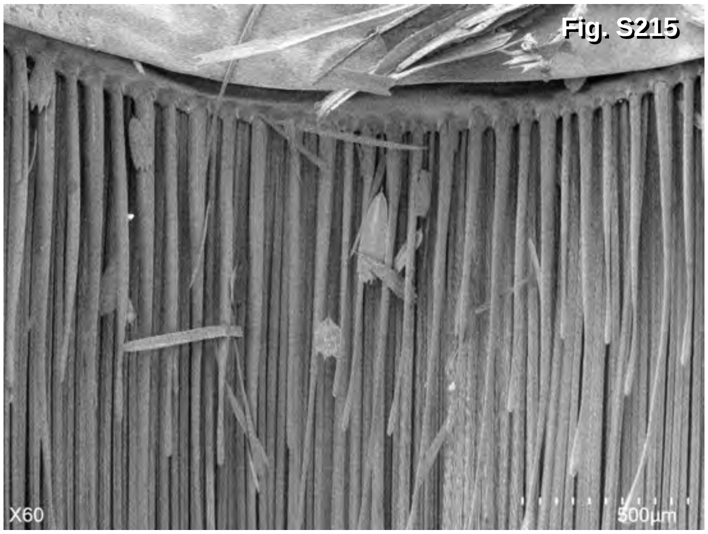

GP0045_Xanthopan_morganii_T8scales_60x.tif

Acosmeryx anceus

scale-on-nodule

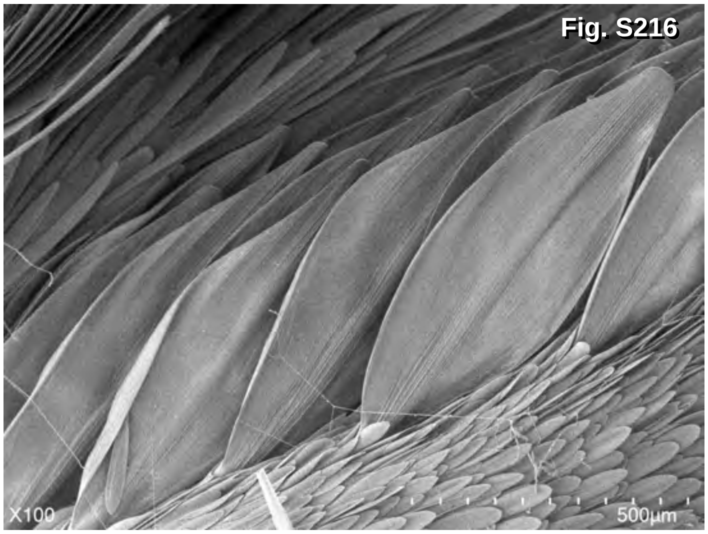

GP0015_Acosmeryx_anceus_LeftValve_100x.tif

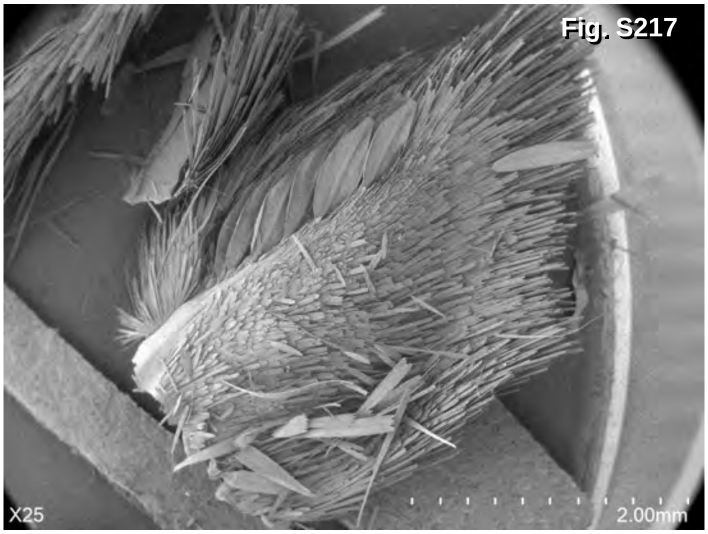

GP0015_Acosmeryx_anceus_LeftValve_25x.tif

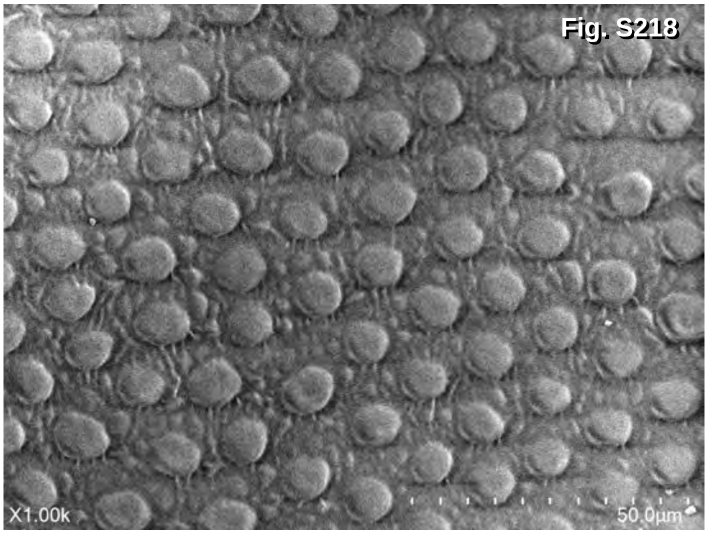

GP0015_Acosmeryx_anceus_T8_1000x.tif

Acosmeryx shervillii

scale-on-nodule

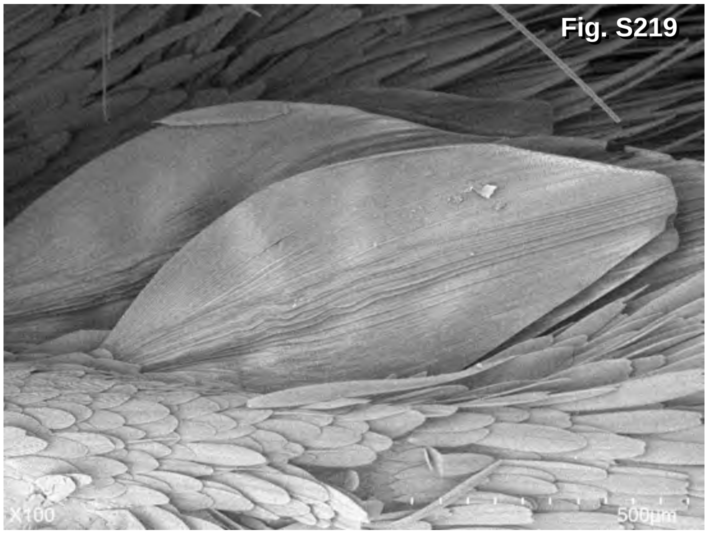

GP0014_Acosmeryx_shervillii_LeftValve_100x.tif

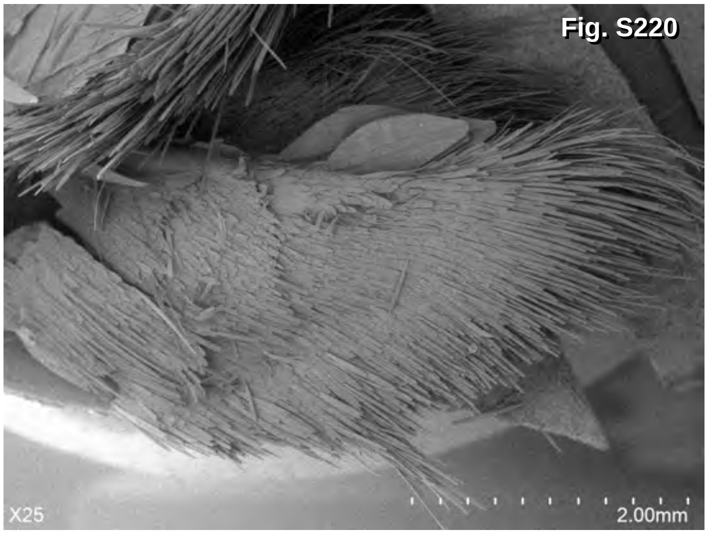

GP0014_Acosmeryx_shervillii_LeftValve_25x.tif

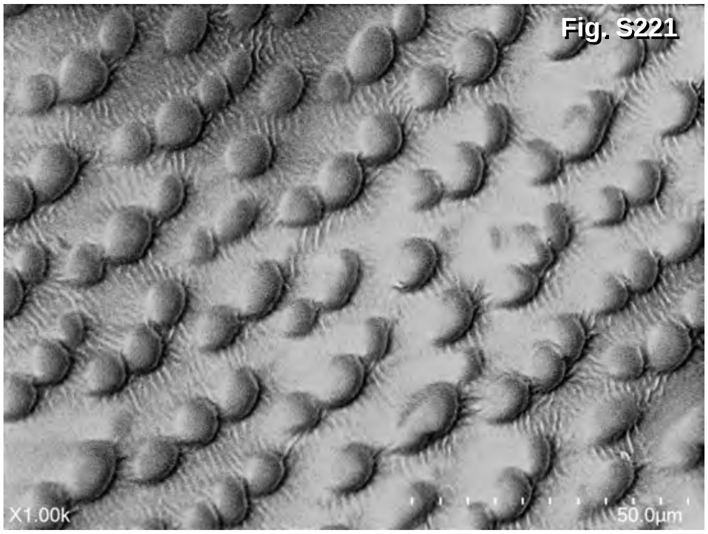

GP0014_Acosmeryx_shervillii_T8_1000x.tif

Ampelophaga rubiginosa

scale-on-nodule

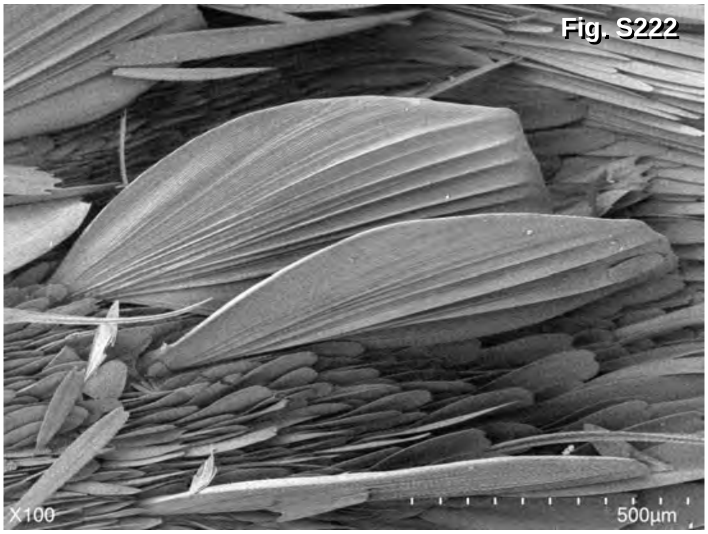

GP0013_Ampelophaga_rubiginosa_LeftValve_100x.tif

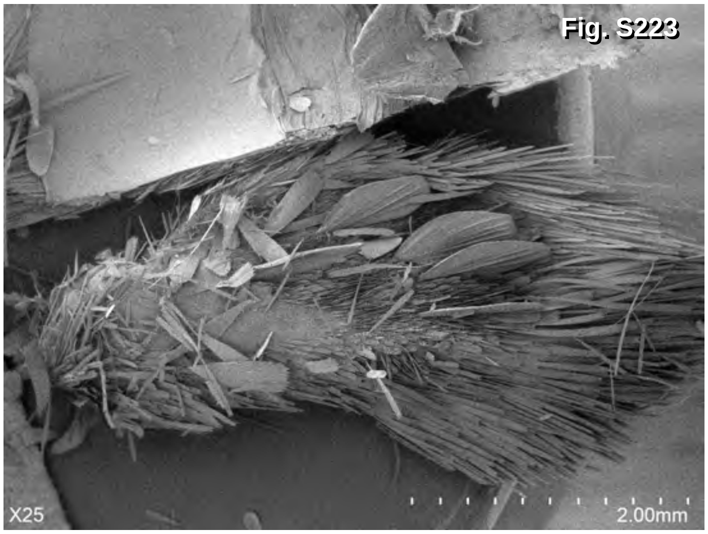

GP0013_Ampelophaga_rubiginosa_LeftValve_25x.tif

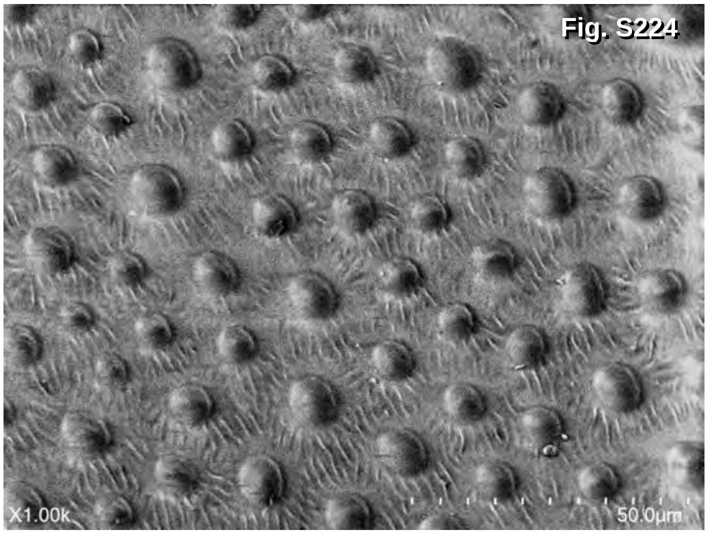

GP0013_Ampelophaga_rubiginosa_T8_1000x_2.tif

Callionima parce

scale-on-nodule

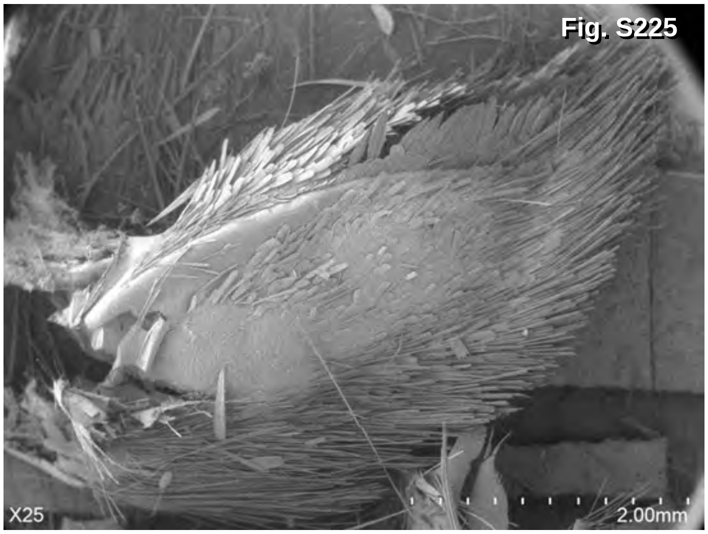

GP0033_Callionima_parce_LeftValve_25x.tif

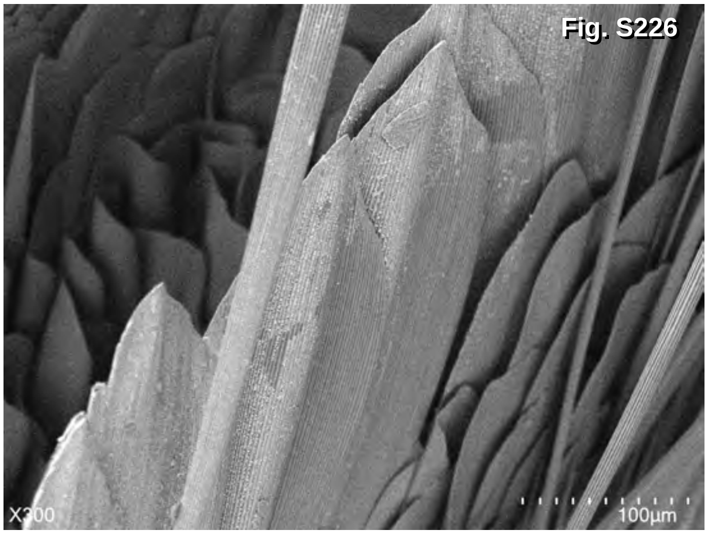

GP0069_Callionima_parce_LeftValve_x300.tif

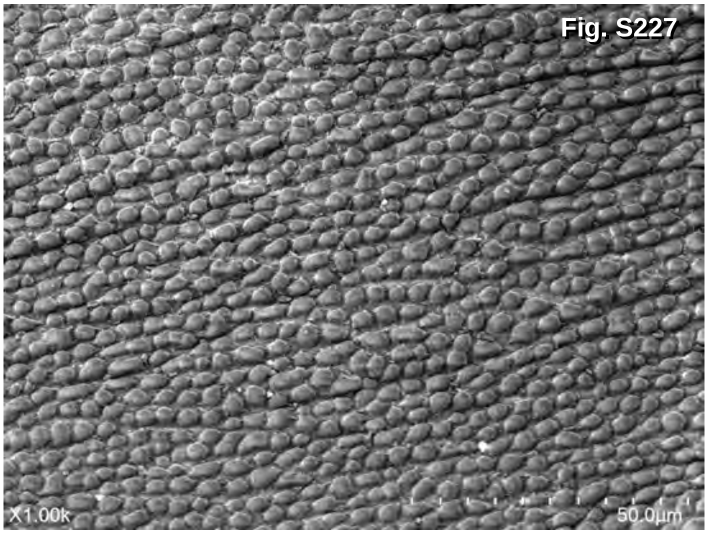

GP0069_Callionima_parce_T8_x1000.tif

Cechenena helops

scale-on-nodule

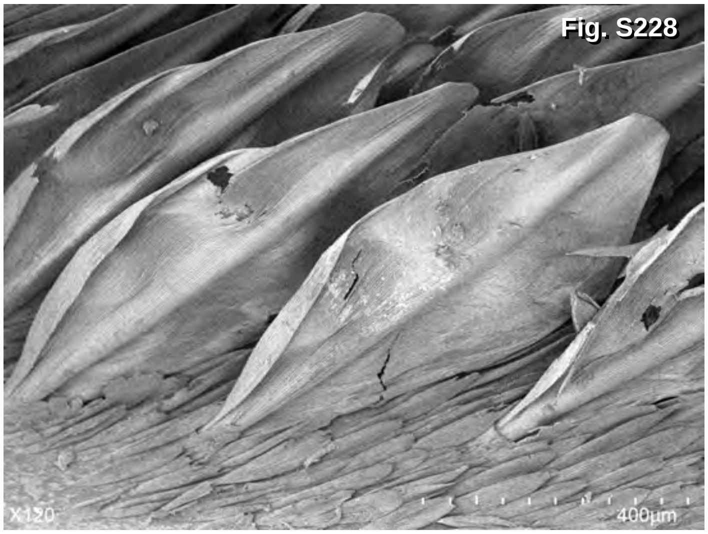

GP0008_Cechenena_helops_LeftValve_120x.tif

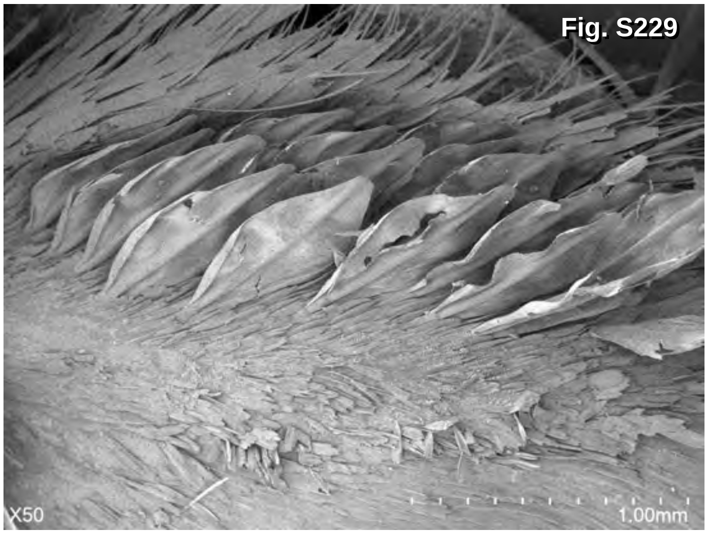

GP0008_Cechenena_helops_LeftValve_50x.tif

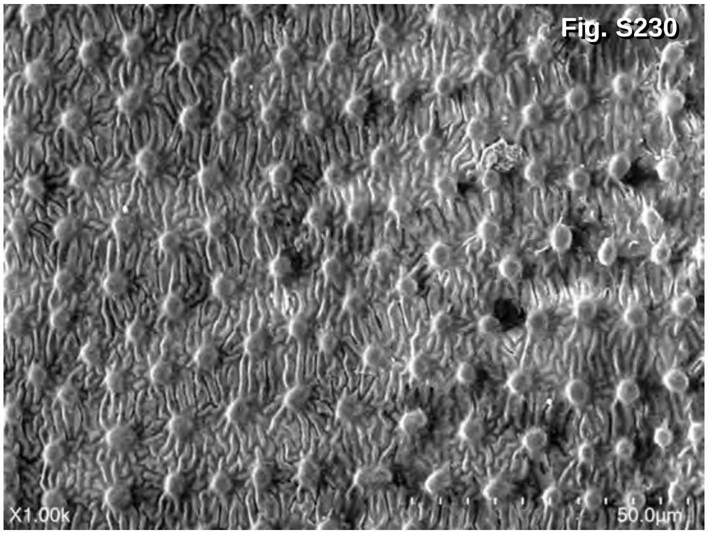

GP0008_Cechenena_helops_T8_1000x_2.tif

Daphnis hypothous

scale-on-nodule

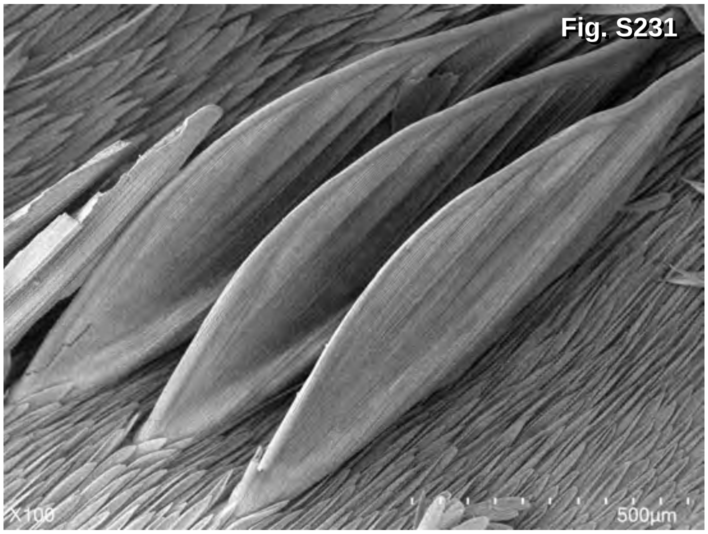

GP0011_Daphnis_hypothous_LeftValve_100x.tif

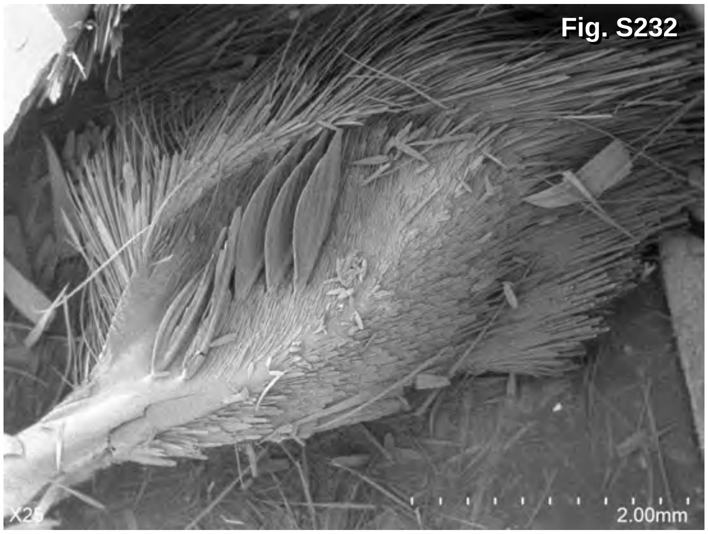

GP0011_Daphnis_hypothous_LeftValve_25x.tif

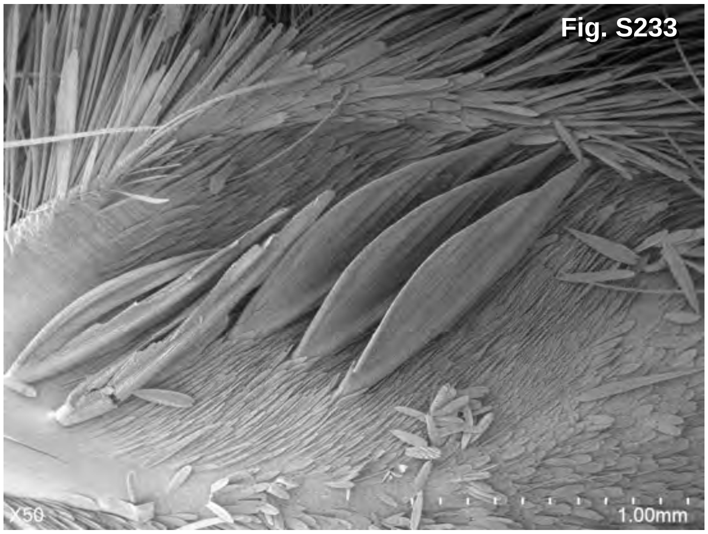

GP0011_Daphnis_hypothous_LeftValve_50x.tif

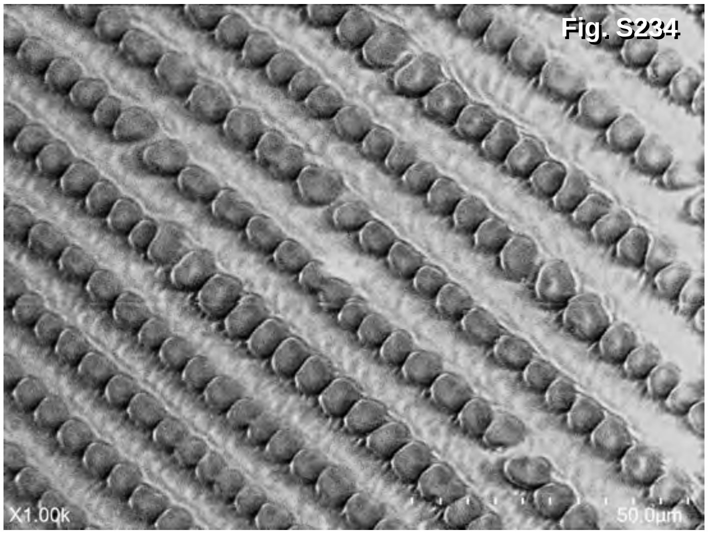

GP0011_Daphnis_hypothous_T8_1000x.tif

Darapsa myron

scale-on-nodule

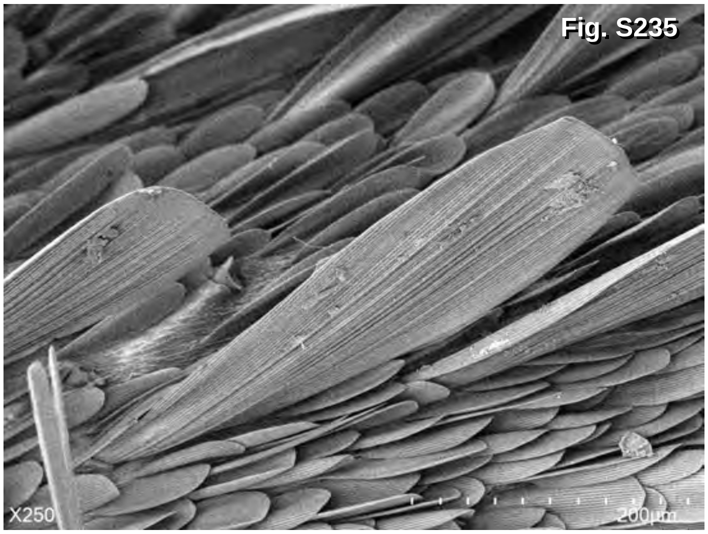

GP0009_Darapsa_myron_LeftValve_250x_2.tif

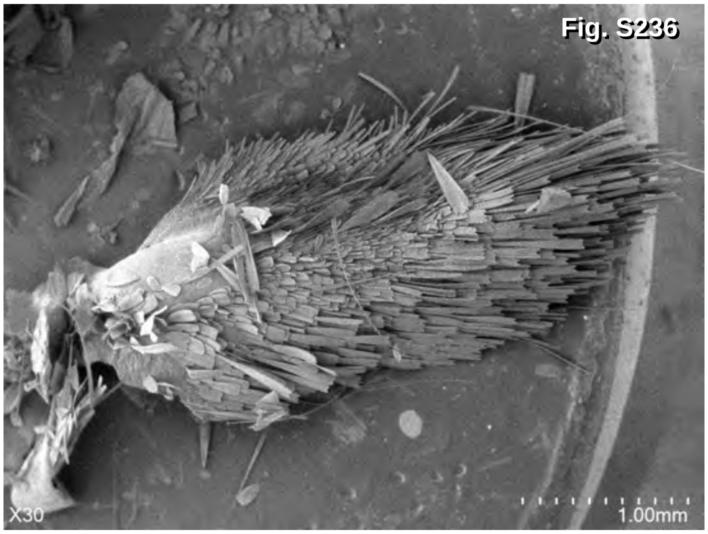

GP0009_Darapsa_myron_LeftValve_30x.tif

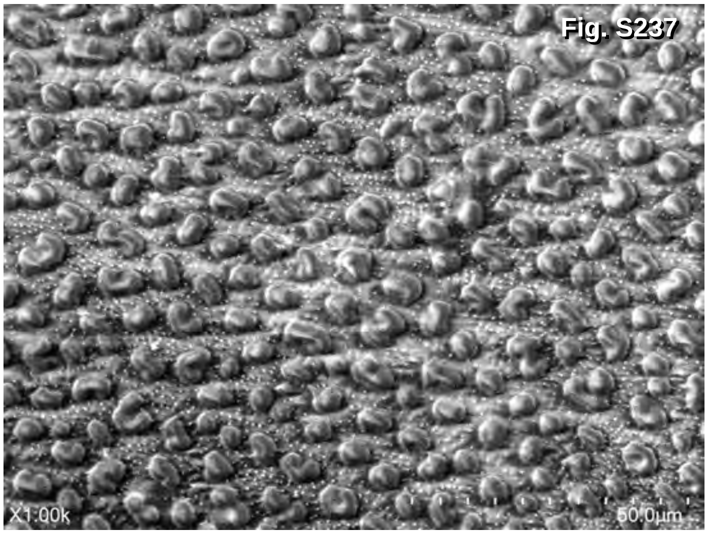

GP0009_Darapsa_myron_T8_1000x_2.tif

Elibia dolichus

scale-on-nodule

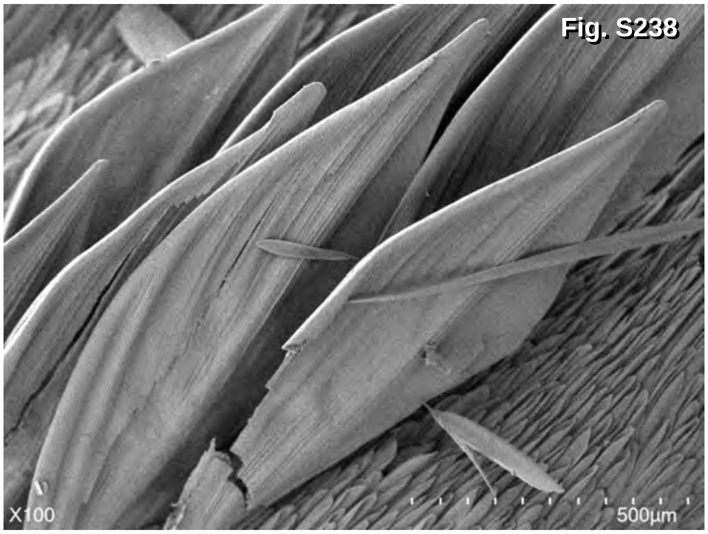

GP0012_Elibia_dolichus_LeftValve_100x.tif

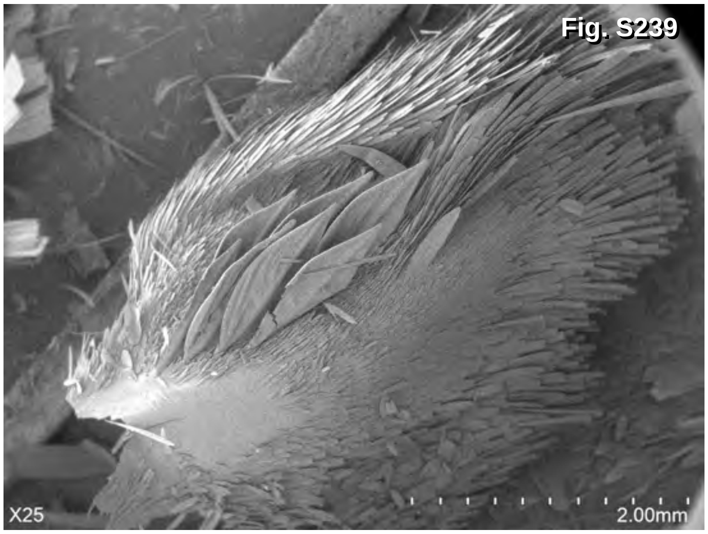

GP0012_Elibia_dolichus_LeftValve_25x.tif

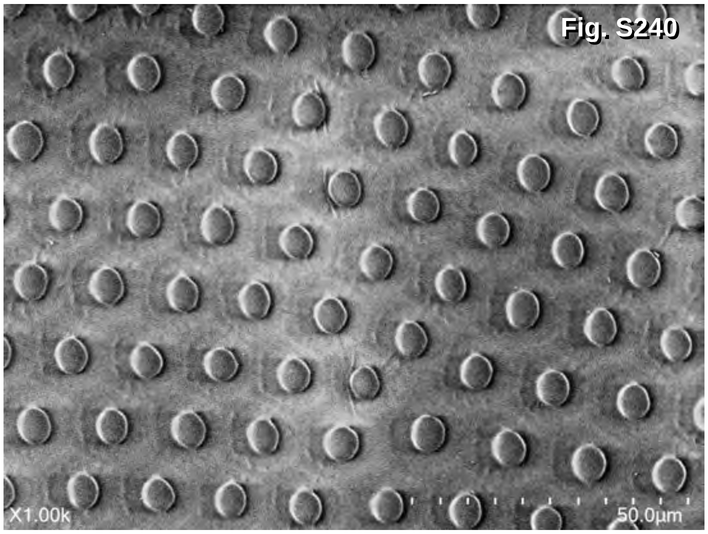

GP0012_Elibia_dolichus_T8_1000x.tif

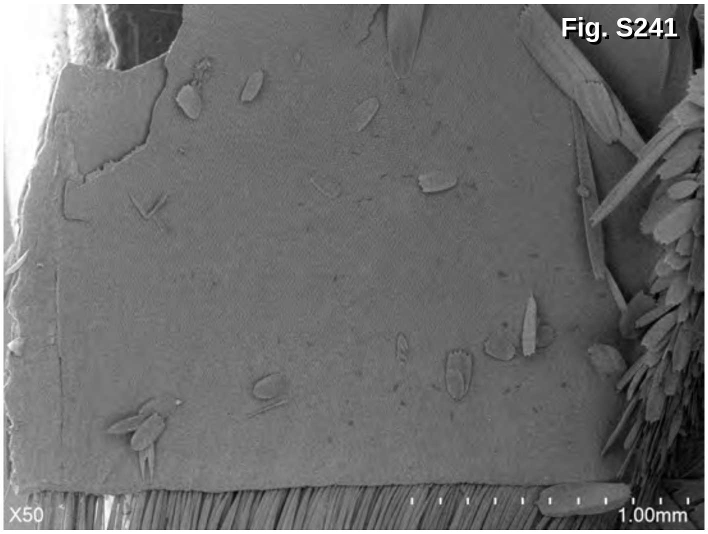

GP0012_Elibia_dolichus_T8_50x.tif

Enpinanga borneensis

scale-on-nodule

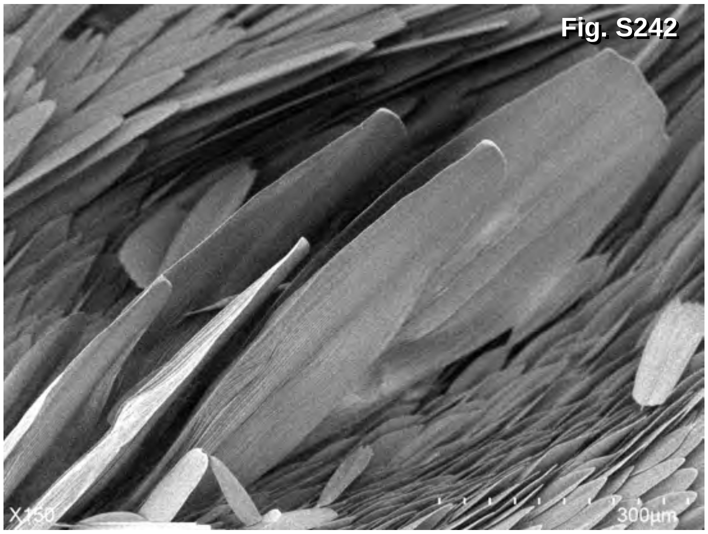

GP0017_Enpinanga_borneensis_LeftValve_150x.tif

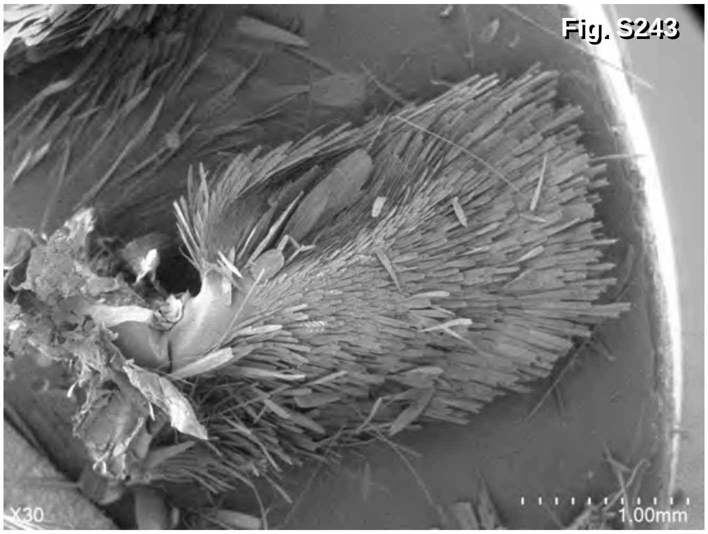

GP0017_Enpinanga_borneensis_LeftValve_30x.tif

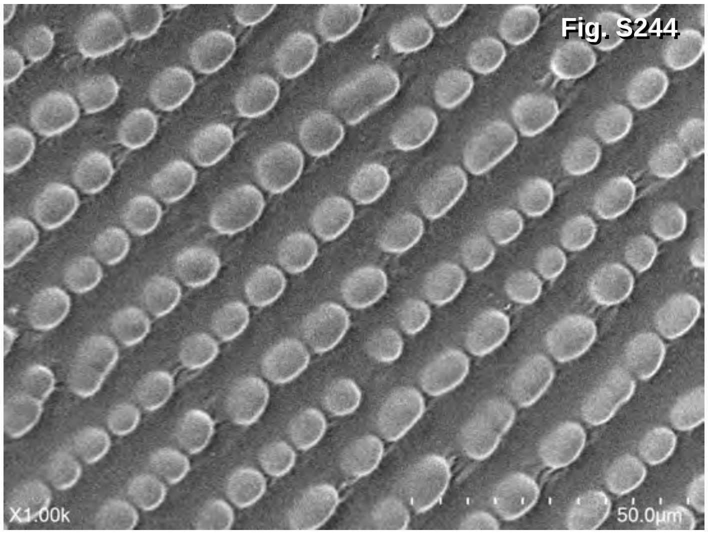

GP0017_Enpinanga_borneensis_T8_1000x.tif

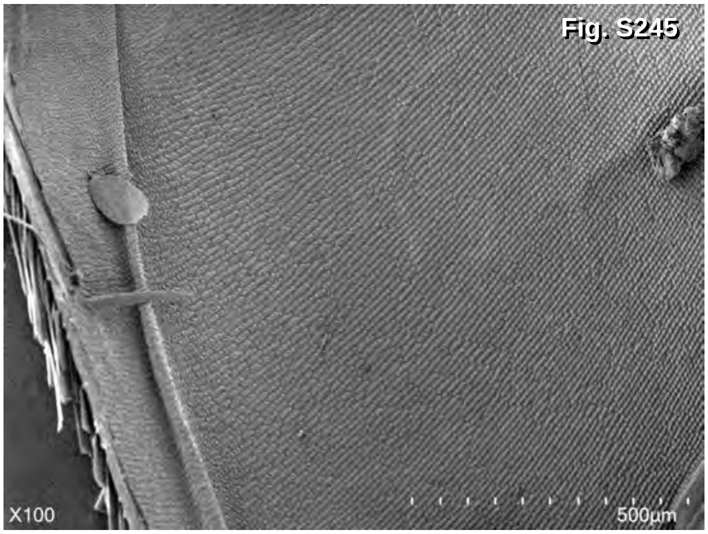

GP0017_Enpinanga_borneensis_T8_100x.tif

Eupanacra regularis

scale-on-nodule

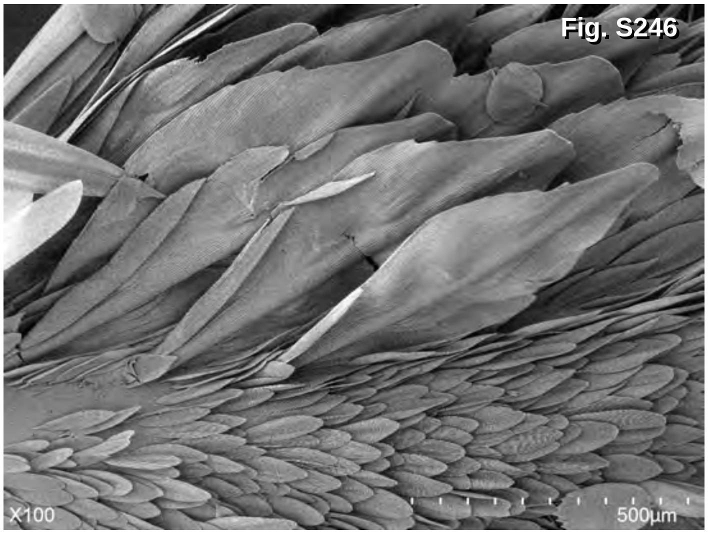

GP0016_Eupanacra_regularis_LeftValve_100x.tif

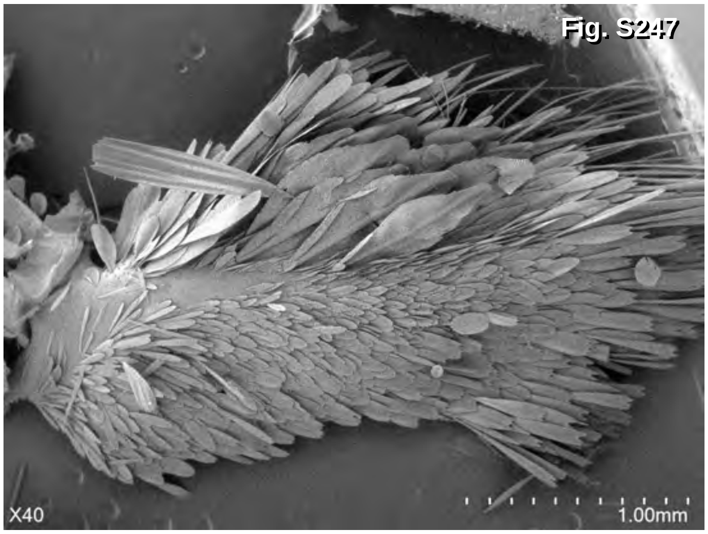

GP0016_Eupanacra_regularis_LeftValve_40x.tif

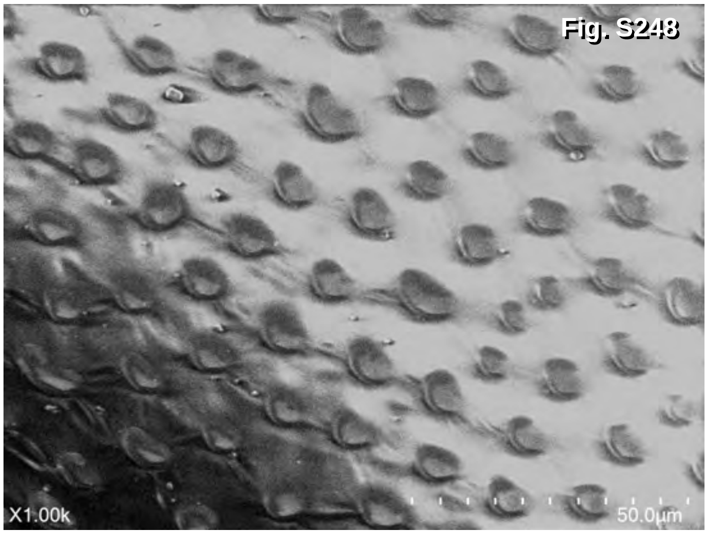

GP0016_Eupanacra_regularis_T8_1000x.tif

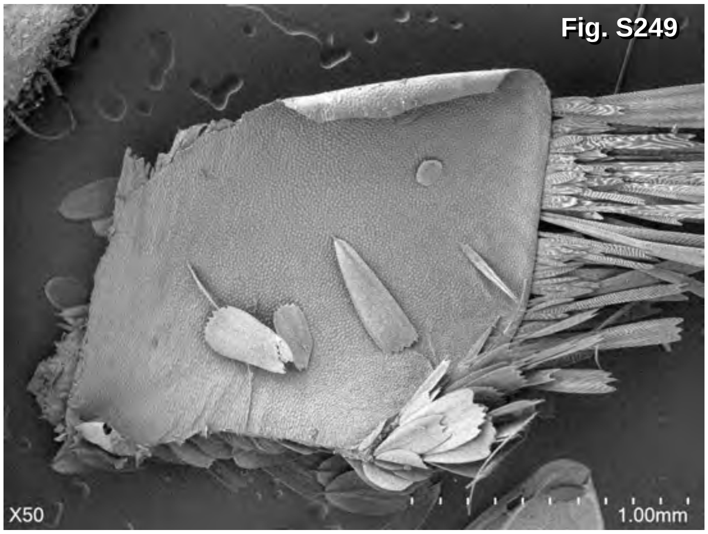

GP0016_Eupanacra_regularis_T8_50x.tif

Hippotion rosetta

scale-on-nodule

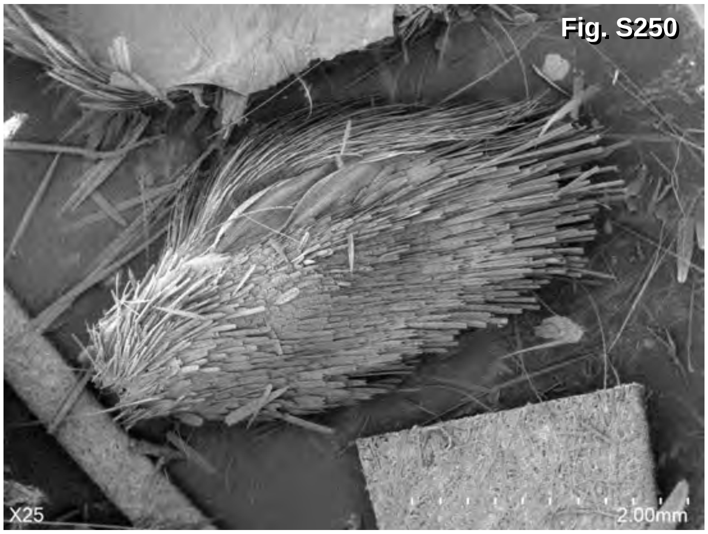

GP0007_Hippotion_rosetta_LeftValve_25x.tif

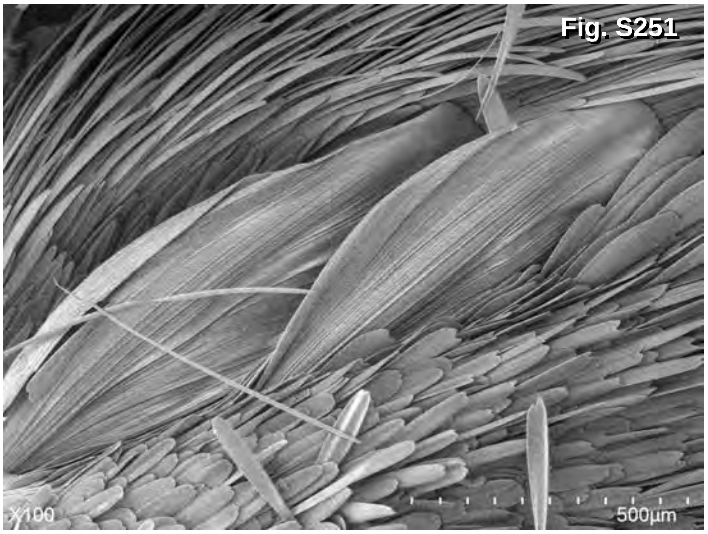

GP0007_Hippotion_rosetta_LeftValve_100x.tif

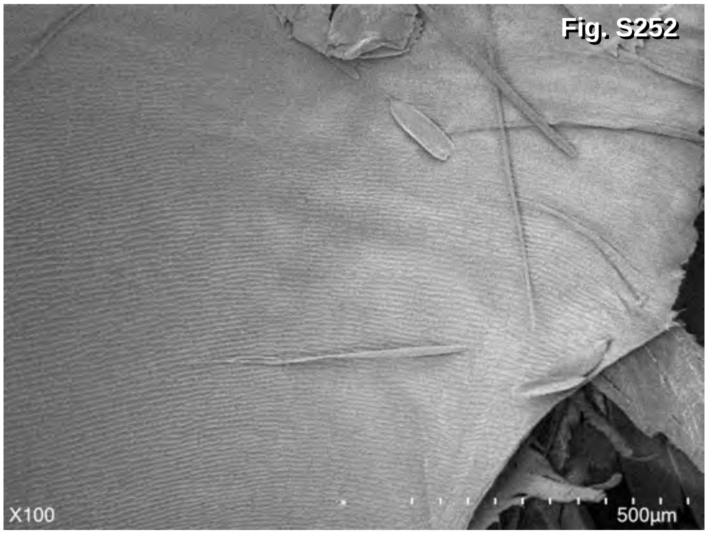

GP0007_Hippotion_rosetta_T8_100x.tif

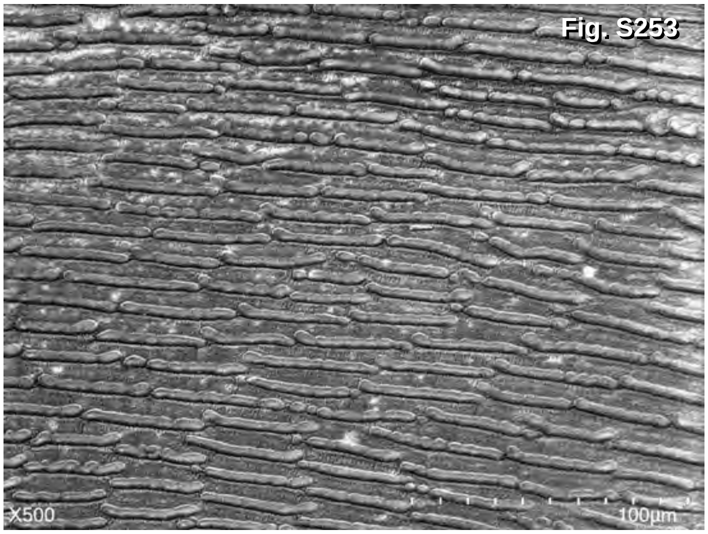

GP0007_Hippotion_rosetta_T8_500x.tif

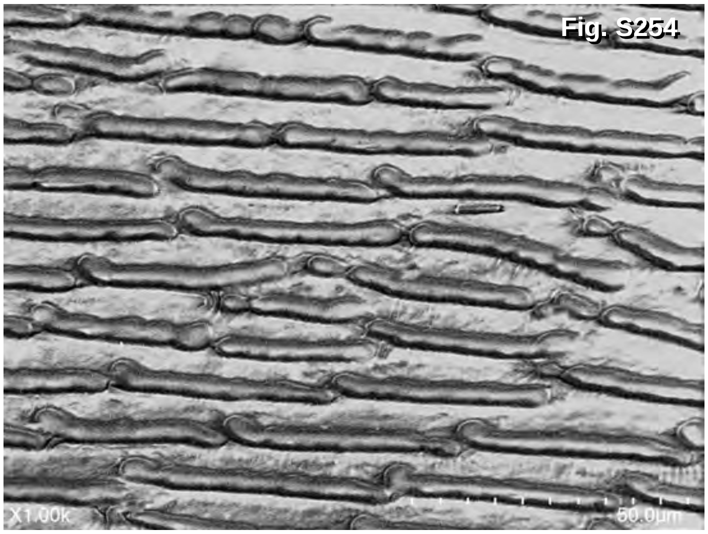

GP0007_Hippotion_rosetta_T8_1000x.tif

Isognathus rimosa

scale-on-nodule

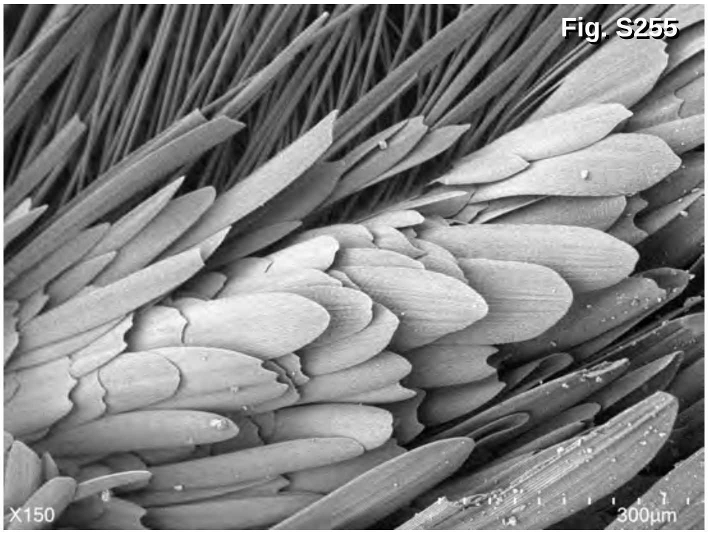

GP0029_Isognathus_rimosa_LeftValve_150x.tif

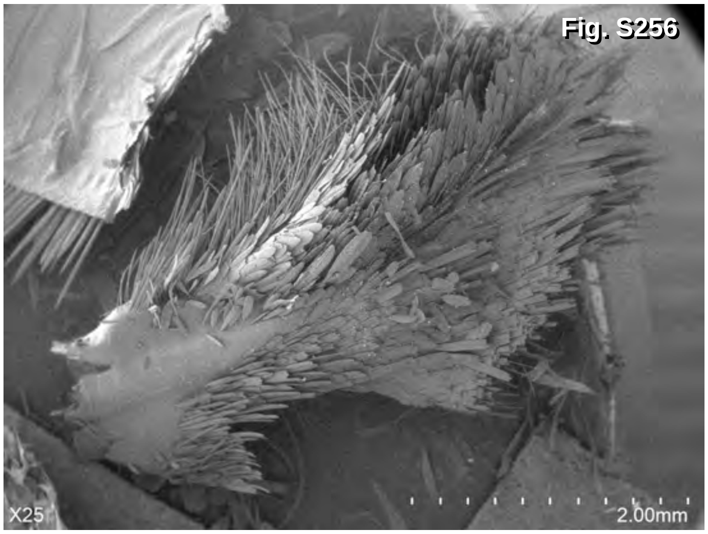

GP0029_Isognathus_rimosa_LeftValve_25x.tif

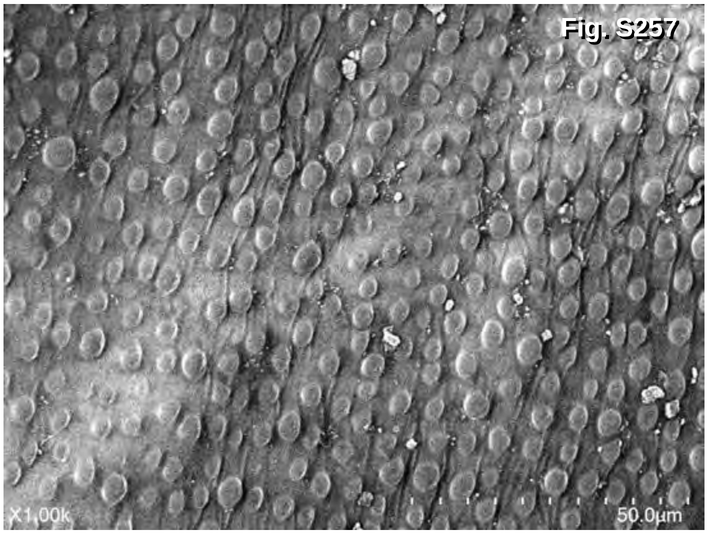

GP0029_Isognathus_rimosa_T8_1000x.tif

Nyceryx coffaeae

scale-on-nodule

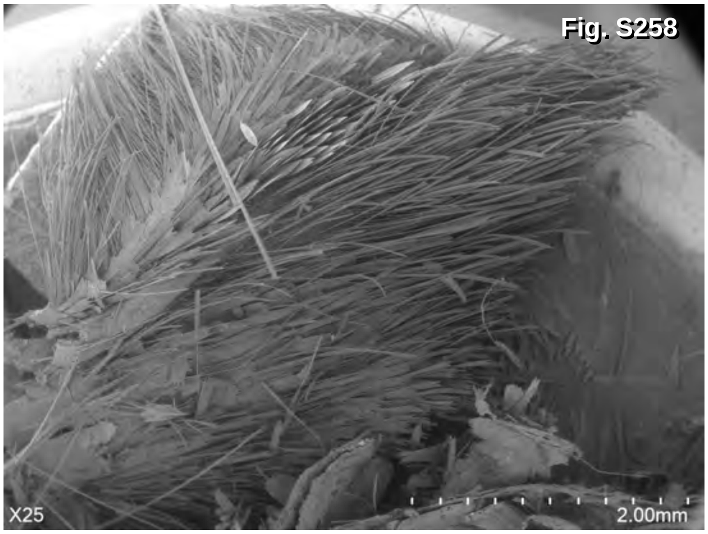

GP0034_Nyceryx_coffaeae_LeftValve_25x.tif

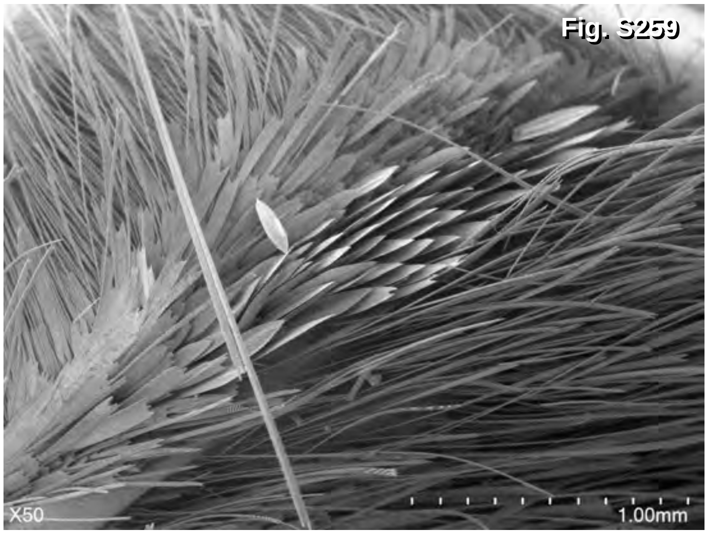

GP0034_Nyceryx_coffaeae_LeftValve_50x.tif

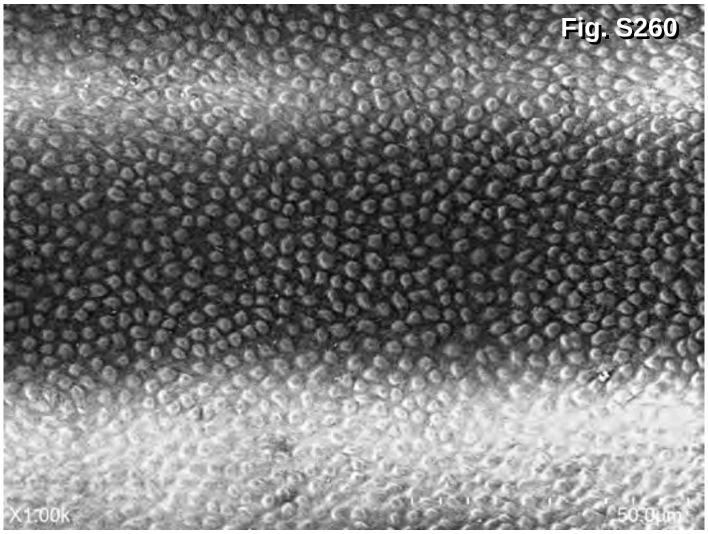

GP0034_Nyceryx_coffaeae_T8_1000x.tif

Nyceryx magna

scale-on-nodule

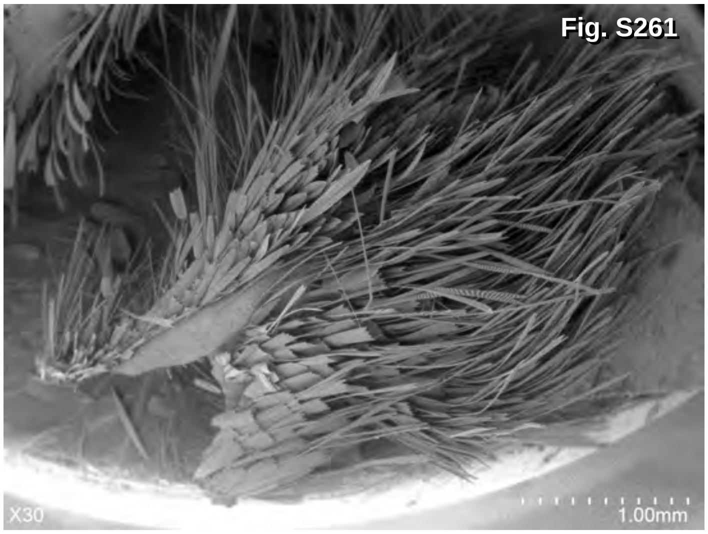

GP0038_Nyceryx_magna_LeftValve_30x.tif

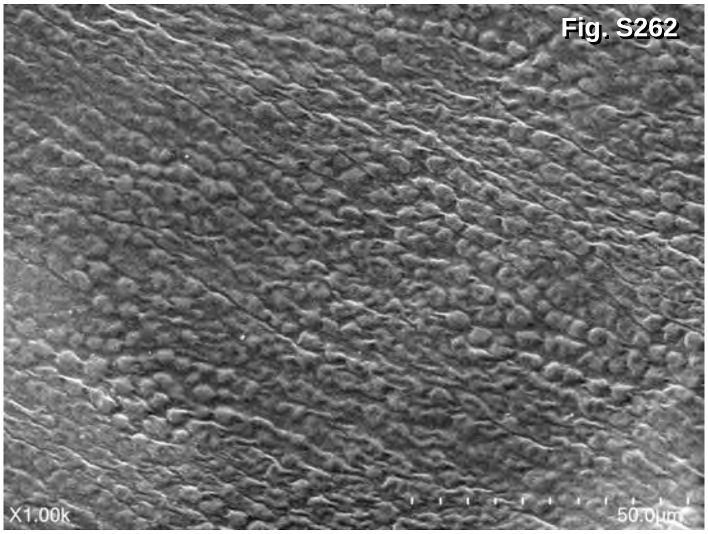

GP0038_Nyceryx_magna_T8_1000x.tif

Theretra nessus

scale-on-nodule

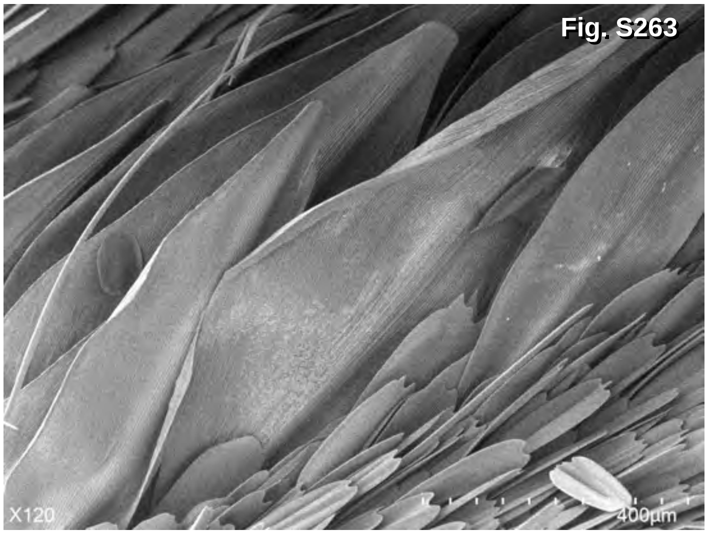

GP0005_Theretra_nessus_LeftValve_120x.tif

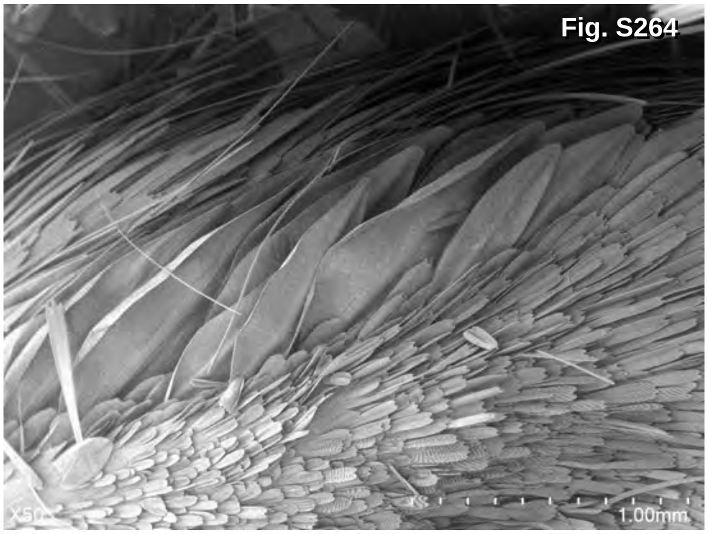

GP0005_Theretra_nessus_LeftValve_50x.tif

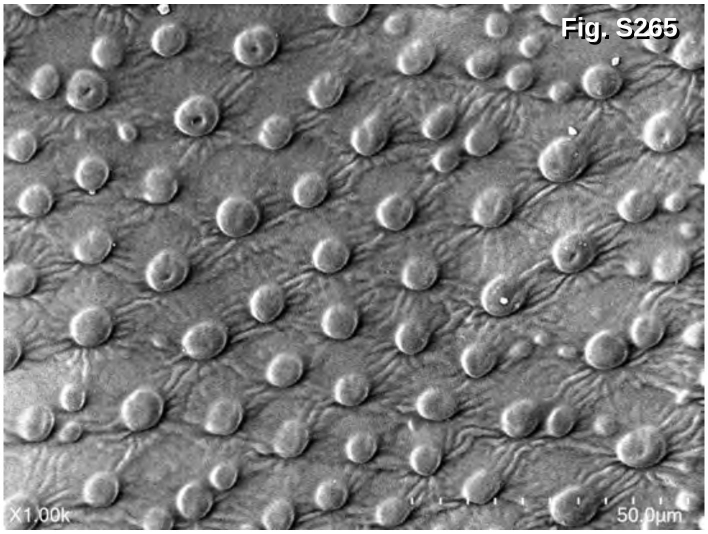

GP0005_Theretra_nessus_T8_1000x.tif

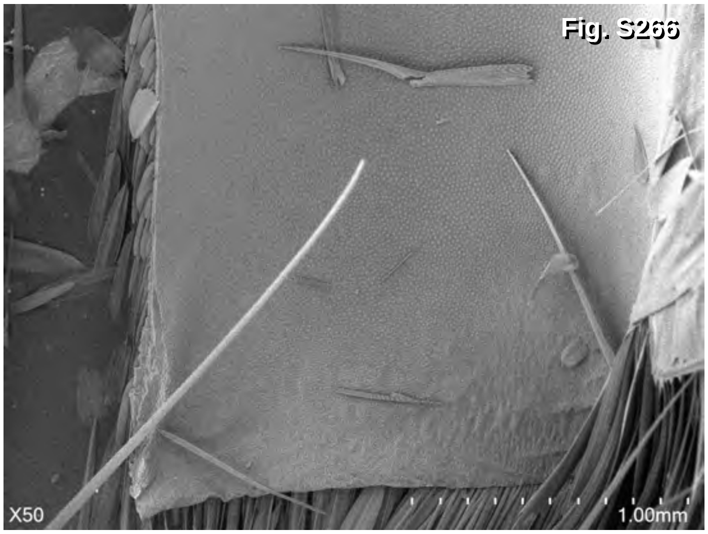

GP0005_Theretra_nessus_T8_50x.tif

Theretra rhesus

scale-on-nodule

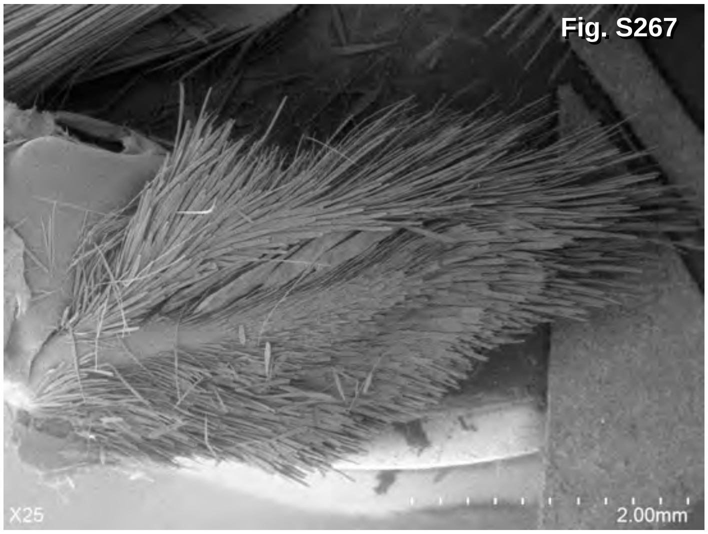

GP0004_Theretra_rhesus_LeftValve_25x.tif

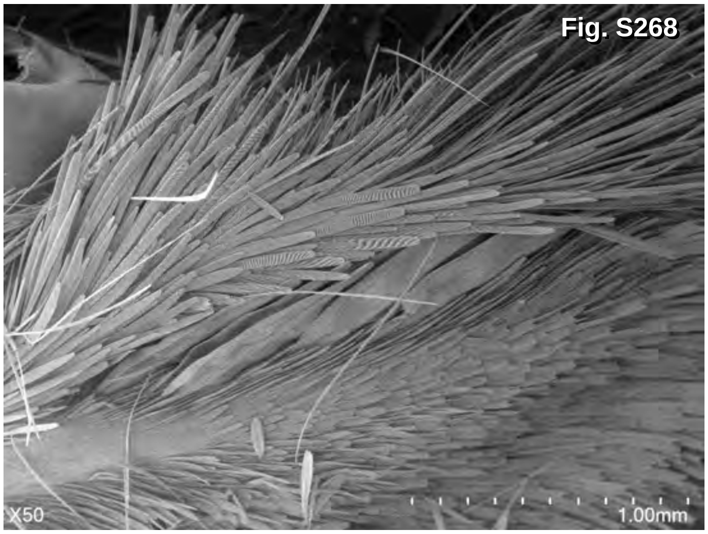

GP0004_Theretra_rhesus_LeftValve_50x.tif

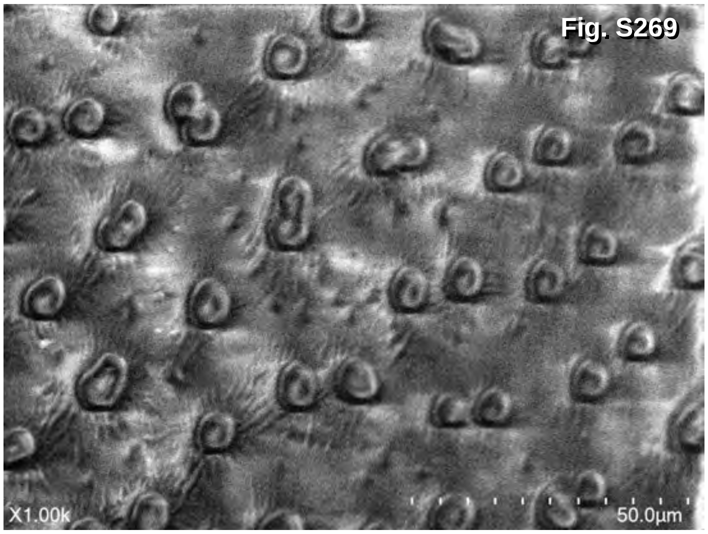

GP0004_Theretra_rhesus_T8_1000x_2.tif

Xylophanes amadis

scale-on-nodule

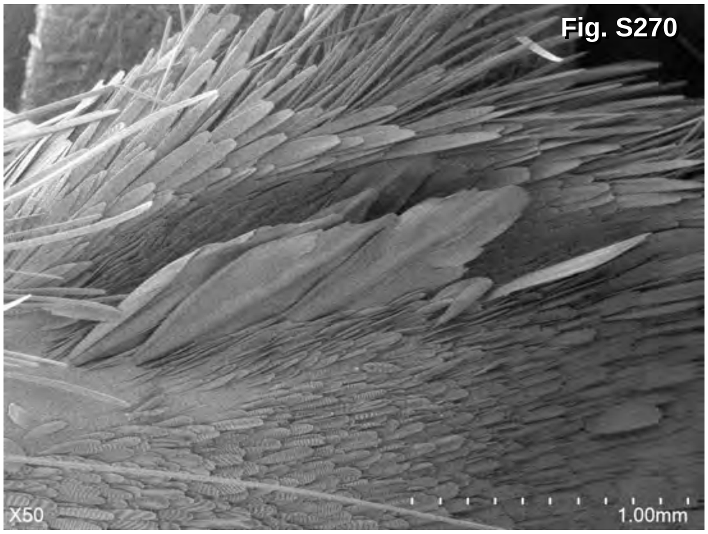

GP0003_Xylophanes_amadis_LeftValve_50x.tif

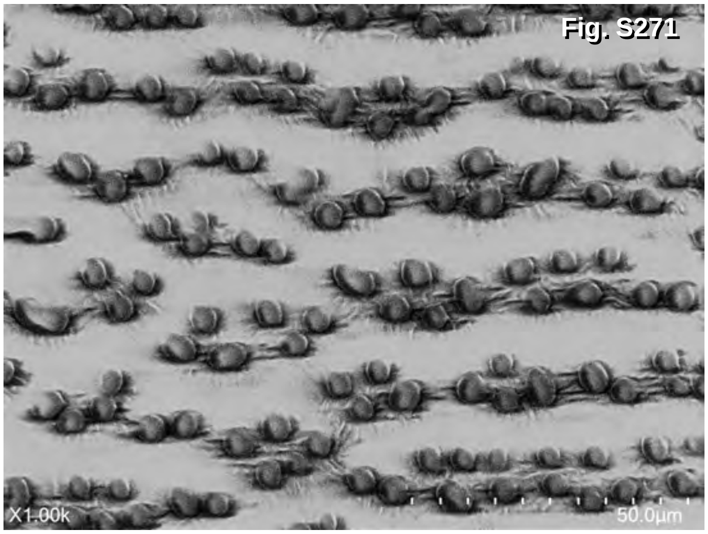

GP0003_Xylophanes_amadis_T8_1000x.tif

Xylophanes falco

scale-on-nodule

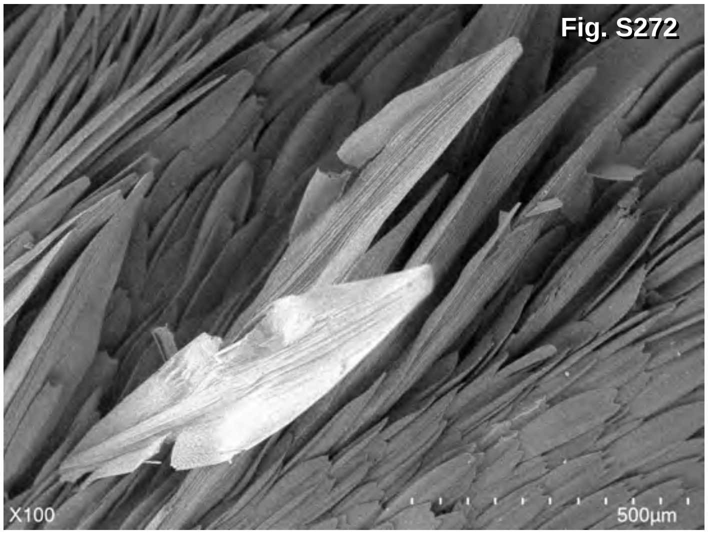

GP0060_Xylophanes_falco_LeftValve_100x.tif

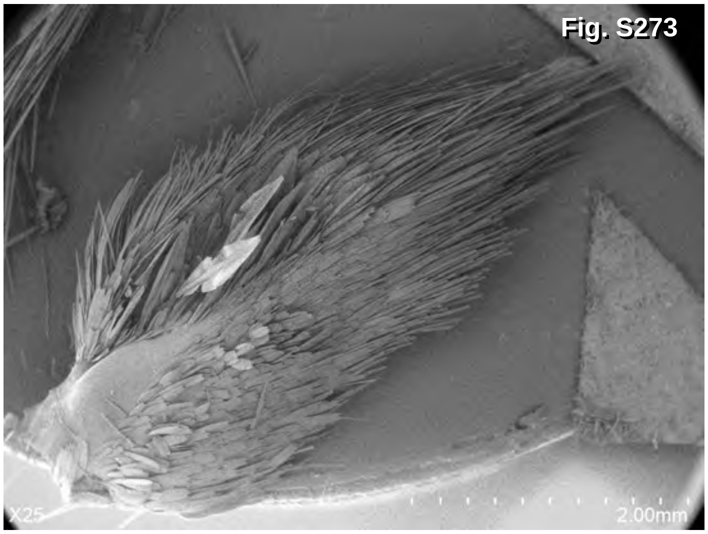

GP0060_Xylophanes_falco_LeftValve_25x.tif

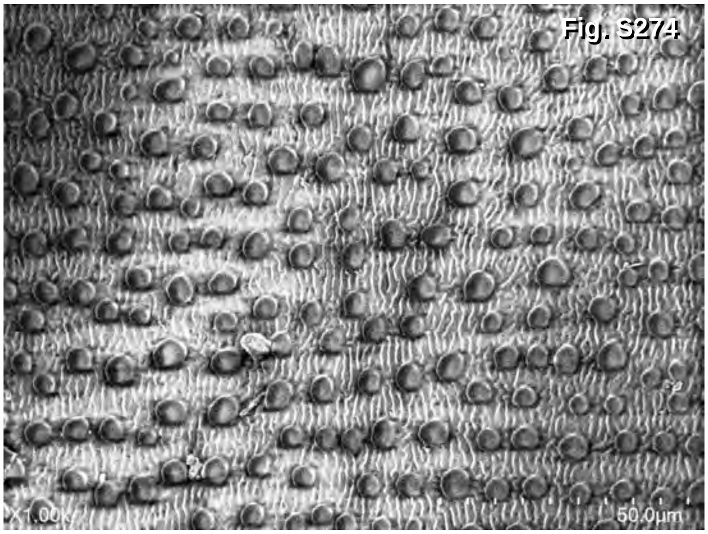

GP0060_Xylophanes_falco_T8_1000x.tif

Xylophanes pluto

scale-on-nodule

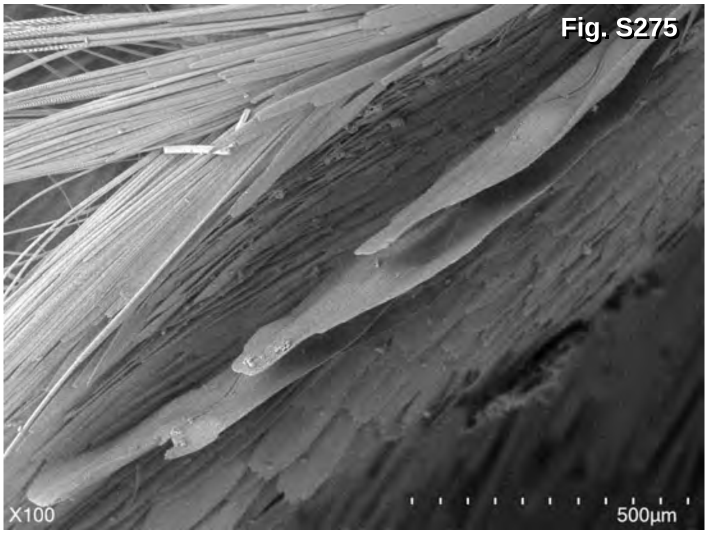

GP0070_Xylophanes_pluto_RightValve_x100.tif

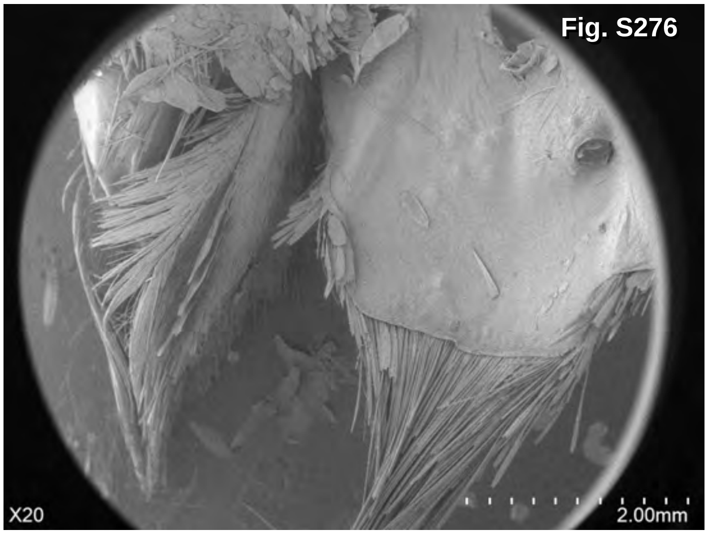

GP0070_Xylophanes_pluto_RightValve+T8_x20_scales.tif

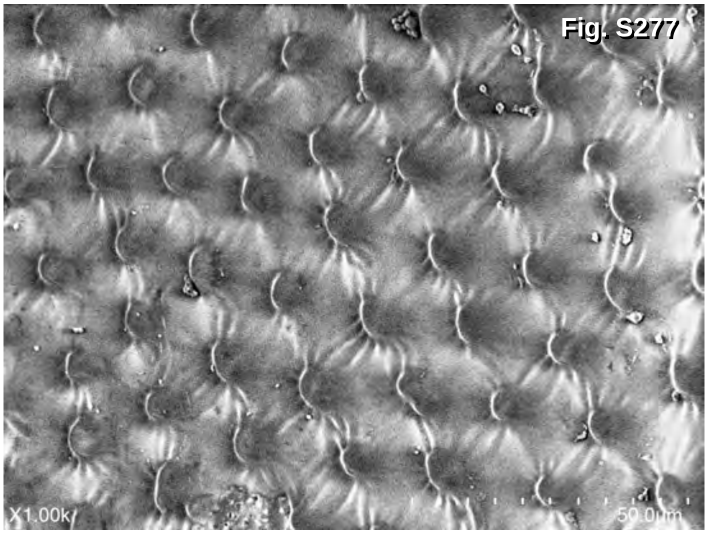

GP0070_Xylophanes_pluto_T8_x1000.tif

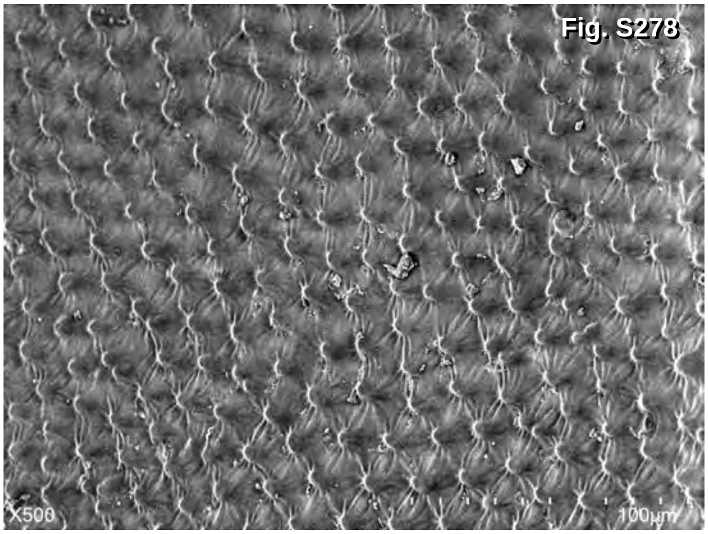

GP0070_Xylophanes_pluto_T8_x500.tif

Xylophanes tersa

scale-on-nodule

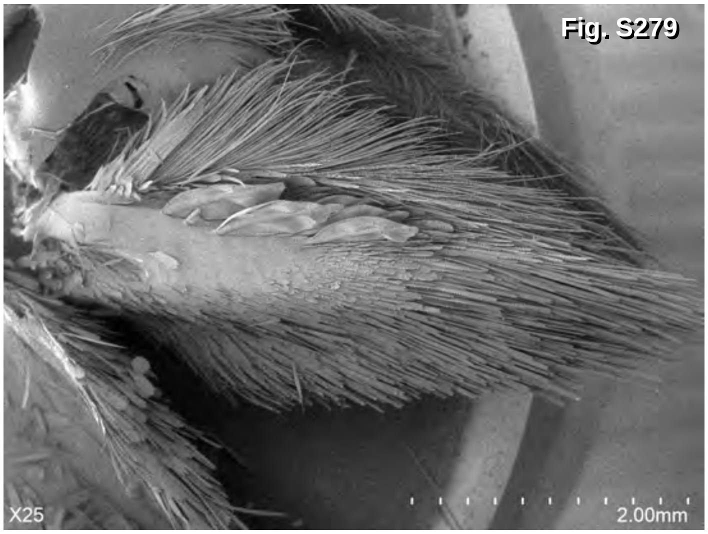

GP0002_Xylophanes_tersa_LeftValve_25x.tif

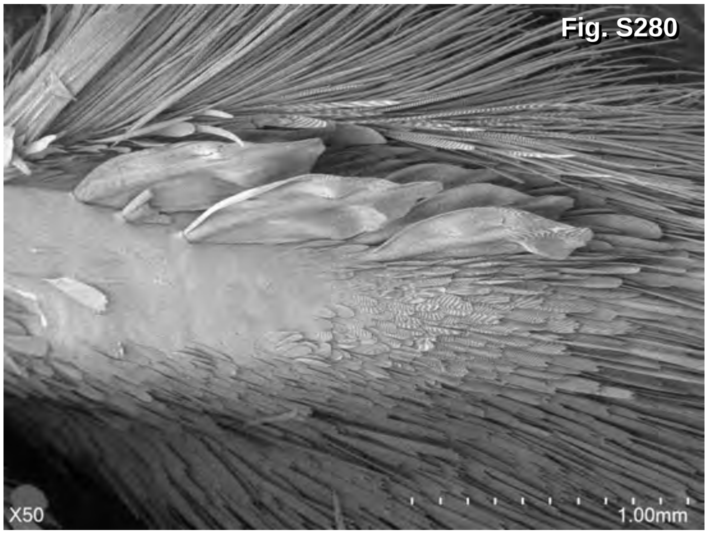

GP0002_Xylophanes_tersa_LeftValve_50x.tif

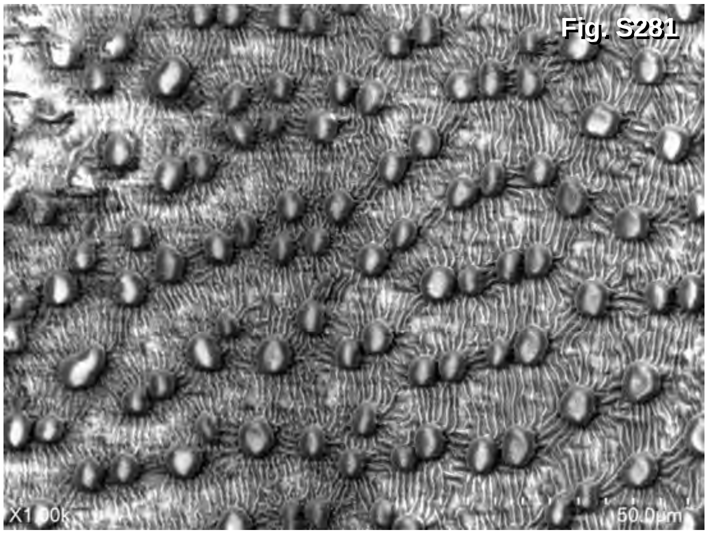

GP0002_Xylophanes_tersa_T8_1000x_4.tif

Xylophanes thyelia

scale-on-nodule

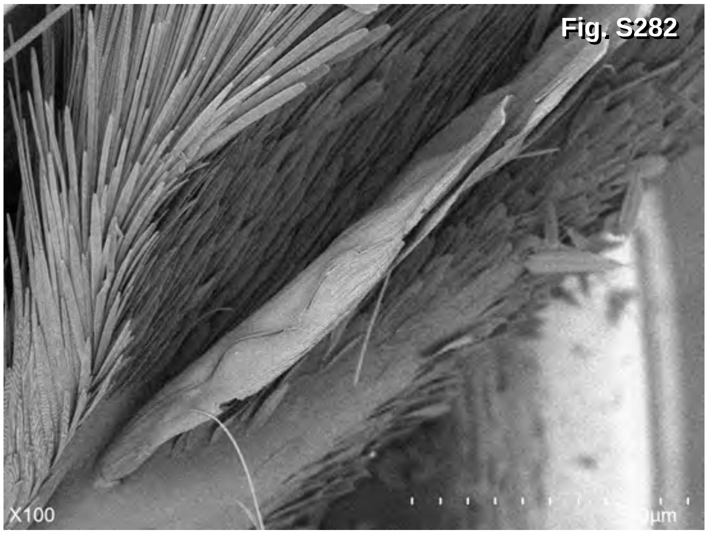

GP0001_Xylophanes_thyelia_LeftValve_100x_2.tif

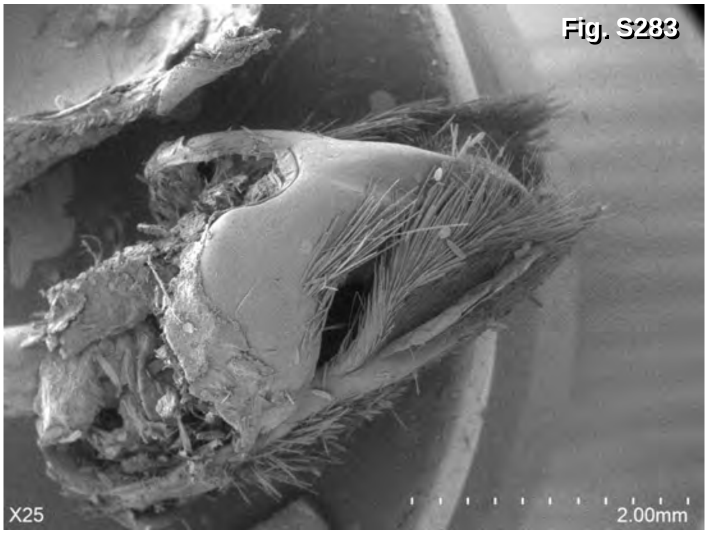

GP0001_Xylophanes_thyelia_LeftValve_25x_4.tif

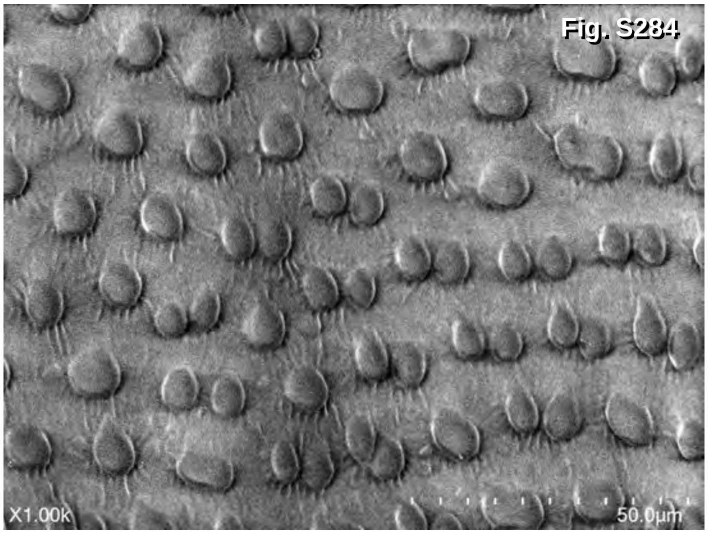

GP0001_Xylophanes_thyelia_T8_1000x_4.tif
