# Supplementary material for: The repeated evolution of anti-bat bioacoustics in hawkmoths
Source: Proc Biol Sci. 2026 Nov 4;293(2082):20262303. doi: 10.1098/rspb.2026.2303 (PMC13730811; doi:10.1098/rspb.2026.2303)
Supplement: Supplementary file 1 [file rspb.2026.2303.zip › rspb.2026.2303/rspb20262303_si_006.pdf]

SUPPLEMENTARY DATA README FILE

The repeated evolution of anti-bat bioacoustics in hawkmoths

Proceedings B 10.1098/rspb

<u>Data:</u>

DATA_for_ASR_Response_to_batcall_matrix_(fromTableS3).csv

Description: Data matrix for the ancestral state reconstruction of stridulatory response behavior used in the script "SCRIPT_ASR.R".

DATA_for_ASR_Stridulatory_System_Matrix_(fromTableS6).csv

Description: Data matrix for the ancestral state reconstruction of stridulatory systems used in the script "SCRIPT_ASR.R".

DATA_Acoustic_Features_Imputed_(fromTableS8).csv

Description: Imputed acoustic data used for UMAP clustering and analysis. The data in this file consists of acoustic parameters measured from live hawkmoths during stridulatory behavior assays. See the README for Table S7 for detailed descriptions of the acoustic parameters listed.

DATA_for_PGLS_Scale_Perimeters_vs_Nodule_Diameters_(fromTableS13).csv

Description: This file contains mean measurements of scraper scale perimeters and T8 nodule diameters across sphingid genera.

DATA_for_Contmap_Scraper_Scale_Perimeters_(fromTableS13).csv

Description: This file contains mean measurements of scraper scale perimeters across sphingid genera.

DATA_for_Contmap_File_Nodule_Diameters_(fromTableS13).csv

Description: This file contains mean measurements of T8 nodule diameters across sphingid genera.

<u>Scripts:</u>

SCRIPT_ASR.R

Description: This annotated R script was used to conduct ancestral state reconstruction analyses of anti-bat acoustic response and sphingid stridulatory systems. This script relies on the tree file "Supplementary_Tree_File_1_Sphingidae_Dated_Tree_Couch_et_al._2026.tre" and the data files "DATA_for_ASR_Response_to_batcall_matrix_(fromTableS3).csv" and "DATA_for_ASR_Stridulatory_System_Matrix_(fromTableS6).csv" and produces raw versions of annotated phylogenies displayed in Figure 1.

SCRIPT_UMAP.R

Description: This annotated R script was used to cluster hawkmoth acoustic data using UMAP. This script relies on the data file "DATA_Acoustic_Features_Imputed_(fromTableS8).csv" and produces the raw plot displayed in Figure 3B.

SCRIPT_BOX_PLOTS.R

Description: This annotated R script was used to produce box plots for three specific acoustic features: Duty Cycle, Dominant Frequency, and Mean Duration of Modulation Cycle. This script relies on the data file “DATA_Acoustic_Features_Imputed_(fromTableS8).csv” and produces raw versions of the box graphs shown in Figure 3C, 3D, and 3E.

SCRIPT_PGLS.R

Description: This annotated R script was used to perform Phylogenetic Generalized Least Squares (PGLS) on scraper scale and file nodule measurements in a phylogenetic context. This script relies on the data file “DATA_for_PGLS_Scale_Perimeters_vs_Nodule_Diameters_(fromTableS13).csv” and the tree file “Supplementary_Tree_File_2_Sphingidae_Tree_Generic_Names.tre” and produces the PGLS regression graph displayed in Figure 4C.

SCRIPT_CONTINUOUS_TRAIT_MAPPING.R

Description: This annotated R script was used to map continuous traits (scraper scale perimeter and file nodule diameter) to a generic-level version of the sphingid tree. This script relies on the data files “DATA_for_Contmap_Scraper_Scale_Perimeters_(fromTableS13).csv” and “DATA_for_Contmap_File_Nodule_Diameters_(fromTableS13).csv” as well as the tree file “Supplementary_Tree_File_2_Sphingidae_Tree_Generic_Names.tre” and produces the raw versions of the trait-mapped trees displayed in Figures 4D+4E.

<u>Supplementary Tree Files:</u>

Supplementary_Tree_File_1_Sphingidae_Dated_Tree_Couch_et_al._2026.tre

Description: Dated tree of Sphingidae from Couch et al. (2026) derived from an anchored hybrid enrichment dataset of 537 loci.

Supplementary_Tree_File_2_Sphingidae_Tree_Generic_Names.tre

Description: Genus-level phylogeny of Sphingidae pruned from the dated tree of Couch et al. (2026).
